# A practical evaluation of AutoML tools for binary, multiclass, and multilabel classification

**DOI:** 10.1038/s41598-025-02149-x

**Published:** 2025-05-21

**Authors:** Marcelo V. C. Aragão, Augusto G. Afonso, Rafaela C. Ferraz, Rairon G. Ferreira, Sávio G. Leite, Felipe A. P. de Figueiredo, Samuel B. Mafra

**Affiliations:** National Institute of Telecommunications (Inatel), Santa Rita do Sapucaí, MG 37536-001 Brazil

**Keywords:** Machine learning, Classification, Hyperparameter optimization, Neural architecture search, AutoML, Computational science, Scientific data

## Abstract

Selecting the most suitable Automated Machine Learning (AutoML) tool is pivotal for achieving optimal performance in diverse classification tasks, including binary, multiclass, and multilabel scenarios. The wide range of frameworks with distinct features and capabilities complicates this decision, necessitating a systematic evaluation. This study benchmarks sixteen AutoML tools, including AutoGluon, AutoSklearn, TPOT, PyCaret, and Lightwood, across all three classification types using 21 real-world datasets. Unlike prior studies focusing on a subset of classification tasks or a limited number of tools, we provide a unified evaluation of sixteen frameworks, incorporating feature-based comparisons, time-constrained experiments, and multi-tier statistical validation. We also compared our findings with four representative prior benchmarks to contextualize our results within the existing literature. A key contribution of our study is the in-depth assessment of multilabel classification, exploring both native and label-powerset representations and revealing that several tools lack robust multilabel capabilities. Our findings demonstrate that AutoSklearn excels in predictive performance for binary and multiclass settings, albeit at longer training times, while Lightwood and AutoKeras offer faster training at the cost of predictive performance on complex datasets. AutoGluon emerges as the best overall solution, balancing predictive accuracy with computational efficiency. Our statistical analysis—at per-dataset, across-datasets, and all-datasets levels—confirms significant performance differences among tools, highlighting accuracy-speed trade-offs in AutoML. These insights underscore the importance of aligning tool selection with specific problem characteristics and resource constraints. The open-source code and reproducible experimental protocols further ensure the study’s value as a robust resource for researchers and practitioners.

## Introduction

Automated Machine Learning (AutoML), as the name suggests, automates the Machine Learning (ML) process, reducing the need for manual human labor and in-depth knowledge in the area^1^. Several AutoML tools—presented in this study—are available, but choosing one is not trivial since each has its particularities and is intended to solve different binary, multiclass, and multilabel classification problems. One of ML’s goals is to predict values or categories for new (i.e., unseen) data as accurately as possible. Thus, selecting an AutoML tool is essential, particularly when balancing model performance and training efficiency. Given the rapid industrial uptake of AutoML, the community still lacks a single “all-tasks” benchmark that is both statistically rigorous and reproducible.

Despite intense activity, we found *no* prior study that (i) validates results at three nested levels–per-dataset, across-datasets, and all-datasets—and (ii) contrasts *native* versus *label-powerset* multilabel handling. As summarised in Table 2, even the largest benchmarks (Truong et al.^2^, Wever et al.^3^, and Gijbers et al.^4^) omit one or both facets, making their findings hard to generalise. Specifically, Truong et al. cover only binary and multiclass tasks; Wever et al. treat multilabel classification exclusively; and Gijbers et al. excludes multilabel datasets and reports only aggregate scores without per-dataset or corpus-wide significance testing. In contrast, our work evaluates sixteen widely used Python frameworks on twenty-one real-world datasets that span *all three* classification types, applies the above three-tier statistical analysis, and is therefore, to the best of our knowledge, the most comprehensive AutoML benchmark to date.

Our contributions are fourfold: (1) the first systematic AutoML comparison that jointly covers binary, multiclass, native multilabel, and powerset multilabel tasks; (2) a tight 5-min, hardware-controlled experimental design with standardised weighted-$F_1$ and timing metrics; (3) a comprehensive multi-layer validation pipeline (per-dataset, across-datasets, all-datasets) that quantifies accuracy–speed trade-offs with robust significance tests; and (4) the public release of *all* code, including the end-to-end statistical scripts, so researchers can replicate or extend every figure and table (see Code availability). Crucially, these scripts allow the entire statistical analysis to be rerun with ease.

This paper is organized into seven parts: “Theory review” section explains the main concepts necessary for understanding the article; “Related work” section reviews prior studies on AutoML, positioning our research within existing literature; “Assessment methodology” section presents the work proposal, containing details of the steps performed and why only classification (and not regression) problems were addressed; “Experiments” section describes the experiments conducted, as well as the results obtained and in-depth analysis (quantitative and qualitative) of these results, including benchmarking our findings against four representative prior studies; “Statistical analysis” section provides an extensive statistical validation of the results, employing multi-tier significance tests to compare the performance of AutoML frameworks at different levels; “Threats to validity” section discusses potential limitations of the study, addressing internal, external, and construct validity concerns; and finally, “Conclusion” section presents the main findings derived from this work and proposes future research directions.

## Theory review

The comparative study proposed in this paper addresses several areas of study, such as machine learning, hyperparameter optimization, neural architecture search, and AutoML. A brief theoretical review of each area follows.

### Machine and supervised learning

Machine learning (ML) is a significant domain within Artificial Intelligence (AI) that enables computer systems to learn from data rather than being explicitly programmed. In supervised learning, a subset of ML, systems use labeled datasets during training to produce models that can classify new, unseen data based on patterns learned from the training data^5^.

Supervised learning methods are designed to solve classification and regression tasks. This work focuses on classification, where the objective is to assign input data to predefined categories based on the learned patterns. Classification tasks include:

- *Binary classification* Involves classifying data into two mutually exclusive categories, such as determining whether an email is spam or non-spam based on word frequency and structure or predicting if a patient has a disease or is healthy based on medical test results like blood pressure and cholesterol levels.
- *Multiclass classification* Assigns each sample a single label from a set of more than two mutually exclusive categories, such as classifying animal images as cats, dogs, or birds based on physical features or categorizing movies into genres like drama, comedy, or action based on themes, plot structures, and viewer preferences.
- *Multilabel classification* Associates each sample with one or more labels simultaneously, such as tagging an image with “beach,” “sunset,” and “vacation” to describe its multiple attributes, or assigning research keywords like “machine learning,” “AI,” and “data science” to a paper to reflect its diverse topics and focus areas.

**Fig. 1:**
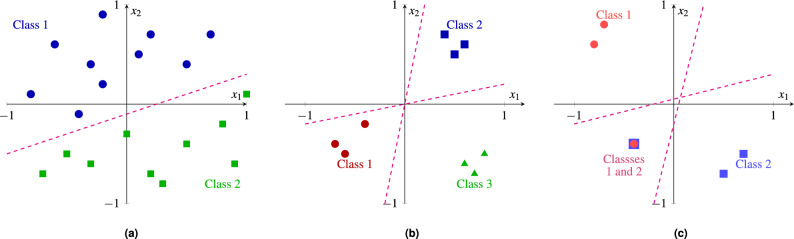
Binary, multiclass, and multilabel classification examples.

The key difference among these tasks lies in the complexity of the output: binary handles two classes (Fig. 1a), multiclass assigns one label from many (Fig. 1b), and multilabel allows multiple labels per sample (Fig. 1c), often requiring models to capture label interdependencies.

In addition, several algorithms widely discussed in the literature are often associated with solving specific tasks. For instance, binary classification tasks frequently utilize algorithms such as Support Vector Machines (SVMs)^6^ and logistic regression^7^. Multiclass classification often employs decision trees^8^, *k*-nearest neighbors (KNN)^9^, or neural networks^10^. For multilabel classification, specialized algorithms such as Binary Relevance (BR)^11^, Classifier Chains (CC)^12^, Label Powerset (LP)^13^, or adaptations of neural networks^14^ are commonly used. The AutoML tools discussed in this paper leverage this knowledge to automate the selection and optimization of ML models or pipelines, enhancing efficiency and reducing the need for extensive manual intervention.

### Hyperparameter optimization (HPO)

ML models require not only the learning of parameters during training but also the careful selection of predefined settings called hyperparameters. Training algorithms work by iteratively adjusting model parameters, such as weights in a neural network, to minimize a loss function and improve performance on the training data^15^. Hyperparameters govern the behavior of this process and can include values such as the learning rate, the number of layers in a neural network, or the depth of a decision tree. Well-chosen hyperparameters can significantly improve model performance and computational efficiency.

Hyperparameter optimization (HPO) aims to identify the best configuration of these settings to maximize the model’s performance, often measured using a validation metric such as accuracy or $F_1$ score. Unlike parameters, which are adjusted dynamically during training, hyperparameters must be determined before training begins. The HPO process involves defining a search space of potential configurations, evaluating their effectiveness, and iteratively refining the search to approach the optimal solution^16,17^.

**Fig. 2:**
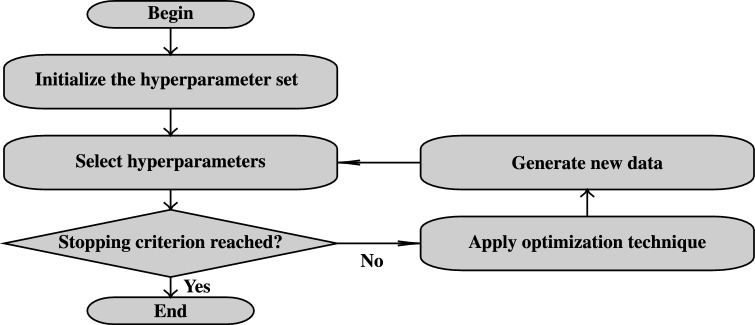
General hyperparameter optimization flow chart. Adapted from Loka et al.^19^ and Nguyen et al.^20^.

Figure 2 illustrates the general workflow of HPO, emphasizing its iterative nature. The problem setup includes selecting an evaluation metric, defining the range of potential values for each setting, and determining how these configurations will be assessed during the search process.

Methods for exploring the search space can be broadly categorized into manual and automated approaches. Manual methods rely on user expertise and intuition, often requiring extensive trial and error. Automated methods, in contrast, utilize algorithms to evaluate configurations systematically and will be discussed in detail in the remainder of this section^18^.

**Fig. 3:**
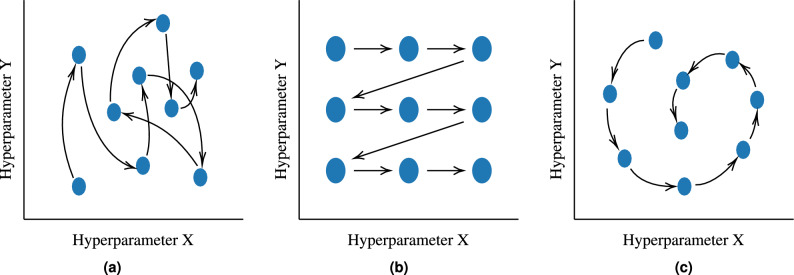
Random Search, Grid Search, and Bayesian Optimization. Adapted from Chen^22^.

Random Search (Fig. 3a) and Grid Search (Fig. 3b) can be used for both classification and regression. Both models use cross-validation and two parameters: the model with the hyperparameter values to be optimized and the search space^16^. The difference between them is in the search space definition, where Random Search tests several hyperparameter settings, and Grid Search exhaustively tests all settings (i.e., all possible combinations)^21^.

Bayesian optimization aims to deduce black-box functions that are initially unknown and usually have a high computational cost to be solved. The Bayesian strategy (Fig. 3c) combines information from the unknown hyperparameter function with sample data to obtain information that will be used to deduce the optimal values of the function. In addition, Gaussian processes are also used to fit the high and low point distributions. Finally, the entire process is repeated until the maximum number of iterations is reached or the difference between the optimal and current values is less than a specified threshold^17^.

**Fig. 4:**
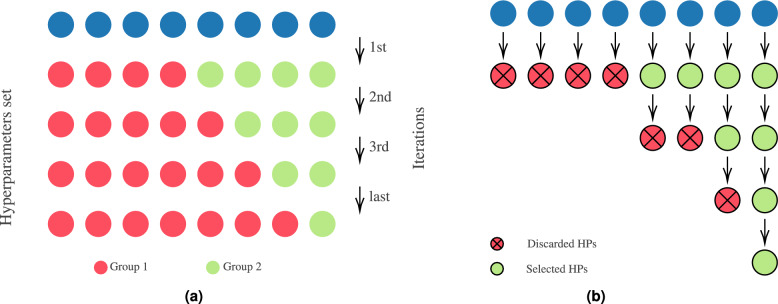
Tree-structured parzen estimator and Hyperband. Adapted from Nguyen et al.^20^ and Li et al.^23^.

The TPE algorithm is commonly used in various domains, including image processing, solar radiation prediction, and work accidents. It employs a Gaussian distribution to model hyperparameter values based on experimental data. Random search is performed initially to initialize the response surface sampling distributions. The hyperparameter space is divided based on fitness values and a predefined threshold, as depicted in Fig. 4a. The optimal hyperparameters are determined from the best observations. Increasing the number of initial iterations improves the distribution^20^.

Unlike other algorithms, Hyperband adopts an early-stopping strategy and requires two inputs to determine the resource allocation. The first input specifies the maximum allocated resources for a configuration, while the second controls the proportion of configurations discarded during successive halving (see Fig. 4b). This approach allows Hyperband to evaluate more configurations in high-cost problems without making strong assumptions. The algorithm begins aggressively to maximize exploitation and ensures that at least one configuration receives resources. Subsequent iterations decrease the minimum resource allocation based on the defined proportion until the final iteration employs Random Search. Hyperband can adaptively allocate resources and may favor conservative allocations in certain scenarios^23^.

The process of optimizing hyperparameters encompasses a range of techniques applicable to various machine learning models. These models, along with some of their respective hyperparameters, include (but are not restricted to):

- *Decision trees (DTs)* maximum depth limits tree complexity to prevent overfitting, while minimum samples per leaf ensure splits are meaningful and not based on small, noisy subsets.
- *K*-*Nearest neighbors (KNN)* number of neighbors (*k*) determines decision boundary smoothness; small *k* captures fine details but risks overfitting, while large *k* smooths predictions at the cost of local precision.
- *Neural networks (NNs)* learning rate governs training speed and stability, with smaller values ensuring steady convergence. Hidden layers and neurons affect model complexity, enabling simple to intricate pattern recognition.
- *Random forests (RFs)* number of trees enhances stability by averaging predictions, while maximum features per split control tree diversity, balancing accuracy and robustness.
- *Support vector machines (SVMs)* regularization parameter (*C*) controls the trade-off between overfitting and generalization, with smaller *C* enforcing simpler models. Kernel type (e.g., linear or polynomial) maps data into higher-dimensional spaces for separating non-linear patterns.

While manually tuning these hyperparameters can be a tedious and time-consuming process, automated hyperparameter optimization contributes significantly to AutoML by reducing the need for human effort, improving the performance of ML algorithms, and being more accessible to reproduce than manual configuration^24^. However, some difficulties are noted, such as the high cost of evaluations when the models, datasets, or pipelines are complex and the lack of clarity about which hyperparameter needs optimization and which ranges should be optimized.

### Neural architecture search (NAS)

Deep learning’s ability to automatically extract features has revolutionized machine learning^25^, shifting the focus to designing effective neural architectures—the blueprints for network structure. Hyperparameters define these architectures, such as the number of hidden layers and neurons within each layer. During training, the network’s internal parameters (weights and biases) are iteratively adjusted through gradient-based optimization techniques like backpropagation^26^ to minimize a loss function. Manually designing neural architectures is labor-intensive and requires significant expertise.

While gradient-based optimization excels at refining a neural network’s parameters through backpropagation^26^, Neural Architecture Search (NAS) tackles a more fundamental challenge: automatically identifying the hyperparameters that optimize the network architecture itself^27^.

The success of deep learning hinges on a well-designed neural network architecture. However, manually crafting these architectures is a highly specialized and time-consuming task. Neural Architecture Search (NAS) addresses this challenge by automating the discovery of optimal architectures^27^.

**Fig. 5:**
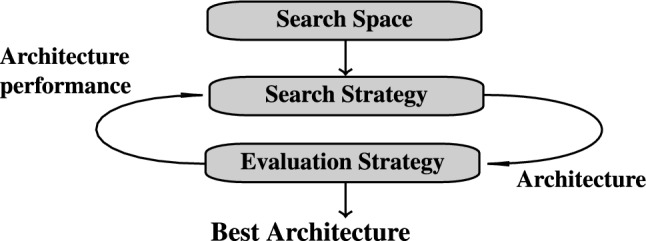
General NAS methodology. Adapted from Elsken et al.^27^.

As shown in Fig. 5, the NAS methodology typically consists of three main components:

- *Search space* The set of possible building blocks, such as layer types (e.g., fully connected, convolutional, pooling), activation functions, and other structural elements. A well-defined search space balances flexibility with computational feasibility, enabling the discovery of meaningful architectures.
- *Search strategy* The method used to navigate the search space and generate promising architectures. Strategies range from simpler approaches like random search to advanced techniques such as evolutionary algorithms. The choice of strategy affects the speed and quality of the search, with trade-offs between exploration and computational cost.
- *Evaluation strategy* The process of assessing a candidate architecture’s performance, typically using metrics like accuracy or loss on a validation set. Results guide the search toward better architectures, balancing efficiency and accuracy to impact both search effectiveness and the quality of the final results.

As mentioned earlier, the search strategy is critical in NAS as it determines how the search space is explored. Two common strategies are:

- *Evolutionary optimization* Inspired by biological evolution, this approach generates a population of candidate architectures and evaluates their performance. High-performing architectures are combined and mutated to produce new candidates in iterative cycles, gradually leading to improved designs^28^.
- *Random search* This simpler approach randomly samples architectures from the search space, evaluating each using the chosen evaluation strategy (e.g., accuracy on a validation set). After a set number of iterations, the architecture with the best performance is selected. Despite its simplicity, random search can be surprisingly effective, particularly in smaller search spaces^29^.

Traditional NAS approaches separate the search and evaluation phases, which can be computationally expensive. One-shot methods streamline the process by integrating these phases into a single step to overcome these challenges, significantly reducing computational costs. Two notable one-shot NAS algorithms are:

- *Efficient neural architecture search (ENAS)* Treats all possible architectures as sub-graphs of a larger supergraph. Each node in the supergraph contains several candidate operations. Once an architecture is trained, its weights are shared with other architectures that share edges in the supergraph^30^.
- *Differentiable architecture search (DARTS)* Relaxes each node’s choice of activation functions into a softmax distribution over all possible operations. This allows the search space to be optimized continuously by minimizing both validation and training loss^31^.

NAS can be an integral step within an AutoML framework, enhancing the automation of the machine learning pipeline by selecting neural network architectures. The concept of AutoML, including its integration with NAS, will be explained in the next subsection.

### Automated machine learning (AutoML)

As previously mentioned, ML is an assertive discipline of AI that can solve many issues by recognizing patterns or making data-based predictions. However, it requires human knowledge, interference, and effort from data scientists.

Automated ML was born to reduce these and other inconveniences. According to Ref.^32^, AutoML may reduce numerous manual tasks and permit domain experts to create and work well with ML pipelines without advanced expertise in ML or statistics. Reference^33^ sees AutoML as a conjunction of automation and ML. They say that AutoML highlights the facility of configuration and control of ML learning tools, making it self-adaptive to problems.

To explore AutoML’s potential, we selected frameworks based on popularity, community adoption, features, and Python support. Most are open source and actively maintained on GitHub, ensuring accessibility and transparency. To provide a broader view, we included 4intelligence, a commercial framework. These frameworks were chosen for their ability to automate key stages of the ML pipeline for structured data. Below, we outline their main features and capabilities.

- 4intelligence^34^: A cloud-based framework by 4intelligence for binary and multiclass classification, requiring minimal user input. It automates data cleaning, feature engineering, preprocessing, and model selection using various algorithms.
- AutoGluon^35^: An open-source AutoML toolkit with deep learning capabilities for tabular, image, and text data tasks. Automates major steps in the ML process, making it accessible for users with diverse skill levels.
- AutoKeras^36^: An open-source system that leverages CPUs and GPUs to train neural networks, focusing on image and text classification and regression tasks. Employs Keras and Bayesian optimization for efficient neural architecture search (NAS) with a user-friendly interface that requires no deep learning expertise.
- Auto-PyTorch^37^: Built on PyTorch, this framework automates NAS and HPO for optimal neural networks, primarily for tabular data tasks. It streamlines the development of models for classification and regression problems.
- AutoSklearn^38^: Based on scikit-learn, it offers diverse preprocessing methods and supports classification, regression, and multilabel tasks. Incorporates meta-learning to enhance Bayesian optimization efficiency and reduce training time.
- EvalML^39^: Automates the entire ML pipeline for structured data, including data loading, cleaning, feature engineering, model selection, HPO, and model evaluation, enabling model building with reduced coding effort.
- FEDOT^40^: An AutoML framework designed for complex ML tasks, including classification. It employs evolutionary algorithms to optimize pipeline creation, focusing on interpretable results to support data-driven decision-making.
- FLAML^41^: A lightweight AutoML library that prioritizes efficient, cost-effective search for accurate models by optimizing the search process to minimize computational costs beyond evaluating each configuration.
- GAMA^42^: An open-source AutoML framework that optimizes pipelines for supervised tasks using genetic programming, offering an intuitive interface for model selection and HPO in classification and regression.
- H2O^43^: Optimized for scalable ML, supporting efficient parallel training of multiple models on large datasets. Aims to achieve competitive performance by utilizing distributed algorithms designed for high efficiency.
- LightAutoML^44^: A comprehensive, open-source framework emphasizing transparency in ML. Automates data preparation, feature engineering, model selection, HPO, and model deployment, ensuring interpretability in predictions.
- Lightwood^45^: An open-source AutoML library developed by MindsDB, focusing on integrating machine learning models directly within databases. It automates core processes like data preprocessing and feature selection for structured data, emphasizing accessibility and ease of use.
- mljar-supervised^46^: Dedicated to supervised learning tasks, focusing on both accuracy and interpretability. Automates model selection, HPO, and training with insights into model performance and feature importance.
- NaiveAutoML^47^: A lightweight AutoML tool designed for simplicity, automating tasks like data cleaning, feature selection, and basic model evaluation. Ideal for interpretable applications with minimal setup.
- PyCaret^48^: A low-code Python library simplifying the ML workflow. Automates preprocessing, feature engineering, model selection, hyperparameter tuning, and evaluation, supporting classification, regression, clustering, and anomaly detection, and seamlessly integrates with popular deployment tools.
- TPOT^49^: Uses genetic programming to generate and refine ML pipelines automatically. Manages data preprocessing, feature selection, and model selection with minimal input, providing user-friendly and modifiable pipelines.

It is shown in works like^50^, which uses real-life datasets, how helpful AutoML is, making it possible for people without broad knowledge to operate datasets and make a difference with its creation. “Related work” section will further confirm the potential and user-friendliness of AutoML tools through a comprehensive analysis of existing works.

In addition, Table 1 summarizes the key features and capabilities of each AutoML framework, such as data handling (missing data, duplicates, noisy data, outliers), categorical data management, scaling, normalization, feature engineering, NAS, and supported classification types. This comparison will provide insights into the strengths and limitations of each framework, aiding in informed decision-making for researchers and practitioners.

Thus, this paper uses the domains presented above and their characteristics as categories of comparison between the AutoML tools studied to provide detailed insights into how each tool addresses these areas.

**Table 1:** Frameworks summary.

(a) Part 1: AutoGluon, AutoKeras, Auto-PyTorch, AutoSklearn, EvalML					
Feature/capability	Frameworks (Part 1)				
	AutoGluon	AutoKeras	Auto-PyTorch	AutoSklearn	EvalML
Handling missing data	Yes	No	Yes	Yes	Yes
Dealing with duplicates	No	No	No	No	No
Handling noisy data	No	No	No	No	No
Handling outliers	No	No	No	No	No
Handling categorical data	Yes	Yes	Yes	Yes	Yes
Scaling and normalization	Yes	Yes	Yes	Yes	Yes
Handling multicollinearity	No	No	No	No	No
Feature engineering	Yes	Limited	Yes	Yes	Yes
Neural architecture search	No	Yes	Yes	No	No
Data reduction	Yes	No	Yes	Yes	Yes
Data type support	Image, tabular, text	Image, tabular	Image, tabular	Tabular	Tabular, text
Binary classification	Yes	Yes	Yes	Yes	Yes
Multiclass classification	Yes	Yes	Yes	Yes	Yes
Multilabel classification	Limited	Yes	No	Yes	No
Underlying models	EM, NN, Other (TabNet)	Primarily CNNs	CNNs, feedforward NNs	Ensemble methods (scikit-learn)	Ensemble methods (multiple libraries)
Specific models	CNN, GB, LGBM, MLP, RF, TabNet, XGB	CNN (DenseNet, ResNet, VGG)	CNN (DenseNet, ResNet, VGG)	GB, KNN, Lasso, LR, NB, RF, Ridge, SVM	CatBoost, KNN, LGBM, SVM, XGBoost
HPO Strategy	Random + Hyperband	Bayesian + NAS	Bayesian + BOHB	Bayesian (SMAC) + Meta	Bayesian/grid
Runtime overhead	Moderate	High	High	High	Moderate
Scalability support	Multi-core/GPU	Multi-GPU	Multi-core	Multi-core	Multi-core
Integration ease	Low-code API	Keras-style API	PyTorch API	sklearn-style API	Low-code API
Interpretability features	SHAP, permutation importance	Saliency maps, Grad-CAM, LIME	Captum (integrated gradients, guided GradCam)	Permutation importance; interpretable-model subsets	SHAP, LIME

(b) Part 2: FEDOT, FLAML, GAMA, H2O, LightAutoML					
Feature/capability	Frameworks (Part 2)				
	FEDOT	FLAML	GAMA	H2O	LightAutoML
Handling missing data	Yes	Yes	No	Yes	Yes
Dealing with duplicates	No	No	No	No	No
Handling noisy data	Yes	No	No	Yes	Yes
Handling outliers	Limited	No	No	Yes	No
Handling categorical data	Yes	Yes	No	Yes	Yes
Scaling and normalization	Yes	Yes	No	Yes	Yes
Handling multicollinearity	Limited	No	No	Yes	No
Feature engineering	Yes	Yes	Yes	Yes	Yes
Neural architecture search	Limited	No	No	No	No
Data reduction	Yes	No	No	Yes	Yes
Data type support	Image, tabular, text, time series	Tabular	Tabular	Tabular, Text	Tabular
Binary classification	Yes	Yes	Yes	Yes	Yes
Multiclass classification	Yes	Yes	Yes	Yes	Yes
Multilabel classification	No	No	No	No	No
Underlying Models	Ensemble methods, hybrid	Ensemble methods	Ensemble methods	Ensemble methods (GBMs)	Ensemble methods, NN
Specific models	GB, Linear, NN, RF, XGB	LGBM, RF, XGB	GB, LR, RF, Ridge	DL, ET, GLM, RF, Stacked Ensembles, XGB	LGBM, NN, XGB
HPO strategy	Evolutionary	Cost-aware Bayesian	GP	Grid + Random	Preset recipes
Runtime overhead	High	Low	High	High	Moderate
Scalability support	Multi-core	Multi-core	Multi-core	Distributed cluster	Multi-core
Integration ease	JSON config API	Single-class API	CLI + Python	Java/Python REST	Low-code API
Interpretability features	None (external: SHAP, LIME, Evidently)	Permutation importances, SHAP	None (external: SHAP, LIME)	PDP, ICE, SHAP, surrogate trees, LOCO	Feature importance, PDP, ICE, AutoWoE

(c) Part 3: Lightwood, mljar-supervised, NaiveAutoML, PyCaret, TPOT					
Feature/capability	Frameworks (Part 3)				
	Lightwood	mljar-supervised	NaiveAutoML	PyCaret	TPOT
Handling missing data	Yes	Yes	Limited	Yes	Yes
Dealing with duplicates	No	No	No	No	No
Handling noisy data	No	No	No	No	No
Handling outliers	No	No	No	No	No
Handling categorical data	Yes	Yes	Limited	Yes	Yes
Scaling and normalization	Yes	Yes	No	Yes	Yes
Handling multicollinearity	Limited	Limited	No	Yes	No
Feature engineering	Yes	Yes	No	Yes	Yes
Neural architecture search	No	No	No	No	No
Data reduction	Limited	Yes	No	Yes	Yes
Data type support	Tabular, time series	Tabular	Tabular	Tabular	Tabular
Binary classification	Yes	Yes	Yes	Yes	Yes
Multiclass classification	Yes	Yes	Yes	Yes	Yes
Multilabel classification	No	No	No	No	No
Underlying models	NN, RNN	Ensemble Methods	Scikit-learn Estimators	Integrations (various)	Ensemble Methods
Specific models	NN, RNN	CatBoost, ET, LGBM, RF	DT, KNN, LR, NB, RF, SVM	Integrations (various)	GB, LR, RF, ridge
HPO strategy	Minimal random	Optuna random	None/minimal	Optuna/Bayesian	GP
Runtime overhead	Low	Moderate	Low	Moderate	Very high
Scalability support	Single-core	Multi-core	Single-core	Multi-core	Multi-core
Integration ease	SQL/JSON friendly	Web UI + Python	One-liner API	Low-code workflow	sklearn-style API
Interpretability features	Permutation importance	Markdown reports, local/global attributions	None	SHAP, LIME, PDP/ICE	None

Note that the table reflects each AutoML framework’s native, out-of-the-box features and capabilities and does not include custom algorithms or implementations. Thus, cells marked as “Limited” indicate partial or constrained support within the framework’s native capabilities.

Acronyms used in the table: *AutoWoE* automatic weight-of-evidence, *BOHB* Bayesian optimization and hyperband, *CatBoost* categorical boosting, CLI command–line interface, *CNN* convolutional neural network, *DL* deep learning, *DT* decision tree, *EM* ensemble methods, *ET* extra trees, *GB* gradient boosting, *GLM* generalized linear models, *GP* genetic programming, *HPO* hyperparameter optimization, *ICE* individual conditional expectation, *JSON* JavaScript object notation, *KNN* K-nearest neighbors, *Lasso* least absolute shrinkage and selection operator, *LGBM* light gradient boosting machine, *LIME* Local interpretable model-agnostic explanations, *LOCO* leave-one-covariate-out, *LR* logistic regression, *MLP* multi-layer perceptron, *NAS* neural architecture search, *NB* Naive Bayes, *NN* neural network, *PDP* partial dependence plot, *REST* representational state transfer, *Ridge* ridge regression, *RNN* recurrent neural network, *SHAP* SHapley Additive exPlanations, *SMAC* Sequential model-based algorithm configuration, *SVM* support vector machine, *XGB* eXtreme gradient boosting

## Related work

This section situates our research within AutoML. “Overview of existing research” section reviews existing studies on frameworks, surveys, and applications, while “Research gap and our contribution” section identifies research gaps and outlines our contributions through benchmarking and in-depth statistical analysis.

### Overview of existing research

We review literature on AutoML, examining its functionalities, applications, and evaluations. Our synthesis highlights assessment methodologies, metrics, scalability challenges, and the transition from domain-specific to general-purpose benchmarking.

Truong et al.^2^ compared AutoML tools across datasets, assessing their performance, advantages, and limitations. While AutoML tools excel in feature engineering, data preprocessing still requires significant human intervention. The evaluation involved around 300 OpenML datasets, with accuracy as the primary metric for binary and multiclass classification tasks. It was observed that no single tool consistently outperformed others across diverse tasks, indicating performance variations depending on the specific test. Notably, H2O excelled in binary classification, while AutoKeras led in multiclass classification.

Wever et al.^3^ advanced the study of AutoML by focusing on multilabel classification. They introduced a benchmarking framework ensuring uniform runtime constraints, search space models, and evaluation routines across multiple optimization approaches, including Bayesian optimization and bandit algorithms. Their results underscored the difficulties posed by large search spaces and found that a grammar-based best-first search approach outperformed others for multilabel tasks.

Mustafa and Azghadi^51^ surveyed AutoML in clinical notes analysis, highlighting its challenges and advantages in healthcare. The most commonly used algorithms for clinical notes analysis were Support Vector Machine, Convolutional Neural Networks, Random Forest, and Linear Regression. It was noted that no single feature selection algorithm consistently provided optimal results across all datasets. Future research suggests exploring new feature-extraction techniques, comparing feature selection methods, and developing tools combining Bayesian optimization with random search.

Similarly, Bahri et al.^52^ reviewed AutoML techniques for unsupervised anomaly detection. The review addresses challenges in building high-quality machine learning models through AutoML, emphasizing scalability, evaluation metrics, and handling high-dimensional data. The methodology involves summarizing representative methods, evaluating their performance, and discussing their advantages and disadvantages. Additionally, various automated methods and strategies for anomaly detection are reviewed, including meta-learning approaches and meta-features. Results include insights into the current state of AutoML, highlighting limitations and open research questions, along with updated overviews of AutoML and HPO methods. Furthermore, the article examines anomaly detection algorithms, highlighting common uses, challenges like hyperparameter sensitivity, and frameworks such as PyOD and PyODDS for scalability and pipeline optimization.

In addition to domain-specific applications, AutoML has shown promise in augmenting model training processes. Lin et al.^53^ developed a solution to optimize augmentation policies during network training. The solution, named OHL-Auto-Aug, treated the augmentation policy as a parameterized probability distribution, and its strategy for neural architecture search was based on AutoML techniques. It was formulated as a bilevel framework where, in the inner loops, the network parameters were trained using a standard Stochastic Gradient Descent along with augmentation sampling. In the outer loops, augmentation distribution parameters were trained using REINFORCE gradients with trajectory samples. The solution achieved a search efficiency 60x faster on CIFAR-10 and 24x faster on ImageNet than the previous state-of-the-art approach.

Extending AutoML’s application across diverse domains, Angarita-Zapata et al.^54^ conducted a study on hybrid AutoML for traffic forecasting, focusing on using the AutoSklearn framework. The study explored three scenarios: optimization, meta-learning, and ensemble learning; meta-learning combined with ensemble learning; and selecting the best-performing pipeline suggested by meta-learning. The study used speed measurements at 5-min intervals for highways and 15-min intervals for urban areas. AutoSklearn assumes pipelines ranked near position 1, with meta-features closest to the input dataset, perform better. However, the distance measure showed a weak correlation with performance, and pipelines below position 25 were excluded based on this similarity metric.

AutoML’s impact on medical imaging was explored by Beduin^50^, who proposed a new residual model called AutoResCovidNet using AutoKeras. This model aimed to compare X-ray images of healthy, pneumonia, and COVID-19-diagnosed individuals. The chosen metrics for analysis were accuracy, precision, sensitivity, and $F_1$ score, with parameters designed to enhance image processing accuracy. Before AutoML, preprocessing was performed on the COVIDx-m dataset to optimize the training data. Despite testing 20 models, AutoResCovidNet did not surpass existing results in the literature. However, the study highlighted the advantages of AutoML in accelerating model development and enabling focused research for more specific and satisfactory outcomes. The user-friendly interface, simplicity, and strong processing power of AutoKeras were recognized.

Expanding on domain-specific applications, van Eeden et al.^55^ compared multinomial logistic regression, Naïve Bayesian classifier, and AutoSklearn for predicting psychiatric diagnoses. They hypothesized that AutoSklearn would outperform the Bayesian classifier, surpassing traditional regression techniques in detecting complex patterns. They also hypothesized that AutoSklearn would be efficient when including single items and follow-up measures. The study used data from the Netherlands Study of Depression and Anxiety (NESDA), excluding non-Dutch fluent patients and other psychiatric disorders. Variables such as gender, age, ethnicity, education level, relationship status, and employment status were considered. AutoSklearn outperformed logistic regression and the Bayesian classifier on more complex predictor sets, but its accuracy varied depending on the predictor variables used. However, AutoSklearn was the most consistent across predictor sets.

Beyond specific applications, Ferreira et al.^56^ proposed a benchmark study to examine the characteristics of eight open-source AutoML frameworks (AutoKeras, Auto-PyTorch, AutoSklearn, AutoGluon, H2O, TPOT, rminer, and TransmogrifAI) along with twelve popular OpenML datasets (such as 37—Diabetes and 23—Contraceptive Method Choice). These datasets were divided into regression, binary, and multiclass classification tasks. The study compared the performance of these tools across different scenarios: General Machine Learning (GML), Deep Learning (DL), and XGBoost (XGB). In the GML scenario, results indicated that TransmogrifAI excelled in binary classification, AutoGluon in multiclass classification, and rminer in regression tasks. In the DL scenario, H2O performed well in binary classification and regression tasks, while AutoGluon stood out in multiclass classification. For XGB, rminer was the top choice for binary classification and regression tasks, and H2O for multiclass classification. Overall, the GML approach demonstrated superior predictive performance, with tools like TransmogrifAI, AutoGluon, and rminer consistently delivering strong results across tasks.

Romero et al.^57^ evaluated the performance of three widely used AutoML frameworks—AutoSklearn, H2O, and TPOT—on highly imbalanced binary classification tasks in the healthcare domain. Using de-identified medical claims data, they predicted the occurrence of six different diseases, each framed as a separate binary task. Their evaluation focused on imbalanced-aware metrics, such as the Area Under the Precision-Recall Curve (AUPRC), due to the low prevalence of positive cases. While all AutoML tools outperformed a tuned Random Forest baseline, their relative performances were not statistically distinguishable, suggesting no single framework had a consistent advantage across tasks. This study highlights the need to evaluate AutoML tools under real-world data imbalance, especially in high-stakes domains like healthcare.

Synthesizing these insights, Del Valle et al.^58^ systematically reviewed AutoML for multilabel classification and multi-target regression. The review analyzed existing AutoML approaches, encompassing search space definition, optimization algorithms, and evaluation metrics while identifying limitations and proposing future research directions. The protocol involved selecting studies from various online data sources, employing a four-step process: submitting a search string, initial and final selection, and snowballing. Initially identifying 94 studies, the review ultimately selected 12 for analysis, which included models such as convolutional networks and grammar-based genetic programming. Key findings include determining suitable evaluation measures for each context, exploring alternative loss functions, enhancing search space definition, developing methods for large datasets (e.g., transfer learning), and investigating meta-learning approaches to expedite the AutoML search process.

Neverov et al.^59^ applied AutoML to wave data classification, addressing parameter optimization challenges. The authors analyzed frameworks including MLJAR AutoML, AutoGluon, AutoKeras, and TPOT, evaluating performance on datasets like Sonar, Doppler, and Winnipeg, featuring energy bands, radar matrices, and labels like mine/rock, car/human/drone, and crops. Using genetic algorithms and Bayesian optimization, AutoGluon achieved the highest accuracy, outperforming other frameworks. The study also demonstrated AutoGluon ’s ability to combine models and optimize performance, making it faster, more reliable, and accurate, underscoring AutoML’s effectiveness in wave data classification and real-world applicability.

Salehin et al.^60^ expanded on AutoML’s broader implications by conducting a comprehensive review of AutoML and Neural Architecture Search (NAS). They analyzed existing literature and provided insights into advancements, challenges, and future directions. Employing a systematic review approach, the authors identified studies from reputable databases. The review covers various aspects, including AutoML methods, industrial applications, and open issues related to performance and accuracy. It highlights the role of these techniques in optimizing deep neural applications and addressing challenges in accuracy, latency, and energy consumption. Additionally, the article discusses the motivation behind AutoML research and its potential impact on innovation across industries. It also examines open issues and limitations, offering insights into potential developments.

To systematically evaluate and compare the performance of various AutoML frameworks, Gijbers et al.^4^ introduced Automated Machine Learning Benchmark (AMLB), a comprehensive benchmarking framework. AMLB provides a standardized environment with a diverse collection of real-world datasets covering tasks such as classification and regression of varying complexities. By establishing consistent evaluation metrics and protocols, curating datasets from multiple domains to test generalization capabilities, presenting empirical results of state-of-the-art AutoML frameworks, and offering an open-source platform to encourage reproducibility and future research, the authors emphasize the importance of such benchmarks in advancing AutoML development. AMLB is a valuable resource for researchers and industry professionals by identifying performance gaps and guiding practitioners in selecting appropriate tools to understand and improve AutoML technologies.

Eldeeb et al.^61^ proposed a large-scale AutoML benchmark study across 100 classification datasets using six frameworks, including AutoWEKA, AutoSklearn, TPOT, RECIPE, ATM, and SmartML. The study explored how pipeline design decisions—such as search space size, use of meta-learning, time budgets, and ensemble strategies—impact performance. Unlike many benchmarks that report only aggregate scores, Eldeeb et al. emphasized how tool behavior changes under different constraints. Although they did not focus on multilabel classification, their results provide a detailed comparative analysis of tool robustness and configurability across a wide range of tasks, offering practical guidance for AutoML deployment.

### Research gap and our contribution

This section has reviewed various AutoML frameworks, highlighting their transformative potential across diverse applications and methodologies. While these studies demonstrate significant advancements, challenges such as scalability, optimization for multilabel tasks, and the need for standardized benchmarks remain. Prior research has largely focused on theoretical comparisons, often failing to address real-world applications. Notably, although some works incorporate limited performance metrics (e.g., accuracy) or consider only binary and multiclass tasks, few studies adopt multi-level statistical tests or examine native and powerset multilabel approaches in a unified setting.

To bridge these gaps, our study evaluates sixteen AutoML frameworks under stringent time constraints across binary, multiclass, and multilabel problems—including both native and label-powerset classifications. Unlike many prior efforts, we employ extensive, multi-tiered statistical analyses (*per-dataset, across-datasets, and all-datasets*) to robustly determine significance in both predictive performance and computational efficiency. This hands-on methodology connects theoretical insights with real-world outcomes, offering practitioners actionable guidance for selecting or refining AutoML strategies. Our open-source code and reproducible protocols further ensure that researchers can replicate, extend, and apply our findings to a broad range of classification scenarios.

By evaluating AutoML frameworks using real datasets, our study provides a *broader, more practical* perspective on their capabilities, thus helping researchers and practitioners select the most appropriate tool based on classification type, computational constraints, and desired performance metrics. Table 2 highlights the key focuses, methods, metrics, and outcomes of prior research. It also outlines how our work addresses existing gaps – such as limited task diversity, lack of multilabel support, and insufficient statistical rigor—through multi-task benchmarking, multi-tier statistical analysis, and the explicit evaluation of both native and label-powerset multilabel strategies.

**Table 2:** Study-by-study summary of AutoML research, highlighting methods, findings, and distinctions from our work.

Reference	Focus	Methodology	Key metrics	Key results	Distinction from our work
Truong et al.^2^	AutoML tools for classification	Compared AutoML tools on 300 OpenML datasets for binary and multiclass tasks	Accuracy	H2O performed better in binary classification; AutoKeras excelled in multiclass classification	Focuses on accuracy alone; limited mention of multilabel, and does not use multi-level statistical tests
Wever et al.^3^	Multilabel classification	Proposed a benchmarking framework to compare Bayesian optimization, bandit algorithms, and hybrids for multilabel tasks	Performance under runtime constraints	Grammar-based best-first search performed best, overcoming optimization challenges in multilabel tasks	Explores multilabel but lacks broad classification coverage (binary, multiclass) and large-scale statistical comparisons
Mustafa and Azghadi^51^	AutoML in healthcare	Surveyed AutoML applications for clinical notes analysis. Discussed algorithmic challenges and feature extraction techniques.	Algorithm prevalence (e.g., SVM, CNN, RF, LR)	Suggested future research on feature selection methods and new extraction techniques	Concentrates on clinical text data; no end-to-end benchmark or multi-class scenario with extensive statistics
Bahri et al.^52^	Anomaly detection with AutoML	Reviewed techniques for anomaly detection using AutoML, focusing on scalability and high-dimensional data challenges	Scalability, evaluation metrics	Highlighted meta-learning approaches and frameworks like PyOD, emphasizing hyperparameter sensitivity issues	Centers on unsupervised anomaly detection; does not address binary/multiclass or label powerset
Lin et al.^53^	NAS	Proposed OHL-Auto-Aug, a bilevel NAS framework combining augmentation policy optimization with SGD and REINFORCE gradients.	Search efficiency, classification accuracy	Achieved 60x faster search on CIFAR-10 and 24x on ImageNet compared to prior approaches.	Focuses on neural architecture search in image tasks rather than comprehensive AutoML classification
Angarita-Zapata et al.^54^	Hybrid AutoML for traffic	Studied AutoSklearn in traffic forecasting with meta-learning, optimization, and ensemble learning scenarios.	Pipeline performance correlation	Found meta-features not well correlated with performance; pipelines beyond position 25 were disregarded	Domain-specific (traffic) approach with a single AutoML tool, no large-scale cross-framework comparison
Beduin^50^	AutoML in medical imaging	Developed AutoResCovidNet using AutoKeras for COVID-19 X-ray image classification	Accuracy, precision, sensitivity, $F_1$ score	Highlighted AutoML’s advantages despite AutoResCovidNet not surpassing prior results	Focuses on a single domain (medical X-ray), lacking multi-tool or multilabel analysis
van Eeden et al.^55^	Psychiatric diagnoses	Compared multinomial logistic regression, Naïve Bayesian classifier, and AutoSklearn for psychiatric diagnosis prediction	Accuracy across predictor sets	AutoSklearn outperformed traditional methods and showed consistent accuracy across predictor sets.	Domain-specific (psychiatry) with few tools; does not include extensive classification scenarios or time constraints.
Ferreira et al.^56^	AutoML benchmarking	Compared eight AutoML frameworks on 12 OpenML datasets under GML, DL, and XGB scenarios	Predictive performance, computational efficiency	GML: TransmogrifAI (binary), AutoGluon (multiclass), rminer (regression). DL: H2O and AutoGluon excelled.	Benchmarks multiple frameworks but less emphasis on multilabel or multi-tier statistical approaches
Romero et al.^57^	AutoML in healthcare	Benchmarked AutoSklearn, H2O, and TPOT on six imbalanced binary classification tasks derived from medical claims data	AUPRC, accuracy, $F_1$ score	All tools outperformed a Random Forest baseline, but no significant differences were found among them	Focused on real-world class imbalance; lacks multilabel, multi-class scenarios, or multi-tier statistical analysis
Del Valle et al.^58^	AutoML for multi-target learning	Systematic review analyzing AutoML for multilabel classification and multi-target regression	Search space definition, evaluation metrics	Proposed improvements in meta-learning, loss functions, and search space enhancements for large datasets	Comprehensive review on multilabel but not an experimental multi-framework benchmark with time constraints
Neverov et al.^59^	Wave data classification	Applied AutoML frameworks (e.g., AutoGluon, AutoKeras) on wave data classification using genetic algorithms and Bayesian optimization	Accuracy, model combination performance	AutoGluon achieved the highest accuracy and demonstrated robust model combination capabilities	Focuses on wave data domain; narrower scope, fewer frameworks, no multi-level statistical significance tests
Salehin et al.^60^	AutoML and NAS review	Reviewed advancements and challenges in AutoML and NAS, focusing on industrial applications and performance issues	Accuracy, latency, energy consumption	Identified key issues in optimizing deep neural applications and future directions for NAS	Literature-based review on NAS + AutoML synergy; not an extensive experimental benchmark or multi-task scenario
Gijbers et al.^4^	AutoML Benchmarking Framework (AMLB)	Designed AMLB to systematically evaluate and compare AutoML systems across tasks using standardized datasets and protocols	Generalization, benchmarking performance	Highlighted performance gaps and provided an open-source platform for reproducibility and research	Presents a valuable framework but does not specifically focus on multi-tier statistical methods or label-powerset strategies
Eldeeb et al.^61^	AutoML benchmark across 100 datasets	Compared six AutoML frameworks under varied design decisions (ensembling, meta-learning, time budgets)	Classification accuracy, runtime trends	Framework performance was sensitive to pipeline configurations; no tool was best in all settings	Large-scale benchmark of binary/multiclass tasks, but does not include multilabel or multi-level statistical validation

To complement the study-specific overview in Tables 2 and 3 presents a comparative summary of recent AutoML benchmarking efforts across key methodological dimensions. This feature-level synthesis highlights how our study extends the state of the art in terms of classification coverage, dataset diversity, statistical validation, and practical applicability.

**Table 3:** Feature-level comparison of AutoML studies, emphasizing task coverage, tool diversity, and evaluation rigor.

Feature/criterion	Truong et al.(2019)^2^	Wever et al.(2021)^3^	Romero et al.(2022)^57^	Gijsbers et al.(2024)^4^	Eldeeb et al.(2024)^61^	This study(2025)
Classification tasks	Binary multiclass	Multilabel	Binary	Binary multiclass	Binary multiclass	Binary Multiclass Multilabel
# Datasets	$\approx$ 300	10–15	1 (6 tasks)	71	100	21
Dataset overlap	11/21	5/21	0/21	6/21	6/21	–
# AutoML tools	14	Custom	3	9	6	16
Tool overlap	4/16	0/16	3/16	8/16	2/16	–
Statistical tests	✗	✓Wilcoxon ✓Friedman	✗	✓Friedman ✓Nemenyi	✗	✓ANOVA + Tukey’s HSD ✓Kruskal–Wallis + Dunn ✓Friedman + Nemenyi
Evaluation depth	Aggregated	Per-dataset aggregated	Task-wise	Per-dataset aggregated	Per-dataset config-aware	Per-dataset across-datasets all-datasets
Multilabel coverage	✗	✓Native	✗	✗	✗	✓Native ✓Label powerset
Dataset diversity	Medium	Medium	Low	Medium	High	High
Experimental rigor	Low	Medium	Medium	Medium	Medium	High
Notable contributions	Pioneered large-scale AutoML benchmark	First multilabel optimizer survey	First AutoML on medical claims	Introduced open AutoML benchmark	100-dataset AutoML study	Full-spectrum AutoML benchmarking Joint binary/multiclass/multilabel coverage Native + Label Powerset evaluation 21-dataset, 16-tool benchmark Three-level statistical validation
Open source code	✓	✓	✓	✓	✓	✓

*Dataset/tool overlap* values are shown as (matches / total), where the total number of datasets is reported in Table 4, and the total number of frameworks is listed in Table 1.

*Tool overlap* is based on the set of frameworks actually benchmarked in this study; minor variants or configurations are not counted separately unless independently evaluated.

*Statistical Tests*: ✓ indicates use of that test; ✗ indicates none.

*Evaluation Depth*: “Aggregated” = only overall metrics; “Per-dataset” = results per dataset; “Config-aware” = analysis of parameter settings.

*Dataset Diversity*: Low = single domain; Medium = few domains/types; High = broad variation (domain, size, complexity, imbalance).

*Experimental Rigor*: Low = single-run/no time budget; Medium = multiple runs or minimal controls; High = fixed time cap, multiple runs, hardware-controlled environment

## Assessment methodology

This section describes the methodology for evaluating AutoML tools in different classification tasks. It details dataset selection, data preprocessing, tool workflow, performance metrics, and result analysis to ensure a fair and reproducible comparison.

### Dataset selection

To evaluate AutoML frameworks across different classification tasks, we selected datasets spanning binary, multiclass, and multilabel classification problems. These datasets are all real, publicly available OpenML benchmarks, not synthetic toy problems. They vary in size and include a mix of quantitative, qualitative, and mixed feature types. The number of predictive features ranges from just a few to over 200, and the label structures differ in complexity: from simple binary outcomes to multi-class categories and multilabel combinations. For multilabel datasets, we report both the number of original labels and the number of unique label combinations resulting from the Label Powerset transformation. We specifically chose these 21 OpenML datasets because they are among the most commonly referenced benchmarks in the literature, ensuring comparability with prior studies, and they collectively cover a broad spectrum of real-world domains and data characteristics.

To quantify dataset characteristics, we used two complementary metrics. The first, *complexity*, is calculated differently depending on the classification type. For binary and multiclass tasks, complexity^62^ is defined as the ratio of the product of features and classes to samples. For multilabel classification, complexity^11^ incorporates label cardinality (the average number of labels per instance) to compute effective classes. This metric helps contextualize the computational difficulty of each dataset, where higher values suggest a more challenging classification task. We also report the *imbalance*, defined for binary and multiclass tasks as the ratio of the smallest to the largest class size. For multilabel datasets, we report both a global imbalance ratio based on total positive and negative label counts, and a powerset-based ratio computed over unique label combinations. Values near 1 indicate balanced distributions, while values near 0 reflect severe imbalance.

Our selection aimed for broad diversity across several dimensions. We sampled (i) *size tiers*—small ($<1000$ rows), medium (1000–2000), and large ($>2000$); (ii) *application domains*, including finance (*credit-g*), health (*diabetes*, *cardiotocography*), imaging (*wdbc*, *segment*, *scene*), text (*reuters*), and time-series/audio (*birds*); (iii) a wide *complexity spectrum*—from 0.006 for *bank-note-authentication* to 7.766 for *birds*; and (iv) a broad *imbalance range*—from 0.001 for *reuters* to 1.000 for *hill-valley* and *segment*. A complete summary of all datasets is presented in Table 4.

**Table 4:** Summary of the datasets.

Type	ID	Name	Data type	Samples	Features	Classes	Complexity	Imbalance	Classification/prediction task
Binary	31	Credit-g^63^	Mixed	1000	20	2	0.040	0.429	Creditworthiness prediction from personal and financial data
	37	Diabetes^64^	Quantitative	768	8	2	0.021	0.536	Diabetes occurrence based on health and medical measurements like blood pressure and age
	44	Spambase^65^	Quantitative	4601	57	2	0.025	0.650	Email spam based on word/character frequency and statistics like average run length
	1462	Bank-note-authentication^66^	Quantitative	1372	4	2	0.006	0.800	Banknote authenticity based on photograph measurements after a Wavelet transform
	1479	Hill-valley^67^	Quantitative	1212	100	2	0.165	1.000	Valley/hill occurrence based on point coordinates in a two-dimensional graph
	1510	wdbc^68^	Quantitative	569	30	2	0.105	0.594	Breast cancer diagnosis based on cell nuclei characteristics from scanned images
	40945	Titanic^69^	Mixed	1309	13	2	0.020	0.618	Likelihood of passenger survival using their demographic and socioeconomic characteristics
Multiclass	23	Contraceptive-method-choice^70^	Mixed	1473	9	3	0.018	0.529	Contraceptive choice based on demographic and socioeconomic factors like age, education level, and religion
	36	Segment^71^	Mixed	2310	19	7	0.058	1.000	Classify pixels into predefined image regions (e.g., cement, grass, sky, window)
	54	Vehicle^72^	Quantitative	846	18	4	0.085	0.913	Car silhouettes into different types of vehicles based on their geometric features
	181	Yeast^73^	Mixed	1484	8	10	0.054	0.011	Protein localization based on signals of amino acid sequences
	1466	Cardiotocography^74^	Mixed	2126	35	10	0.165	0.091	Fetal morphologic pattern based on cardiotocograph measurements and statistics
	40691	Wine-quality-red^75^	Quantitative	1599	11	6	0.041	0.015	Wine quality based on physicochemical and sensory attributes (e.g. acidity, chrolides, and residual sugar)
	40975	Car^76^	Qualitative	1728	6	4	0.014	0.054	Car acceptability based on door count, maintenance cost, etc
Multilabel	285	Flags^77^	Mixed	194	17	12 (103)	4.336	0.524 (0.043)	Flag element prediction from geographic and visual attributes
	41,464	Birds^78^	Mixed	645	260	19 (133)	7.766	0.056 (0.003)	Presence of multiple bird species in audio recordings
	41,465	Emotions^79^	Mixed	593	72	6 (27)	1.361	0.452 (0.012)	Song emotions based on amplitude, beats per minute, etc
	41,468	Image^80^	Quantitative	2000	135	5 (20)	0.417	0.328 (0.003)	Similar to the “scene” dataset, sharing the same task
	41,470	Reuters^81^	Mixed	2000	243	7 (25)	0.981	0.197 (0.001)	Article categories based on textual features
	41,471	Scene^82^	Quantitative	2407	294	6 (15)	0.787	0.218 (0.003)	Scene/landscape elements based on image data.
	41,473	Yeast^83^	Quantitative	2417	103	14 (198)	2.528	0.434 (0.004)	Yeast gene types via expression patterns

*Qualitative* data represents categories or labels that describe groups, such as gender, colors, or species, commonly used in classification tasks.

*Quantitative* data consists of numerical values that measure quantities, like age, weight, or temperature, often used in regression and classification models.

*Mixed* data combines categorical and numerical features, requiring preprocessing like encoding to ensure compatibility with machine learning algorithms.

In multilabel datasets, values in parentheses indicate metrics computed after applying the label powerset transformation: the *classes* value reflects the number of unique label combinations, and the *imbalance ratio* reflects the class imbalance among them

### Data preprocessing

Minimal preprocessing was applied prior to feeding data into the AutoML tools. Categorical features were integer-encoded using factorization for compatibility across frameworks. Targets were numerically encoded for binary and multiclass tasks; for multilabel classification, labels were either binarized (0 = absence, 1 = presence) or transformed via Label Powerset into unique class identifiers. All other preprocessing, including missing value handling, scaling, and feature engineering, was delegated to the AutoML frameworks to allow them to optimize data preparation internally.

### Tool workflow

Frameworks were selected based on adoption in the research community, open-source availability, and integration with widely used ML libraries to align with standard workflows. Each AutoML framework was executed with minimal intervention, handling data preprocessing, feature selection, model training, and HPO. To ensure fair comparison, key parameters—time budget, evaluation metrics, and parallelization—were unified. Because each framework conducts its own internal hyperparameter search, we gauged consistency by running 20 independent trials per dataset, each started with a different prime-number seed—thereby sampling distinct HPO trajectories without externally altering the tools’ default settings. Table 1 summarizes key features: data handling, classification task coverage, and optimization techniques. Details on dataset partitioning, runtime constraints, and hardware specifications are provided in “Setup” section, while threats to validity are discussed in “Threats to validity” section.

### Performance metrics

To evaluate AutoML frameworks, we define key performance metrics. *Precision* is the proportion of correctly predicted positive instances among all predicted positives for a given class $a$, while *recall* measures the proportion of actual positives correctly identified. Here, $a$ represents a specific class, $TP(a)$ are correctly predicted positives, $FP(a)$ are incorrectly predicted as positive, and $FN(a)$ are actual positives missed by the model. Their formal definitions are given in Eqs. (1) and (2).

1 $$\begin{aligned} Precision(class = a) = \frac{TP(a)}{TP(a) + FP(a)}, \end{aligned}$$

2 $$\begin{aligned} Recall(class = a) = \frac{TP(a)}{TP(a) + FN(a)}. \end{aligned}$$

A more robust and informative metric is the *F*_1_ score, which represents the harmonic mean of precision and recall for a given class $a$. This metric balances the trade-off between precision and recall, making it particularly useful for evaluating imbalanced datasets. However, while the $F_1$ score effectively evaluates individual classes, it does not account for class imbalances across the dataset. To address this, the *weighted* $F_1$ score^84–86^ extends the $F_1$ score by incorporating the *support*, which is the number of true instances (samples) of each class in the dataset. This ensures that the metric reflects the relative importance of each class proportionally. Here, $C$ represents the set of all classes, $n_a$ is the support of class $a$ (i.e., the number of true samples belonging to class $a$), and $F_1~score(a)$ is the $F_1$ score for class $a$. The formulas for both metrics are given in Eqs. (3) and (4).

3 $$\begin{aligned} F_1~score(class = a) = 2 \cdot \frac{\text {Precision}(a) \cdot \text {Recall}(a)}{\text {Precision}(a) + \text {Recall}(a)}, \end{aligned}$$

4 $$\begin{aligned} Weighted~F_1~score(class = a) = \frac{\sum _{a \in C} n_a \cdot F_1~score(a)}{\sum _{a \in C} n_a}. \end{aligned}$$

The calculation of the weighted $F_1$ score varies slightly depending on the type of classification problem:

- Binary: the weighted $F_1$ score is equivalent to the standard $F_1$ score when there are only two classes, as the weights are derived directly from the support of the positive and negative classes.
- Multiclass: the weighted $F_1$ score considers the $F_1~score(a)$ for each class $a$ and weights them by their respective support ($n_a$). This ensures that the contribution of each class to the final score reflects its proportion in the dataset.
- Multilabel: each label is treated as a binary classification problem, and the weighted $F_1$ score is calculated across all labels by summing the weighted $F_1$ scores for each label and dividing by the total support.

The weighted $F_1$ score provides a robust and comprehensive performance measure, making it suitable for various classification tasks with imbalanced datasets. Incorporating the relative importance of each class allows results to be presented and evaluated responsibly and fairly. Each metric ranges from 0 to 1, with values closer to 1 indicating better performance.

Alternative evaluation metrics such as the Area Under the Receiver Operating Characteristic Curve (AUROC), the Area Under the Precision-Recall Curve (AUC-PR), and log-loss were not adopted due to limitations in multiclass and multilabel contexts. AUROC measures the trade-off between true and false positive rates across thresholds, while AUC-PR reflects precision-recall performance for the positive class^87,88^. Both are difficult to interpret or inconsistently defined when label dependencies are present, as in multilabel classification^11^. Logarithmic loss (log-loss) assesses the confidence of probabilistic predictions but is sensitive to class imbalance and lacks intuitive interpretability across tasks^89^. In contrast, the weighted $F_1$ score is robust, interpretable, and applicable across binary, multiclass, and multilabel settings, making it the most suitable choice for unified AutoML benchmarking. Although some frameworks—such as AutoSklearn, FLAML, and mljar-supervised—support interpretability via SHAP (SHapley Additive exPlanations)^90^ and feature importance scores^91^, this study focuses on predictive performance and computational efficiency. A dedicated evaluation of interpretability features is left for future work.

### Result analysis

The evaluation of AutoML frameworks considers both performance and efficiency. To assess model reliability, stability, and computational demands, we analyze the following:

- Weighted $F_1$ score: Evaluates classification performance by balancing precision and recall. We report: (i) *maximum* weighted $F_1$ score, indicating the best-case scenario, (ii) *mean* weighted $F_1$ score, representing expected performance, and (iii) *standard deviation* (SD), measuring variability, where lower values indicate greater stability. Results for binary, multiclass, and multilabel tasks are analyzed separately, with framework rankings aggregated for overall assessment.
- Training time: Assesses efficiency based on execution speed and consistency. We report: (i) *minimum* training time, reflecting the fastest execution, (ii) *mean* training time, estimating typical computational needs, and (iii) *standard deviation*, indicating runtime stability, where lower values suggest more predictable performance.

To ensure robust comparisons, we apply statistical tests in “Statistical analysis” section to evaluate performance ($F_1$ score) and efficiency (training time) across frameworks. The analysis considers three scenarios: per-dataset, across-datasets, and all-datasets. Per-dataset analysis examines each dataset individually, across-datasets analysis ranks frameworks based on aggregated results, and all-datasets analysis identifies the best-performing framework overall. These tests confirm that observed differences are statistically meaningful rather than random variations. Additional methodological details are also provided in the section.

## Experiments

### Setup

The experimental setup involved implementing the experiments in Python and consolidating the code and instructions for reproducibility in the following public GitHub repository: https://github.com/marcelovca90/auto-ml-evaluation. Key functionalities, including data retrieval, sampling, and target encoding, were executed using the Pandas^92^, scikit-learn^21^, and scikit-multilearn^93^ (for label powerset) libraries. Datasets were sourced from the public API of the OpenML tool^94^.

To ensure robust and random evaluations, each dataset underwent 20 rounds of shuffling using prime number seeds (2–71) for the random number generator (RNG). This shuffling mitigates bias and strengthens the generalizability of the findings. The data was split into an 80/20 ratio for training and testing in each round, ensuring consistency across evaluations. Additionally, the prime number seeds were used to initialize the random state of the AutoML frameworks, enhancing reproducibility in their search processes. Prime numbers were chosen as seeds to minimize unintended correlations in dataset shuffling. Unlike composite numbers, they reduce the risk of systematic bias and improve statistical randomness. This approach aligns with best practices in cryptography and randomized algorithms, reinforcing the robustness and fairness of experimental outcomes^95^.

For each seed, a shell script orchestrated the experiments in a dedicated Linux environment, executing all AutoML frameworks per dataset with a fixed 5-min timeout. This limit ensured fair comparison across frameworks with varying optimization speeds—some rely on fast heuristics, others on longer runtimes. A fixed cap prevents bias toward either. RAM was monitored throughout, but no framework exceeded system memory or failed due to resource limits, so no cap was enforced. After each iteration, results were aggregated using NumPy^96^ to compute basic statistics (max, min, mean, and standard deviation), providing insight into framework performance.

The experiments were conducted on a computer with an AMD® Ryzen™ 9 5900X processor, 128 GB of DDR4-3200 RAM, Nvidia GeForce® RTX™ 3090 24 GB GDDR6X dedicated graphics card (driver version 546.17), and Ubuntu operating system (version 22.04.2 LTS) under Windows 11 Professional (version 22H2) using WSL (Windows Subsystem for Linux).

For a more comprehensive understanding of the experiment’s configuration, Table 5 details the custom parameters used for each framework. This table provides insight into the specific settings employed during the construction, fitting, and prediction processes through custom parameters.

### Results and discussion

This section presents experimental results for binary, multiclass, and multilabel classification tasks. The primary performance metric is the weighted $F_1$ score (defined in “Performance metrics” section), which balances precision and recall while accounting for class imbalance. Training time is also analyzed to assess computational efficiency.

Each framework-dataset pair was evaluated over 20 runs with shuffled splits using prime number seeds. We report the maximum, mean, and standard deviation of the weighted $F_1$ score. A high maximum indicates strong predictive capacity in at least one trial, while a high mean and low standard deviation reflect robustness to hyperparameter and data variation.

While prediction time matters in real-time settings such as Network Traffic Analysis (NTA)^97,98^, we focus on training time, as excessive durations can hinder model usability.

For each classification type, results are summarized in tables and figures reporting weighted $F_1$ scores and training times. The discussion is structured around the following key aspects:

- *Performance analysis* Comparison of frameworks based on weighted $F_1$ scores across datasets of varying complexity. The analysis identifies where advanced techniques like ensemble learning or NAS contribute to higher accuracy.
- *Training time analysis* Examination of computational efficiency through training time comparisons. This highlights the trade-off between frameworks that maximize accuracy through exhaustive optimization versus those prioritizing speed through adaptive resource allocation.
- *Usability and scalability comparison* Evaluation of framework behavior under growing data complexity and resource constraints, including how they manage missing values, categorical features, and class imbalance. We highlight differences in scalability and design focus (e.g., exhaustive search vs. adaptive strategies).
- *Biases, limitations, and failure modes* We log crashes, timeouts, and degenerate predictions (e.g., NaNs, single-class outputs), tracing them to issues such as inadequate handling of missing values, categorical encoding, or label imbalance. These cases expose framework brittleness and complement quantitative results with robustness insights.
- *Insights on complexity and imbalance* Expanded analysis of the relationship between dataset complexity, class imbalance, and framework performance, with emphasis on cases where traditional complexity metrics fail to explain results. In both standard and multilabel settings, severe imbalance—whether across labels or label combinations—often proved more predictive of failure than complexity alone, highlighting the need for imbalance-aware modeling.
- *Conclusion* Summary of trade-offs between frameworks favoring exhaustive search and those optimized for efficiency.

Next, we present and discuss the results for each classification task, followed by overall findings and insights. Finally, we examine threats to validity, considering potential limitations and the reliability of our conclusions.

**Table 5:** Custom settings used in each framework.

Framework	Classifier constructor		Fit/predict methods	
	Argument	Value	Argument	Value
AutoGluon	eval_metric	“f1_weighted”	time_limit	EXEC_TIME_SECONDS
	Label	“class”	num_cpus	NUM_CPUS
AutoKeras	multi_label	True/false	Epochs	300
	max_trials	3		
	Overwrite	True		
	Seed	SEED		
Auto-PyTorch	n_jobs	NUM_CPUS	optimize_metric	“f1_weighted”
	Seed	SEED	budget_type	“runtime”
			total_walltime_limit	EXEC_TIME_SECONDS
			func_eval_time_limit_secs	EXEC_TIME_SECONDS//10
AutoSklearn	time_left_for_this_task	EXEC_TIME_SECONDS	–	–
	per_run_time_limit	EXEC_TIME_SECONDS//10		
	resampling_strategy	“cv”		
	resampling_strategy_args	{“folds”: 5}		
	n_jobs	NUM_CPUS		
	seed	SEED		
EvalML	problem_type	“binary” / multiclass”	–	–
	max_time	EXEC_TIME_SECONDS		
	n_jobs	NUM_CPUS		
	random_seed	SEED		
FEDOT	problem	“classification”	–	–
	timeout	EXEC_TIME_MINUTES		
	seed	SEED		
FLAML	metric	“accuracy”	–	–
	task	“classification”		
	time_budget	EXEC_TIME_SECONDS		
GAMA	max_total_time	EXEC_TIME_SECONDS	–	–
	max_eval_time	EXEC_TIME_SECONDS//10		
	store	“nothing”		
	n_jobs	NUM_CPUS		
	random_seed	SEED		
H2O	max_runtime_secs	EXEC_TIME_SECONDS	–	–
	sort_metric	“auto”		
	nfolds	5		
	Seed	SEED		
LightAutoML	task	Task(“binary” / “multiclass”, “metric”=“accuracy”)	roles	{“target”: “class”}
	Timeout	EXEC_TIME_SECONDS		
	cpu_limit	NUM_CPUS		
	reader_params	{“cv”: 5, “n_jobs”: NUM_CPUS, “random_state”: SEED}		
Lightwood	problem_definition	ProblemDefinition.from_dict({“target”: “class”, “time_aim”: EXEC_TIME_SECONDS, “seed_nr”: SEED, “strict_mode”: False})	–	–
mljar-supervised	total_time_limit	EXEC_TIME_SECONDS	–	–
	ml_task	“binary_classification” / “multiclass_classification”		
	random_state	SEED		
NaiveAutoML	Timeout	EXEC_TIME_SECONDS	–	–
	execution_timeout	EXEC_TIME_SECONDS//10		
	task_type	“classification”		
	Scoring	“f1”		
PyCaret	Target	“class”	budget_time	EXEC_TIME_SECONDS
	session_id	SEED	n_select	1
	folds	5	sort	“Accuracy”
	n_jobs	NUM_CPUS		
TPOT	max_time_mins	EXEC_TIME_MINUTES	–	–
	max_eval_time_mins	EXEC_TIME_MINUTES//10		
	cv	5		
	n_jobs	NUM_CPUS		
	random_state	SEED		

EXEC_TIME_SECONDS is a constant denoting the execution time in seconds (e.g., 300). When present, the symbol “//” means integer division.

NUM_CPUS is a constant denoting the total number of virtual processors in the setup (e.g., 24).

SEED is the current prime number used as seed by the RNG; it can be one of [2, 3, 5, 7, 11, 13, 17, 19, 23, 29, 31, 37, 41, 43, 47, 53, 59, 61, 67, 71].

EXEC_TIME_MINUTES is a constant denoting the execution time in minutes (e.g., 5)

#### Binary scenario

**Fig. 6:**
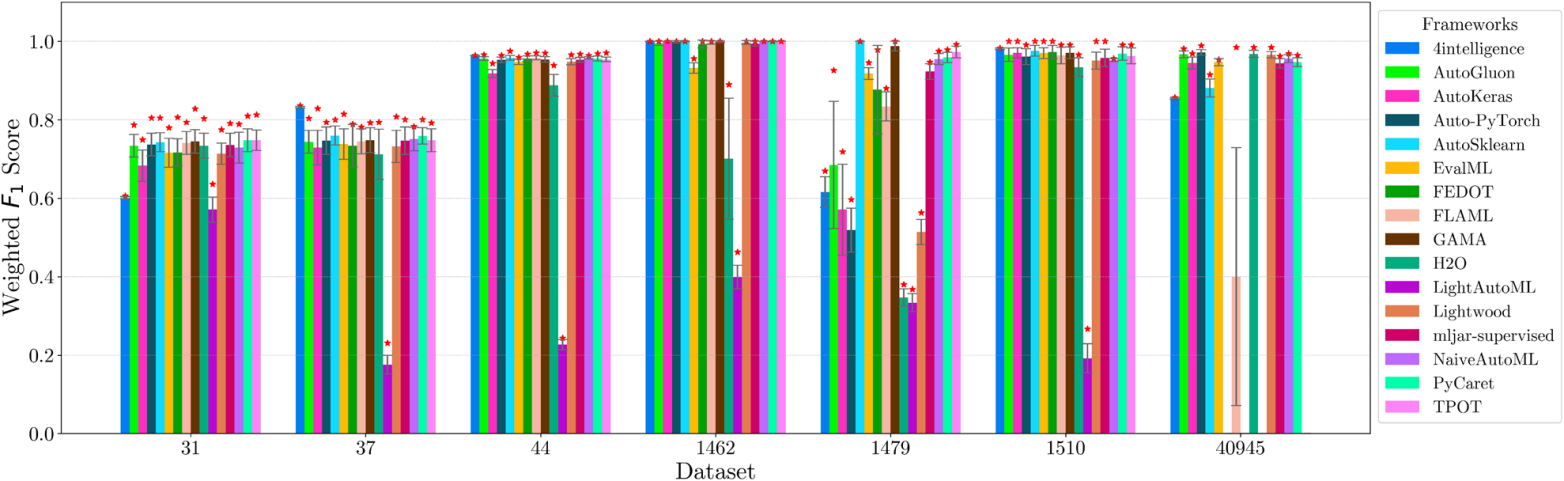
Binary weighted $F_1$ score—maximum (mean ± std dev).

**Fig. 7:**
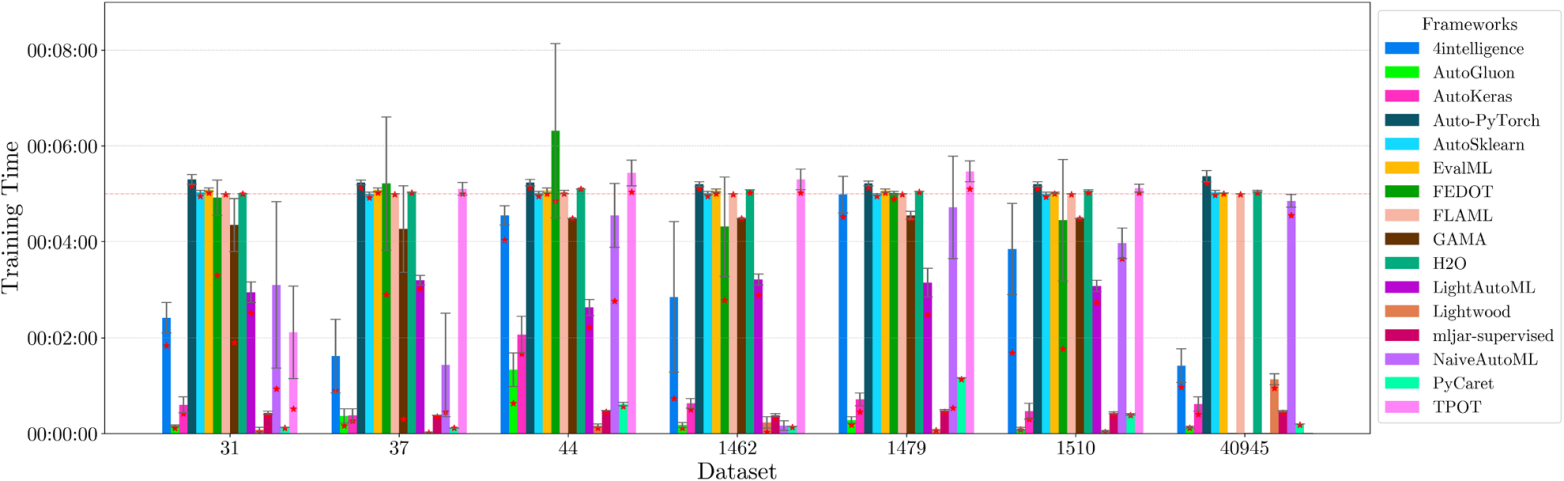
Binary training time—minimum (mean ± std dev).

From here on, each dataset is identified as “*Name* (ID, *C*/*I*)”, where *C* denotes complexity and *I* indicates class imbalance, as per Table 4; scores are reported as “max. (mean ± SD)” for $F_{1}$, and training times as “min. (mean ± SD).” For the binary datasets, Tables 6a and b, along with Figs. 6 and 7, summarize each framework’s weighted $F_{1}$ score and training time.

**Table 6:** Performance summary for binary classification tasks.

(a) Binary weighted $F_1$ Score—maximum (mean ± std dev)							
Framework	Dataset						
	31	37	44	1462	1479	1510	40945
4intelligence	0.606 (0.601 ± 0.003)	0.836 (0.832 ± 0.002)	0.964 (0.964 ± 0.000)	1.000 (1.000 ± 0.000)	0.670 (0.616 ± 0.039)	0.983 (0.981 ± 0.002)	0.857 (0.855 ± 0.002)
AutoGluon	0.787 (0.734 ± 0.029)	0.804 (0.744 ± 0.029)	0.966 (0.956 ± 0.005)	1.000 (0.995 ± 0.005)	0.926 (0.685 ± 0.162)	1.000 (0.966 ± 0.017)	0.981 (0.967 ± 0.009)
AutoKeras	0.749 (0.683 ± 0.040)	0.829 (0.729 ± 0.044)	0.944 (0.918 ± 0.010)	1.000 (1.000 ± 0.000)	0.719 (0.571 ± 0.116)	1.000 (0.971 ± 0.013)	0.969 (0.945 ± 0.015)
Auto-PyTorch	0.805 (0.737 ± 0.029)	0.794 (0.747 ± 0.035)	0.964 (0.953 ± 0.007)	1.000 (0.999 ± 0.003)	0.597 (0.519 ± 0.056)	0.991 (0.961 ± 0.020)	0.989 (0.972 ± 0.007)
AutoSklearn	0.805 (0.743 ± 0.024)	0.800 (0.760 ± 0.024)	0.975 (0.958 ± 0.006)	1.000 (1.000 ± 0.001)	1.000 (1.000 ± 0.000)	1.000 (0.976 ± 0.014)	0.915 (0.881 ± 0.023)
EvalML	0.780 (0.716 ± 0.037)	0.815 (0.738 ± 0.039)	0.959 (0.948 ± 0.007)	0.956 (0.932 ± 0.013)	0.946 (0.918 ± 0.015)	1.000 (0.970 ± 0.014)	0.954 (0.947 ± 0.009)
FEDOT	0.806 (0.717 ± 0.035)	0.789 (0.734 ± 0.054)	0.968 (0.956 ± 0.007)	1.000 (0.993 ± 0.010)	0.979 (0.877 ± 0.113)	1.000 (0.973 ± 0.017)	–
FLAML	0.794 (0.741 ± 0.029)	0.782 (0.745 ± 0.032)	0.971 (0.958 ± 0.005)	1.000 (0.997 ± 0.004)	0.880 (0.834 ± 0.037)	0.991 (0.964 ± 0.021)	0.985 (0.400 ± 0.329)
GAMA	0.828 (0.745 ± 0.030)	0.793 (0.748 ± 0.032)	0.970 (0.954 ± 0.007)	1.000 (0.999 ± 0.002)	1.000 (0.988 ± 0.012)	0.991 (0.971 ± 0.015)	–
H2O	0.803 (0.734 ± 0.032)	0.794 (0.712 ± 0.064)	0.939 (0.888 ± 0.028)	0.890 (0.701 ± 0.154)	0.380 (0.347 ± 0.022)	0.966 (0.934 ± 0.024)	0.985 (0.968 ± 0.009)
LightAutoML	0.636 (0.571 ± 0.032)	0.231 (0.176 ± 0.024)	0.244 (0.227 ± 0.013)	0.463 (0.399 ± 0.030)	0.368 (0.334 ± 0.023)	0.267 (0.192 ± 0.037)	–
Lightwood	0.775 (0.714 ± 0.027)	0.808 (0.732 ± 0.041)	0.966 (0.948 ± 0.008)	1.000 (0.997 ± 0.005)	0.563 (0.514 ± 0.032)	1.000 (0.951 ± 0.022)	0.985 (0.966 ± 0.008)
mljar-supervised	0.791 (0.736 ± 0.030)	0.801 (0.747 ± 0.035)	0.968 (0.953 ± 0.006)	1.000 (0.994 ± 0.006)	0.947 (0.923 ± 0.020)	1.000 (0.958 ± 0.022)	0.962 (0.944 ± 0.012)
NaiveAutoML	0.789 (0.729 ± 0.039)	0.783 (0.751 ± 0.030)	0.964 (0.958 ± 0.003)	1.000 (1.000 ± 0.000)	0.975 (0.955 ± 0.014)	0.956 (0.952 ± 0.004)	0.969 (0.956 ± 0.010)
PyCaret	0.810 (0.748 ± 0.029)	0.801 (0.759 ± 0.021)	0.969 (0.956 ± 0.006)	1.000 (1.000 ± 0.001)	0.979 (0.959 ± 0.013)	0.991 (0.969 ± 0.017)	0.965 (0.947 ± 0.011)
TPOT	0.813 (0.748 ± 0.026)	0.792 (0.748 ± 0.029)	0.971 (0.954 ± 0.006)	1.000 (1.000 ± 0.000)	0.992 (0.973 ± 0.015)	0.991 (0.963 ± 0.020)	–

(b) Binary training times—minimum (mean ± std dev)							
Framework	Dataset						
	31	37	44	1462	1479	1510	40945
4intelligence	01:50 (02:25 ± 00:19)	00:54 (01:37 ± 00:46)	04:02 (04:33 ± 00:12)	00:44 (02:51 ± 01:34)	04:31 (04:59 ± 00:23)	01:41 (03:51 ± 00:57)	00:58 (01:25 ± 00:21)
AutoGluon	00:07 (00:10 ± 00:01)	00:10 (00:22 ± 00:09)	00:38 (01:20 ± 00:21)	00:07 (00:11 ± 00:03)	00:11 (00:17 ± 00:04)	00:05 (00:06 ± 00:02)	00:07 (00:08 ± 00:01)
AutoKeras	00:25 (00:36 ± 00:10)	00:16 (00:23 ± 00:08)	01:40 (02:04 ± 00:23)	00:30 (00:38 ± 00:06)	00:27 (00:43 ± 00:08)	00:18 (00:28 ± 00.10)	00:24 (00:37 ± 00:09)
Auto-PyTorch	05:10 (05:18 ± 00:06)	05:07 (05:14 ± 00:03)	05:07 (05:14 ± 00:04)	05:06 (05:12 ± 00:03)	05:07 (05:13 ± 00:03)	05:06 (05:12 ± 00:03)	05:13 (05:22 ± 00:07)
AutoSklearn	04:57 (05:02 ± 00:02)	04:55 (05:00 ± 00:02)	04:57 (05:01 ± 00:02)	04:57 (05:01 ± 00:02)	04:57 (04:59 ± 00:01)	04:56 (05:00 ± 00:02)	04:58 (05:02 ± 00:02)
EvalML	05:01 (05:05 ± 00:02)	05:01 (05:05 ± 00:02)	05:00 (05:04 ± 00:03)	05:00 (05:04 ± 00:02)	05:01 (05:04 ± 00:02)	05:00 (05:01 ± 00:01)	05:00 (05:01 ± 00:00)
FEDOT	03:18 (04:55 ± 00:22)	02:54 (05:13 ± 01:23)	04:51 (06:19 ± 01:49)	02:47 (04:19 ± 01:02)	04:53 (05:01 ± 00:02)	01:46 (04:27 ± 01:16)	–
FLAML	04:59 (05:00 ± 00:00)	04:59 (05:00 ± 00:00)	05:00 (05:02 ± 00:02)	04:59 (05:00 ± 00:00)	04:59 (05:00 ± 00:00)	04:59 (05:00 ± 00:00)	04:59 (05:00 ± 00:00)
GAMA	01:54 (04:21 ± 00:33)	00:18 (04:16 ± 00:54)	04:29 (04:29 ± 00:00)	04:29 (04:29 ± 00:00)	04:29 (04:33 ± 00:05)	04:29 (04:29 ± 00:00)	–
H2O	05:00 (05:00 ± 00:00)	05:01 (05:02 ± 00:00)	05:06 (05:06 ± 00:00)	05:01 (05:05 ± 00:00)	05:02 (05:03 ± 00:00)	05:01 (05:04 ± 00:01)	05:01 (05:03 ± 00:01)
LightAutoML	02:31 (02:57 ± 00:13)	03:02 (03:12 ± 00:06)	02:13 (02:38 ± 00:10)	02:53 (03:13 ± 00:07)	02:29 (03:09 ± 00:18)	02:44 (03:05 ± 00:07)	–
Lightwood	00:02 (00:05 ± 00:03)	00:01 (00:02 ± 00:00)	00:07 (00:10 ± 00:02)	00:02 (00:14 ± 00:07)	00:04 (00:05 ± 00:00)	00:02 (00:03 ± 00:01)	00:57 (01:08 ± 00:07)
mljar-supervised	00:23 (00:26 ± 00:02)	00:21 (00:23 ± 00:00)	00:27 (00:29 ± 00:00)	00:20 (00:23 ± 00:02)	00:27 (00:29 ± 00:01)	00:24 (00:26 ± 00:01)	00:26 (00:28 ± 00:01)
NaiveAutoML	00:56 (03:06 ± 01:44)	00:27 (01:26 ± 01:05)	02:46 (04:33 ± 00:40)	00:00 (00:10 ± 00:06)	00:32 (04:43 ± 01:04)	03:39 (03:58 ± 00:19)	04:33 (04:51 ± 00:08)
PyCaret	00:07 (00:08 ± 00:00)	00:07 (00:07 ± 00:00)	00:34 (00:37 ± 00:02)	00:08 (00:09 ± 00:00)	01:08 (01:10 ± 00:00)	00:23 (00:24 ± 00:01)	00:11 (00:12 ± 00:00)
TPOT	00:31 (02:07 ± 00:58)	05:00 (05:06 ± 00:08)	05:02 (05:26 ± 00:16)	05:01 (05:18 ± 00:13)	05:06 (05:28 ± 00:13)	05:01 (05:07 ± 00:05)	–

##### Performance analysis

From a performance perspective, most frameworks achieved strong results on simpler datasets, as shown in Table 6a and Fig. 6—particularly *bank-note-authentication* (ID 1462, 0.006/0.800), where perfect $F_{1}$ scores were common. However, as dataset complexity increased, performance variability became more pronounced. On *hill-valley* (ID 1479, 0.165/1.000), frameworks exhibited a wide range of results, with AutoSklearn and GAMA achieving a perfect score, followed by TPOT with 0.992. In contrast, AutoGluon, despite a maximum of 0.926 (mean: 0.685 ± 0.162), showed better adaptability to training time, whereas LightAutoML struggled, reaching only 0.368. These differences highlight the impact of framework design on handling complex datasets. Frameworks like AutoSklearn and Auto-PyTorch use advanced Bayesian HPO^38^, while GAMA and TPOT employ genetic programming^49^, both consistently yielding high scores and robust performance. Meanwhile, AutoGluon leverages stacked ensembles and adaptive model selection^35^, maintaining competitive results with lower training time. In contrast, LightAutoML and Lightwood, with limited feature engineering and less aggressive HPO^44,45^, showed performance degradation on high-dimensional datasets.

##### Training time analysis

Training time analysis revealed a clear trade-off between exhaustive optimization and rapid execution. As shown in Table 6b and Fig. 7, heavy-search frameworks such as AutoSklearn and TPOT consistently used nearly the full 5-min budget (05:00 ± 00:02), achieving the highest $F_{1}$ scores. In contrast, adaptive pipelines dynamically adjusted their runtimes: AutoGluon completed *bank-note-authentication* (ID 1462, 0.006/0.800) in just 00:11 ± 00:03 while still reaching $F_{1}=1.000$, and on *hill-valley* (ID 1479, 0.165/1.000) it finished in 00:17 ± 00:04 (with $F_{1}=0.926$). “Speed-first” tools ran dramatically faster but at lower accuracy – for example, Lightwood ran in about 2 s ($\approx 150\times$ faster) with $F_{1}=0.563$, and LightAutoML ran in 02:29 ± 00:18 ($\approx 2\times$ faster) with $F_{1}=0.368$. These results underscore how runtime strategies directly affect both speed and accuracy.

Usability and scalability comparison

On *spambase* (ID 44, 0.025/0.650), frameworks that relied on extensive HPO required the full training budget to achieve high scores, while others produced comparable results in significantly less time. This supports findings that HPO through Bayesian optimization^38^ or genetic programming^49^ can increase training time without necessarily yielding substantial performance improvements. On *hill-valley* (ID 1479, 0.165/1.000), which contains 100 features, scalability differences were more apparent. Some frameworks required the full training budget due to their reliance on exhaustive model selection^38^, whereas others completed training in a fraction of the time at the cost of lower $F_{1}$, illustrating the speed–accuracy trade-off in high-dimensional tasks^35,44^.

##### Biases, limitations, and failure modes

Four frameworks—FEDOT, GAMA, LightAutoML, and TPOT—crashed on the moderately imbalanced *titanic* set (ID 40945, 0.020/0.618) for distinct but related reasons: FEDOT stalled on missing-value imputation, TPOT failed to encode non-numeric categoricals, and GAMA and LightAutoML aborted when extreme class imbalance left the target effectively single-class (the latter performing a sanity check). AutoSklearn and AutoGluon completed the task, evidencing resilient pipelines with built-in imputation, encoding, and class-balancing heuristics. Two general patterns emerged across the seven binary datasets: (i) exhaustive Bayesian or genetic search engines (AutoSklearn, Auto-PyTorch, TPOT) achieved the highest $F_{1}$ scores but often overfit the trivially separable *bank-note-authentication* (ID 1462, 0.006/0.800); (ii) speed-first pipelines (LightAutoML, Lightwood) ran dramatically faster but suffered major accuracy losses ($\ge 0.6$ in $F_{1}$) on noisy, high-dimensional *hill-valley* (ID 1479, 0.165/1.000). Tree-ensemble tools (AutoGluon, FLAML) showed only mild dips on imbalanced sets, likely because their 5-minute budgets precluded advanced re-sampling or cost-sensitive learning.

##### Insights on complexity and imbalance

Dataset difficulty hinges on how complexity interacts with class skew. The noisy, high-dimensional *hill-valley* (ID 1479, 0.165/1.000) defeated every speed-first tool despite moderate complexity, whereas the separable yet skewed *bank-note-authentication* (ID 1462, 0.006/0.800) still yielded perfect $F_{1}$ for most frameworks. Low complexity but moderate skew in *credit-g* (ID 31, 0.040/0.429) caused dips in tree ensembles, while slight skew in *diabetes* (ID 37, 0.021/0.536) challenged pipelines without feature-interaction search. High complexity and moderate skew in *wdbc* (ID 1510, 0.105/0.594) saw uniformly strong scores, confirming that informative feature geometry can override imbalance. Together, these findings show that neither complexity nor skew alone predicts risk; it is their joint profile that decides success.

##### Conclusion

The binary scenario analysis reveals trade-offs among the evaluated frameworks. AutoSklearn and TPOT, which employ comprehensive model search and HPO^38,49^, achieve high accuracy but require longer training times, prioritizing performance over efficiency. AutoGluon and FLAML balance accuracy and scalability through efficient model selection and automated ensembling^35,41^. In contrast, LightAutoML and GAMA, which limit computational resources^42,44^, exhibit higher performance variability, particularly on complex datasets, due to constraints in feature engineering and optimization. These results highlight the need for adaptable frameworks with low variability to ensure reliable performance across diverse datasets.

#### Multiclass scenario

**Fig. 8:**
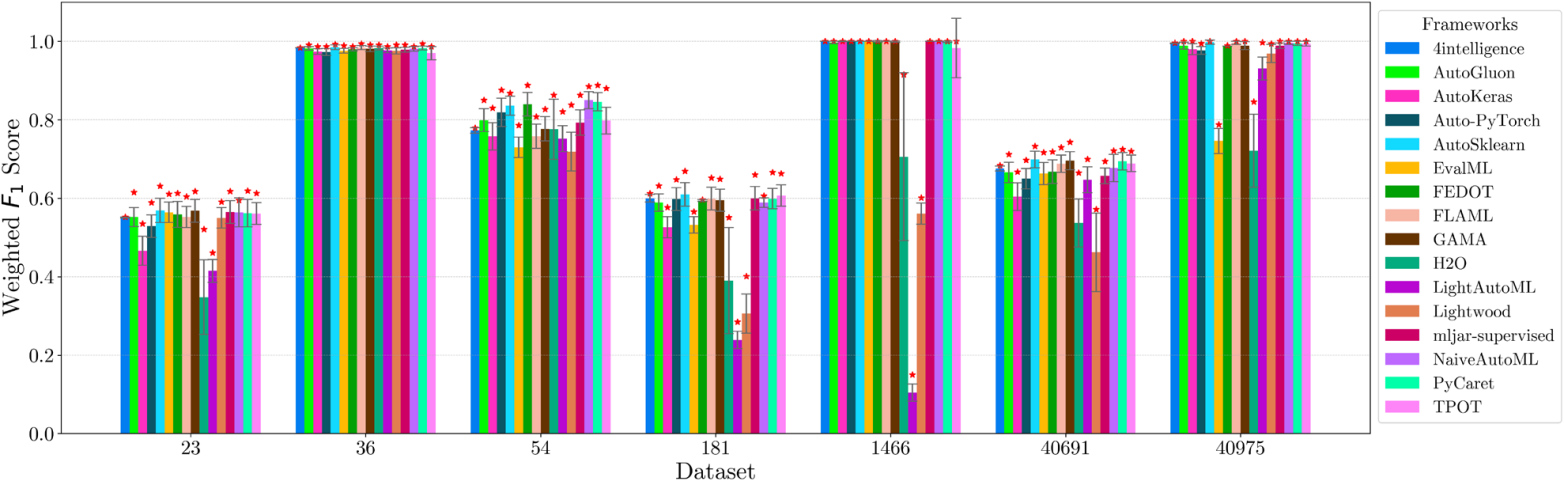
Multiclass weighted $F_1$ score—maximum (mean ± std dev).

**Fig. 9:**
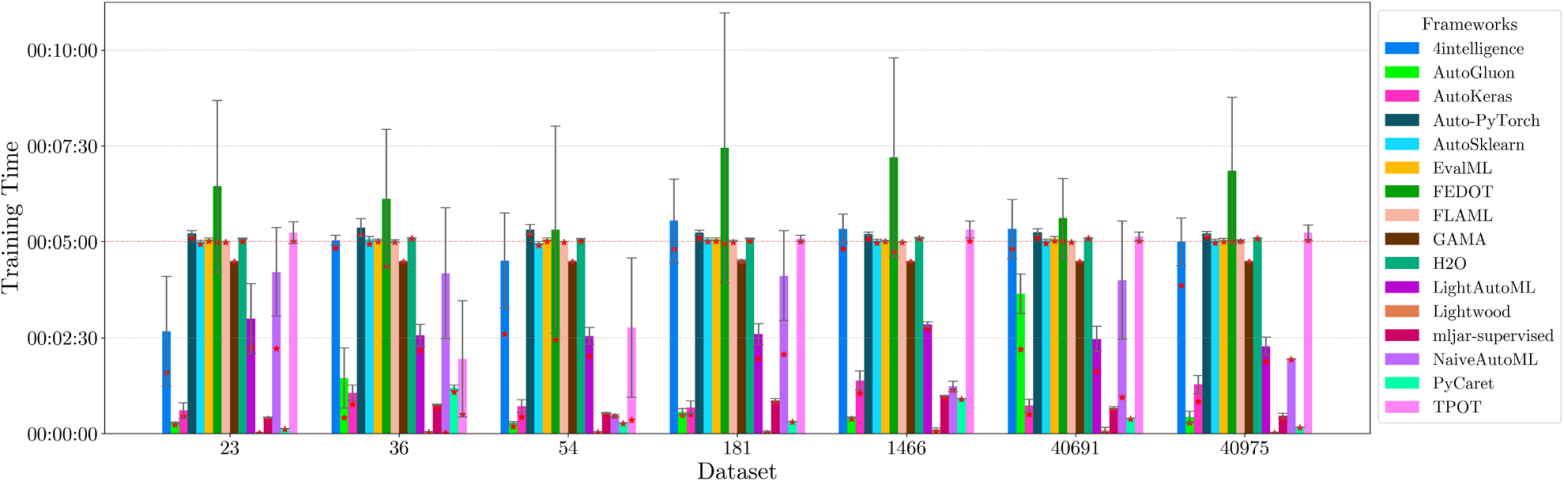
Multiclass training time—minimum (mean ± Std Dev).

Departing from the binary datasets, analyzing the multiclass scenarios through Tables 7a and b, as well as Figs. 8 and 9, we observe significant variation in performance and training times across frameworks.

**Table 7:** Performance summary for multiclass classification tasks.

(a) Multiclass weighted F1 score—maximum (mean ± std dev).							
Framework	Dataset						
	23	36	54	181	1466	40691	40975
4intelligence	0.552 (0.551 ± 0.001)	0.984 (0.984 ± 0.000)	0.779 (0.773 ± 0.007)	0.612 (0.600 ± 0.010)	1.000 (1.000 ± 0.000)	0.684 (0.676 ± 0.006)	0.996 (0.995 ± 0.001)
AutoGluon	0.615 (0.552 ± 0.024)	0.991 (0.982 ± 0.005)	0.850 (0.799 ± 0.029)	0.632 (0.589 ± 0.022)	1.000 (0.997 ± 0.003)	0.712 (0.666 ± 0.026)	1.000 (0.989 ± 0.009)
AutoKeras	0.535 (0.466 ± 0.037)	0.987 (0.974 ± 0.007)	0.830 (0.758 ± 0.035)	0.577 (0.526 ± 0.027)	1.000 (1.000 ± 0.001)	0.667 (0.604 ± 0.035)	1.000 (0.980 ± 0.014)
Auto-PyTorch	0.589 (0.529 ± 0.029)	0.987 (0.973 ± 0.009)	0.876 (0.819 ± 0.036)	0.648 (0.598 ± 0.029)	1.000 (1.000 ± 0.000)	0.697 (0.650 ± 0.026)	0.995 (0.977 ± 0.010)
AutoSklearn	0.631 (0.569 ± 0.031)	0.993 (0.985 ± 0.006)	0.868 (0.836 ± 0.024)	0.669 (0.610 ± 0.030)	1.000 (1.000 ± 0.000)	0.733 (0.699 ± 0.021)	1.000 (0.998 ± 0.005)
EvalML	0.611 (0.564 ± 0.026)	0.989 (0.976 ± 0.006)	0.786 (0.730 ± 0.026)	0.566 (0.532 ± 0.021)	1.000 (1.000 ± 0.000)	0.717 (0.663 ± 0.028)	0.788 (0.746 ± 0.032)
FEDOT	0.613 (0.559 ± 0.033)	0.987 (0.980 ± 0.004)	0.888 (0.840 ± 0.030)	0.596 (0.594 ± 0.002)	1.000 (1.000 ± 0.002)	0.719 (0.668 ± 0.030)	0.989 (0.988 ± 0.000)
FLAML	0.604 (0.552 ± 0.027)	0.994 (0.984 ± 0.005)	0.808 (0.758 ± 0.031)	0.652 (0.599 ± 0.029)	1.000 (1.000 ± 0.000)	0.730 (0.688 ± 0.022)	1.000 (0.996 ± 0.004)
GAMA	0.618 (0.568 ± 0.029)	0.991 (0.981 ± 0.007)	0.827 (0.777 ± 0.031)	0.649 (0.595 ± 0.028)	1.000 (0.999 ± 0.002)	0.743 (0.696 ± 0.023)	1.000 (0.989 ± 0.010)
H2O	0.521 (0.348 ± 0.095)	0.991 (0.983 ± 0.005)	0.863 (0.776 ± 0.077)	0.551 (0.390 ± 0.135)	0.915 (0.706 ± 0.214)	0.665 (0.537 ± 0.061)	0.846 (0.721 ± 0.093)
LightAutoML	0.461 (0.415 ± 0.029)	0.987 (0.977 ± 0.006)	0.821 (0.752 ± 0.033)	0.285 (0.239 ± 0.022)	0.150 (0.104 ± 0.022)	0.700 (0.647 ± 0.033)	0.997 (0.931 ± 0.029)
Lightwood	0.591 (0.550 ± 0.026)	0.991 (0.976 ± 0.008)	0.838 (0.719 ± 0.049)	0.401 (0.306 ± 0.050)	0.601 (0.561 ± 0.027)	0.572 (0.462 ± 0.100)	0.994 (0.969 ± 0.023)
mljar-supervised	0.618 (0.565 ± 0.029)	0.991 (0.979 ± 0.006)	0.863 (0.793 ± 0.032)	0.660 (0.600 ± 0.030)	1.000 (1.000 ± 0.000)	0.694 (0.657 ± 0.020)	1.000 (0.989 ± 0.007)
NaiveAutoML	0.595 (0.564 ± 0.037)	0.987 (0.981 ± 0.006)	0.885 (0.850 ± 0.022)	0.606 (0.589 ± 0.012)	1.000 (1.000 ± 0.000)	0.721 (0.677 ± 0.035)	1.000 (0.996 ± 0.004)
PyCaret	0.619 (0.562 ± 0.035)	0.994 (0.983 ± 0.005)	0.888 (0.846 ± 0.023)	0.666 (0.599 ± 0.026)	1.000 (0.999 ± 0.002)	0.725 (0.694 ± 0.022)	1.000 (0.995 ± 0.005)
TPOT	0.613 (0.561 ± 0.028)	0.987 (0.970 ± 0.017)	0.880 (0.798 ± 0.034)	0.663 (0.607 ± 0.027)	1.000 (0.983 ± 0.076)	0.720 (0.689 ± 0.021)	1.000 (0.994 ± 0.006)

(b) Multiclass training time—minimum (mean ± std dev).							
Framework	Dataset						
	23	36	54	181	1466	40691	40975
4intelligence	01:36 (02:40 ± 01:26)	04:50 (05:02 ± 00:08)	02:36 (04:31 ± 01:14)	04:49 (05:33 ± 01:05)	04:49 (05:20 ± 00:23)	04:49 (05:20 ± 00:46)	03:52 (05:00 ± 00:37)
AutoGluon	00:14 (00:17 ± 00:01)	00:25 (01:27 ± 00:47)	00:11 (00:17 ± 00:02)	00:29 (00:34 ± 00:05)	00:23 (00:25 ± 00:01)	02:12 (03:39 ± 00:31)	00:17 (00:26 ± 00:09)
AutoKeras	00:27 (00:36 ± 00:12)	00:46 (01:04 ± 00:12)	00:26 (00:43 ± 00:10)	00:30 (00:41 ± 00:10)	01:03 (01:23 ± 00:15)	00:30 (00:44 ± 00:10)	00:50 (01:17 ± 00:14)
Auto-PyTorch	05:06 (05:13 ± 00:04)	05:11 (05:22 ± 00:14)	05:12 (05:19 ± 00:08)	05:05 (05:14 ± 00:04)	05:04 (05:12 ± 00:03)	05:06 (05:15 ± 00:05)	05:07 (05:13 ± 00:03)
AutoSklearn	04:56 (05:00 ± 00:02)	04:56 (05:04 ± 00:04)	04:54 (04:58 ± 00:02)	05:00 (05:04 ± 00:02)	04:58 (05:02 ± 00:02)	04:57 (05:02 ± 00:02)	04:58 (05:02 ± 00:02)
EvalML	05:01 (05:04 ± 00:02)	05:00 (05:02 ± 00:02)	05:00 (05:04 ± 00:02)	05:01 (05:04 ± 00:02)	05:00 (05:01 ± 00:01)	05:01 (05:05 ± 00:03)	05:00 (05:03 ± 00:02)
FEDOT	04:59 (06:27 ± 02:14)	04:22 (06:07 ± 01:49)	02:27 (05:19 ± 02:42)	04:57 (07:27 ± 03:31)	04:45 (07:12 ± 02:36)	05:01 (05:37 ± 01:02)	05:00 (06:51 ± 01:55)
FLAML	04:59 (05:00 ± 00:00)	04:59 (05:01 ± 00:02)	04:59 (05:00 ± 00:00)	04:59 (05:01 ± 00:01)	04:59 (04:59 ± 00:00)	04:59 (05:00 ± 00:00)	05:00 (05:01 ± 00:02)
GAMA	04:29 (04:29 ± 00:00)	04:29 (04:29 ± 00:00)	04:29 (04:29 ± 00:00)	04:29 (04:30 ± 00:02)	04:29 (04:29 ± 00:00)	04:29 (04:29 ± 00:00)	04:29 (04:29 ± 00:00)
H2O	05:00 (05:05 ± 00:01)	05:05 (05:06 ± 00:00)	05:01 (05:05 ± 00:00)	05:01 (05:05 ± 00:01)	05:05 (05:06 ± 00:01)	05:05 (05:06 ± 00:00)	05:05 (05:06 ± 00:00)
LightAutoML	02:15 (03:00 ± 00:55)	02:10 (02:34 ± 00:17)	02:01 (02:33 ± 00:13)	01:57 (02:36 ± 00:16)	02:44 (02:51 ± 00:04)	01:37 (02:28 ± 00:20)	01:53 (02:17 ± 00:14)
Lightwood	00:01 (00:01 ± 00:00)	00:02 (00:03 ± 00:00)	00:02 (00:03 ± 00:00)	00:01 (00:02 ± 00:02)	00:03 (00:07 ± 00:02)	00:01 (00:05 ± 00:05)	00:01 (00:02 ± 00:00)
mljar-supervised	00:23 (00:25 ± 00:01)	00:41 (00:45 ± 00:01)	00:29 (00:32 ± 00:01)	00:49 (00:52 ± 00:03)	00:56 (00:59 ± 00:01)	00:37 (00:40 ± 00:02)	00:23 (00:28 ± 00:04)
NaiveAutoML	02:13 (04:13 ± 01:09)	00:01 (04:11 ± 01:42)	00:27 (00:28 ± 00:02)	02:04 (04:07 ± 01:10)	01:08 (01:14 ± 00:08)	00:57 (04:00 ± 01:32)	01:56 (01:57 ± 00:00)
PyCaret	00:07 (00:08 ± 00:00)	01:05 (01:12 ± 00:04)	00:16 (00:17 ± 00:00)	00:18 (00:19 ± 00:00)	00:54 (00:55 ± 00:00)	00:23 (00:24 ± 00:00)	00:09 (00:10 ± 00:00)
TPOT	05:00 (05:14 ± 00:17)	00:30 (01:57 ± 01:31)	00:21 (02:46 ± 01:49)	05:00 (05:05 ± 00:05)	05:01 (05:19 ± 00:13)	05:01 (05:08 ± 00:07)	05:02 (05:14 ± 00:12)

**Fig. 10:**
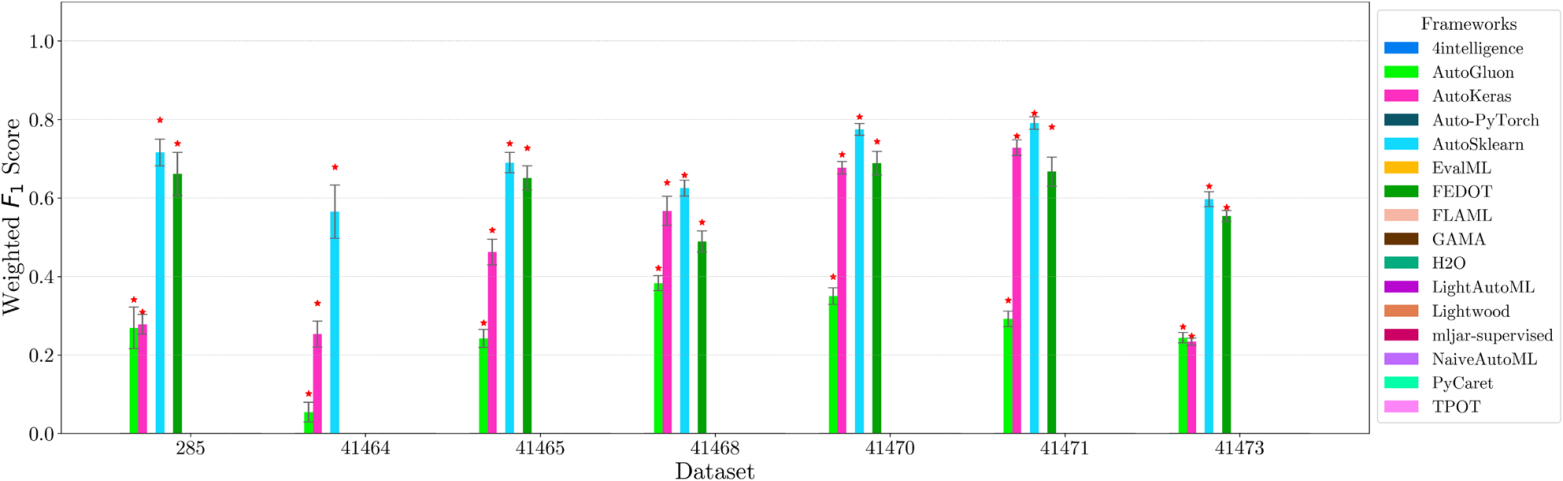
Multilabel (native) weighted $F_1$ score—maximum (mean ± std dev).

**Fig. 11:**
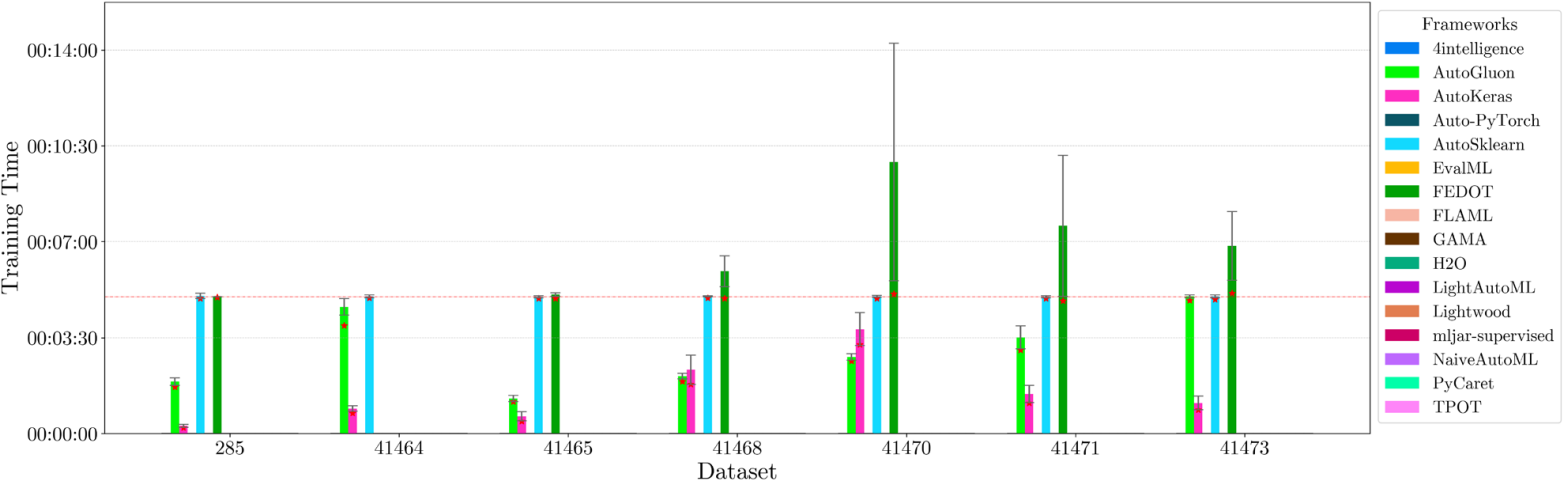
Multilabel (native) training time—minimum (mean ± std dev).

##### Performance analysis

Performance across multiclass datasets revealed notable differences in framework robustness, as shown in Table 7a and Fig. 8. On *cardiotocography* (ID 1466, 0.165/0.091), frameworks like AutoSklearn (1.000 ($1.000\pm 0.000$)) and PyCaret (1.000 ($0.999\pm 0.002$)) achieved high weighted $F_{1}$ scores with low variability, indicating reliable performance on high-complexity tasks. In contrast, H2O (0.915 ($0.706\pm 0.214$)) struggled to handle complex distributions consistently, while LightAutoML performed poorest (0.150 ($0.104\pm 0.022$)) on that dataset. Trends also varied on simpler datasets such as *contraceptive-method-choice* (ID 23, 0.018/0.529). Here, AutoSklearn (max 0.631, mean $0.569\pm 0.031$) proved more consistent, whereas LightAutoML (max 0.461, mean $0.415\pm 0.029$) demonstrated weaker performance and higher variability. Overall, many frameworks (e.g., AutoGluon, Auto-PyTorch, FEDOT, mljar-supervised, NaiveAutoML, TPOT) reached near-perfect $F_{1}$ on datasets such as *segment* (ID 36, 0.058/1.000), *wine-quality-red* (ID 40691, 0.041/0.015), and *car* (ID 40975, 0.014/0.054), underscoring the capacity of AutoML to excel on diverse multiclass tasks.

##### Training time analysis

Training times also varied significantly across frameworks and datasets, as shown in Table 7b and Fig. 9. Some frameworks (e.g., Auto-PyTorch, AutoSklearn, EvalML, TPOT) generally utilized the full 5-min budget or close to it, irrespective of dataset complexity, yielding top-tier $F_{1}$ scores but at the cost of longer, inflexible runtimes. In contrast, LightAutoML took only a few minutes on most datasets (e.g., 02:15 min for *contraceptive-method-choice* (ID 23, 0.018/0.529), 02:44 min for *cardiotocography* (ID 1466, 0.165/0.091)), though its performance was inconsistent. Meanwhile, AutoGluon, PyCaret, and mljar-supervised consistently completed tasks in well under a minute on simpler datasets—and even on more complex ones often finished in 1–3 min (e.g., AutoGluon ’s min 02:12 min on *wine-quality-red* (ID 40691, 0.041/0.015)), leveraging adaptive resource usage and ensembling. Finally, Lightwood reported extremely short runtimes (a few seconds) though with more variable performance.

##### Usability and scalability comparison

On *spambase* (ID 44, 0.025/0.650), frameworks that relied on extensive HPO required the full training budget to achieve high scores, while others produced comparable results in significantly less time. This supports findings that HPO through Bayesian optimization^38^ or genetic programming^49^ can increase training time without necessarily yielding substantial performance improvements. On *hill-valley* (ID 1479, 0.165/1.000), which contains 100 features, scalability differences were more apparent: some frameworks required the full training budget due to their reliance on exhaustive model selection^38^, whereas others completed training in a fraction of the time at the cost of lower $F_{1}$, illustrating the speed-accuracy trade-off in high-dimensional tasks^35,44^.

##### Biases, limitations, and failure modes

No multiclass dataset triggered a hard failure, yet two systematic weaknesses surfaced: (i) NAS-driven frameworks (AutoKeras, Auto-PyTorch) excelled on image-like *segment* (ID 36, 0.058/1.000) but showed limited generalization across tasks, revealing bias toward convolutional backbones; (ii) pipelines that blindly one-hot encode categoricals (H2O, LightAutoML) suffered severe accuracy drops (up to 0.80 in $F_{1}$) on *cardiotocography* (ID 1466, 0.165/0.091) and *yeast* (ID 181, 0.054/0.011), where rare classes combine with many sparse features, whereas CatBoost-based engines (FLAML, mljar-supervised) remained unaffected. Exhaustive-search systems (AutoSklearn, Auto-PyTorch, TPOT, GAMA) consistently consumed full 5-minute budgets, confirming classic speed-versus-performance trade-off.

##### Combined variability analysis

In multiclass classification, frameworks displayed differing levels of reliability in performance and training times. AutoSklearn and PyCaret consistently maintained low variability—exemplified on *segment* (ID 36, 0.058/1.000), where AutoSklearn reached 0.993 (0.985 ± 0.006) and PyCaret 0.994 (0.983 ± 0.005) with limited fluctuations. In contrast, LightAutoML and H2O reported greater variability, especially on *cardiotocography* (ID 1466, 0.165/0.091), where standard deviations in $F_{1}$ exceeded 0.020 and 0.200, respectively—suggesting sensitivity to parameter initialization or partial pipeline exploration.

##### Insights on complexity and imbalance

Imbalance dominated outcomes more than nominal complexity. The most complex set, *cardiotocography* (ID 1466, 0.165/0.091), yielded near-ceiling scores with robust encoders, while benign *yeast* (ID 181, 0.054/0.011) remained chief failure case for one-hot or linear pipelines. Even low-complexity, mid-skew *contraceptive-method-choice* (ID 23, 0.018/0.529) exposed $F_{1}$ gaps between exhaustive search engines and fast heuristics. Conversely, balanced but richer *segment* (ID 36, 0.058/1.000) posed little difficulty for NAS-based tools. Hence, the practical “hardness” surface is shaped by twin axes of categorical sparsity and minority-class share rather than feature-class ratio.

##### Conclusion

AutoSklearn and PyCaret balanced performance, efficiency, and robustness well on multiclass tasks. AutoGluon, mljar-supervised, and TPOT also demonstrated strong results, especially when time was limited and adaptive resource allocation proved beneficial. H2O and LightAutoML exhibited larger swings in performance, highlighting the need for tuning when faced with high-complexity data or subtle class imbalances. Encouragingly, *no* failures occurred on any dataset, underscoring these frameworks’ capabilities under controlled conditions, though real-world deployments still demand cautious preprocessing.

#### Multilabel (Native) scenario

Analyzing the native multilabel cases (i.e., without “label powerset” transformation) reveals framework compatibility and performance insights. Tables 8a and b, as well as Figures 10 and 11, present the experimental results.

**Table 8:** Performance summary for multilabel (Native) classification tasks.

(a) Multilabel (Native) weighted F1 score—maximum (mean ± std dev).							
Framework	Dataset						
	285	41,464	41,465	41,468	41,470	41,471	41,473
4intelligence	–	–	–	–	–	–	–
AutoGluon	0.341 (0.269 ± 0.053)	0.101 (0.054 ± 0.025)	0.282 (0.242 ± 0.023)	0.421 (0.383 ± 0.019)	0.399 (0.350 ± 0.021)	0.339 (0.292 ± 0.020)	0.272 (0.244 ± 0.013)
AutoKeras	0.309 (0.278 ± 0.025)	0.332 (0.253 ± 0.033)	0.518 (0.462 ± 0.033)	0.639 (0.567 ± 0.037)	0.711 (0.677 ± 0.016)	0.758 (0.728 ± 0.020)	0.248 (0.234 ± 0.009)
Auto-PyTorch	–	–	–	–	–	–	–
AutoSklearn	0.799 (0.716 ± 0.034)	0.679 (0.565 ± 0.068)	0.739 (0.690 ± 0.026)	0.658 (0.625 ± 0.020)	0.807 (0.775 ± 0.015)	0.816 (0.791 ± 0.016)	0.630 (0.597 ± 0.019)
EvalML	–	–	–	–	–	–	–
FEDOT	0.739 (0.661 ± 0.055)	–	0.727 (0.651 ± 0.031)	0.538 (0.489 ± 0.027)	0.744 (0.689 ± 0.030)	0.781 (0.667 ± 0.037)	0.576 (0.554 ± 0.014)
FLAML	–	–	–	–	–	–	–
GAMA	–	–	–	–	–	–	–
H2O	–	–	–	–	–	–	–
LightAutoML	–	–	–	–	–	–	–
Lightwood	–	–	–	–	–	–	–
mljar-supervised	–	–	–	–	–	–	–
NaiveAutoML	–	–	–	–	–	–	–
PyCaret	–	–	–	–	–	–	–
TPOT	–	–	–	–	–	–	–

(b) Multilabel (Native) training time—minimum (mean ± std dev).							
Framework	Dataset						
	285	41,464	41,465	41,468	41,470	41,471	41,473
4intelligence	–	–	–	–	–	–	–
AutoGluon	01:42 (01:54 ± 00:08)	03:57 (04:38 ± 00:18)	01:10 (01:17 ± 00:07)	01:54 (02:06 ± 00:06)	02:39 (02:48 ± 00:07)	03:03 (03:31 ± 00:25)	04:52 (05:01 ± 00:03)
AutoKeras	00:13 (00:17 ± 00:03)	00:45 (00:55 ± 00:06)	00:27 (00:38 ± 00:10)	01:48 (02:20 ± 00:32)	03:15 (03:49 ± 00:36)	01:07 (01:27 ± 00:19)	00:52 (01:07 ± 00:15)
Auto-PyTorch	–	–	–	–	–	–	–
AutoSklearn	04:56 (05:02 ± 00:06)	04:57 (05:01 ± 00:03)	04:56 (05:00 ± 00:02)	04:57 (05:01 ± 00:01)	04:56 (05:01 ± 00:02)	04:56 (05:00 ± 00:02)	04:54 (05:01 ± 00:03)
EvalML	–	–	–	–	–	–	–
FEDOT	04:59 (05:00 ± 00:00)	–	04:56 (05:05 ± 00:04)	04:56 (05:56 ± 00:34)	05:06 (09:55 ± 04:20)	04:51 (07:35 ± 02:34)	05:08 (06:51 ± 01:15)
FLAML	–	–	–	–	–	–	–
GAMA	–	–	–	–	–	–	–
H2O	–	–	–	–	–	–	–
LightAutoML	–	–	–	–	–	–	–
Lightwood	–	–	–	–	–	–	–
mljar-supervised	–	–	–	–	–	–	–
NaiveAutoML	–	–	–	–	–	–	–
PyCaret	–	–	–	–	–	–	–
TPOT	–	–	–	–	–	–	–

##### Performance analysis

Among the functioning frameworks, AutoSklearn consistently outperformed AutoKeras, AutoGluon, and FEDOT, as shown in Table 8a and Fig. 10. It achieved significantly higher $F_{1}$ scores across all datasets, with lower or comparable standard deviations, indicating greater robustness^3,32^. For instance, on *yeast* (ID 41473, 2.528/0.434), AutoSklearn reported a weighted $F_{1}$ score of $0.630\,(0.597\pm 0.019)$, outperforming both FEDOT with $0.576\,(0.554\pm 0.014)$ and AutoKeras with $0.248\,(0.234\pm 0.009)$. Even the best performer (AutoSklearn) peaked at just 0.63 on the hardest dataset, versus $>0.9$ on binary/multiclass tasks—underscoring that native multilabel remains largely unsolved. AutoGluon, while efficient on many binary or multiclass tasks^35^, struggled to surpass $F_{1}=0.421$ on *image* (ID 41468, 0.417/0.328), suggesting less effective internal strategies for multi-output data^3^. FEDOT performed moderately but remained sensitive to dataset complexity, and AutoKeras maintained relatively short training times but lagged in predictive accuracy on large or complex label spaces.

##### Training time analysis

As shown in Table 8b and Fig. 11, AutoSklearn consistently consumed the full 5-min budget, yielding thorough HPO and, consequently, higher $F_{1}$ scores^24,38^. By comparison, AutoKeras completed many tasks in under two minutes (e.g. 00:45–03:15), leveraging faster neural architecture exploration at the cost of fully optimized configurations^22^. AutoGluon displayed mixed times (1–5 min), reflecting adaptive resource usage^35^. Meanwhile, FEDOT sometimes hovered near 5 min (and even exceeded it on *reuters* (ID 41470, 0.981/0.197)), indicating less stable search strategies for higher-complexity problems^40^. These findings align with the principle that deeper model searches or ensemble methods typically yield better predictive performance but require proportionally more compute time^2,32^.

##### Usability and scalability comparison

From a usability perspective, frameworks with native multilabel support are rare; most either do not implement multi-output methods or rely on wrappers^3^. In this experiment, only AutoSklearn, AutoGluon, AutoKeras, and FEDOT produced valid native multilabel models. Among these, AutoSklearn ’s advanced Bayesian HPO often secured the highest $F_{1}$ but demanded full-time allocation^24,38^, while AutoKeras achieved faster builds via NAS but lower accuracy. AutoGluon and FEDOT landed in between, reflecting moderate performance and variable runtimes. This suggests that frameworks balancing thorough pipeline searches with efficient resource usage—via optimized cross-validation or automated ensembling^32,35^—offer more scalability on larger datasets under time constraints. However, the dearth of native multilabel solutions underscores that specialized tasks can limit an AutoML framework’s applicability in real-world scenarios.

##### Biases, limitations, and failure modes

Only four frameworks—AutoSklearn, AutoGluon, AutoKeras, and FEDOT—could train native multilabel models; the remaining eleven either lack multi-output estimators or crashed during pipeline construction. AutoSklearn ’s Binary-Relevance ensembles achieved the highest overall weighted $F_{1}$ but still reached only 0.799 (mean $0.716\pm 0.034$) on the highly imbalanced *flags* dataset (ID 285, 4.336/0.524), highlighting struggles with rare labels. Shared-backbone architectures of AutoGluon and AutoKeras also showed lower $F_{1}$ on *flags*, and FEDOT ’s performance varied widely, occasionally timing out under high label cardinality. These patterns illustrate how different native multilabel strategies yield varied robustness when faced with label sparsity.

##### Combined variability analysis

AutoSklearn and PyCaret consistently maintained low variability, exemplified on *segment* (ID 36, 0.058/1.000), where AutoSklearn reached $0.993\,(0.985\pm 0.006)$ and PyCaret $0.994\,(0.983\pm 0.005)$ with limited fluctuations. In contrast, LightAutoML and H2O reported greater variability, especially on *cardiotocography* (ID 1466, 0.165/0.091), where standard deviations in $F_{1}$ exceeded 0.020 and 0.200, respectively. Such variation can imply sensitivity to parameter initialization or partial pipeline exploration, implying further tuning might be necessary in practice.

##### Insights on complexity and imbalance

For native multilabel, rare-label frequency—not dimensionality—dictated variance. Every framework stumbled on *flags* (ID 285, 4.336/0.524), where half the labels appear in < 5% of samples, yet several handled the far larger and denser *birds* (ID 41464, 7.766/0.056) without incident. AutoSklearn ’s binary-relevance ensembles softened the blow but still posted zero recall on the sparsest tags, while shared-backbone models in AutoGluon/AutoKeras excelled on *image* (ID 41468, 0.417/0.328). These contrasts show that robustness scales with a tool’s ability to detect and weight ultra-minor labels rather than with search depth or feature count.

##### Conclusion

In native multilabel classification, AutoSklearn demonstrated the strongest combination of accuracy and stability, leveraging exhaustive model selection and HPO^24,38^. AutoKeras offered fast training but lower predictive power, while AutoGluon and FEDOT delivered mixed results and times. Only four of the 16 evaluated frameworks provided native multilabel support, leaving most unable to train directly on multi-output data. As a result, real-world users must often resort to label-powerset transformations or custom pipelines. This highlights a structural limitation within current AutoML, where tasks such as multilabel classification still require bespoke solutions to bridge persistent gaps in support^3^.

#### Multilabel (powerset) scenario

**Fig. 12:**
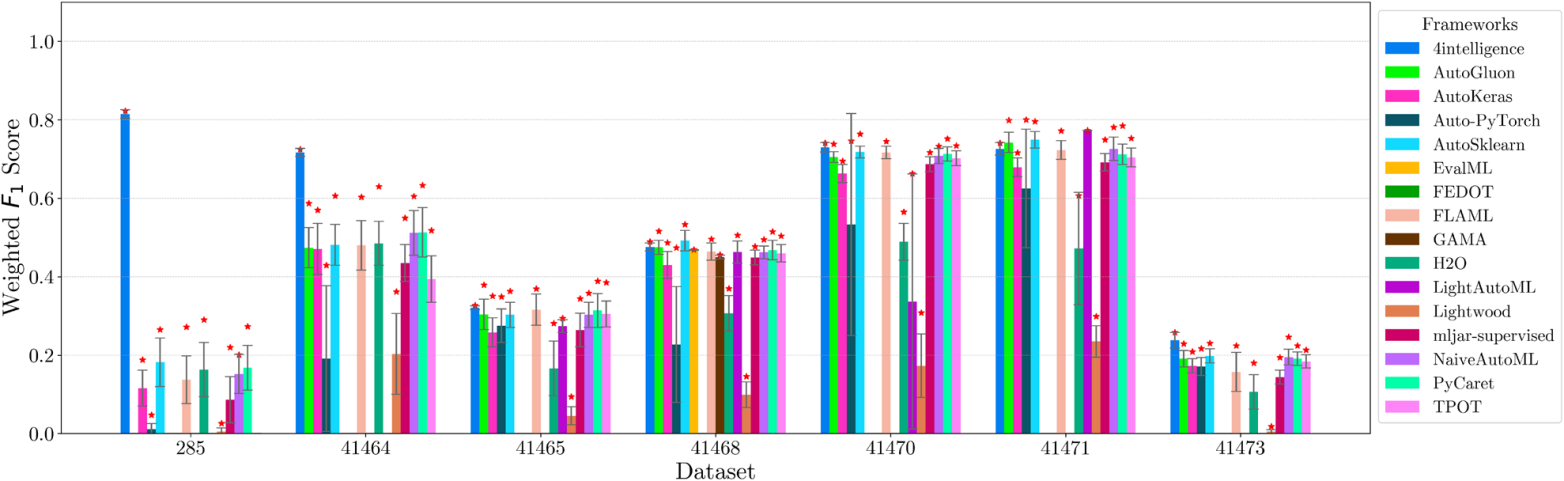
Multilabel (powerset) weighted $F_1$ score—maximum (mean ± std dev).

**Fig. 13:**
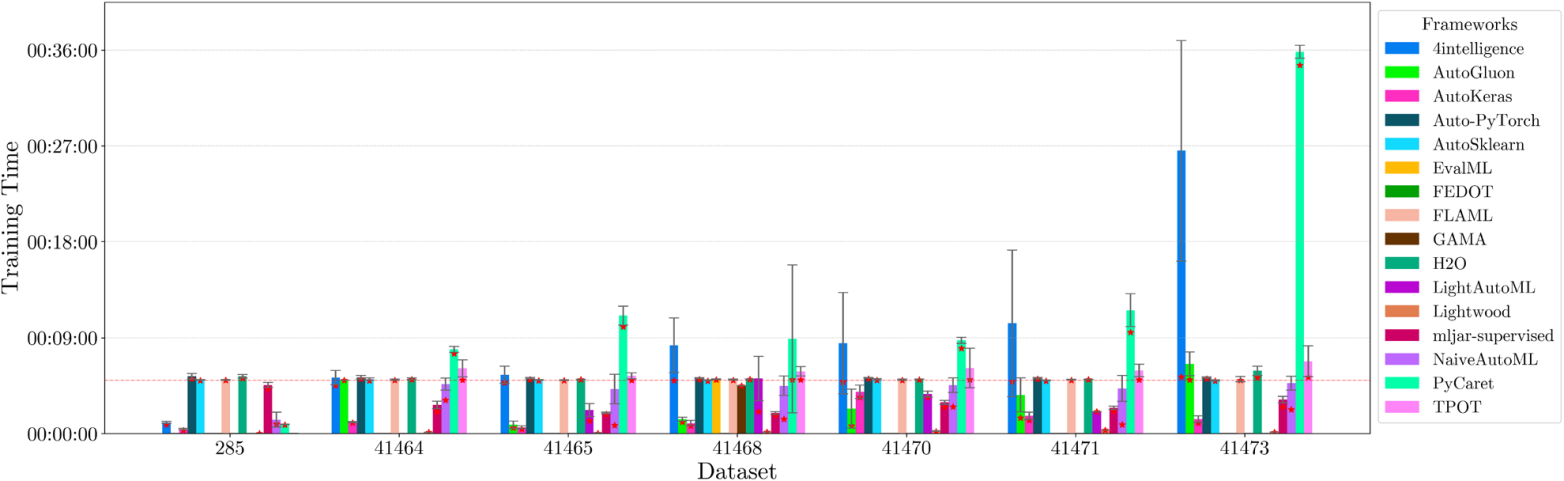
Multilabel (powerset) training time—minimum (mean ± std dev).

Moving on to the powerset-transformed multilabel scenarios, we observe varying performance and training efficiency among different frameworks, as shown in Tables 9a and b, as well as Figs. 12 and 13. All imbalance values reported in this section refer to the powerset-transformed label space, as defined in Table 4.

**Table 9:** Performance summary for multilabel (powerset) classification tasks.

(a) Multilabel (powerset) weighted F1 score—maximum (mean ± std dev).							
Framework	Dataset						
	285	41464	41465	41468	41470	41471	41473
4intelligence	0.823 (0.815 ± 0.011)	0.725 (0.717 ± 0.010)	0.327 (0.321 ± 0.005)	0.489 (0.476 ± 0.010)	0.741 (0.730 ± 0.012)	0.741 (0.726 ± 0.016)	0.258 (0.238 ± 0.020)
AutoGluon	–	0.587 (0.474 ± 0.051)	0.379 (0.304 ± 0.039)	0.516 (0.475 ± 0.018)	0.738 (0.705 ± 0.014)	0.799 (0.742 ± 0.026)	0.229 (0.191 ± 0.021)
AutoKeras	0.188 (0.116 ± 0.046)	0.570 (0.471 ± 0.065)	0.351 (0.258 ± 0.037)	0.487 (0.430 ± 0.034)	0.695 (0.663 ± 0.023)	0.716 (0.679 ± 0.024)	0.209 (0.173 ± 0.018)
Auto-PyTorch	0.047 (0.011 ± 0.014)	0.429 (0.191 ± 0.186)	0.349 (0.275 ± 0.043)	0.474 (0.227 ± 0.148)	0.746 (0.533 ± 0.283)	0.800 (0.625 ± 0.151)	0.217 (0.171 ± 0.023)
AutoSklearn	0.265 (0.182 ± 0.062)	0.606 (0.481 ± 0.052)	0.363 (0.303 ± 0.032)	0.533 (0.492 ± 0.026)	0.764 (0.718 ± 0.015)	0.796 (0.749 ± 0.021)	0.230 (0.198 ± 0.018)
EvalML	–	–	–	0.469 (0.468 ± 0.001)	–	–	–
FEDOT	–	–	–	–	–	–	–
FLAML	0.272 (0.137 ± 0.061)	0.603 (0.480 ± 0.063)	0.369 (0.316 ± 0.040)	0.496 (0.464 ± 0.022)	0.745 (0.717 ± 0.016)	0.772 (0.723 ± 0.024)	0.224 (0.157 ± 0.050)
GAMA	–	–	–	0.456 (0.450 ± 0.005)	–	–	–
H2O	0.290 (0.163 ± 0.069)	0.630 (0.485 ± 0.056)	0.281 (0.166 ± 0.070)	0.370 (0.307 ± 0.045)	0.565 (0.489 ± 0.047)	0.607 (0.472 ± 0.143)	0.180 (0.106 ± 0.044)
LightAutoML	–	–	0.294 (0.274 ± 0.016)	0.506 (0.463 ± 0.028)	0.663 (0.337 ± 0.325)	0.773 (0.773 ± 0.000)	–
Lightwood	0.026 (0.005 ± 0.009)	0.362 (0.203 ± 0.103)	0.094 (0.045 ± 0.023)	0.146 (0.099 ± 0.033)	0.308 (0.173 ± 0.081)	0.299 (0.235 ± 0.040)	0.018 (0.005 ± 0.005)
mljar-supervised	0.220 (0.086 ± 0.059)	0.550 (0.435 ± 0.047)	0.344 (0.264 ± 0.043)	0.477 (0.449 ± 0.019)	0.717 (0.687 ± 0.019)	0.749 (0.692 ± 0.022)	0.194 (0.144 ± 0.018)
NaiveAutoML	0.201 (0.152 ± 0.050)	0.605 (0.512 ± 0.057)	0.358 (0.303 ± 0.032)	0.495 (0.462 ± 0.016)	0.733 (0.708 ± 0.019)	0.781 (0.726 ± 0.030)	0.246 (0.195 ± 0.020)
PyCaret	0.273 (0.168 ± 0.057)	0.633 (0.513 ± 0.063)	0.389 (0.314 ± 0.043)	0.515 (0.468 ± 0.025)	0.752 (0.713 ± 0.018)	0.785 (0.712 ± 0.026)	0.224 (0.191 ± 0.017)
TPOT	–	0.518 (0.394 ± 0.059)	0.385 (0.305 ± 0.033)	0.504 (0.460 ± 0.022)	0.734 (0.702 ± 0.019)	0.753 (0.704 ± 0.024)	0.213 (0.184 ± 0.017)

(b) Multilabel (powerset) training time—minimum (mean ± std dev)							
Framework	Dataset						
	285	41464	41465	41468	41470	41471	41473
4intelligence	00:51 (01:03 ± 00:06)	04:31 (05:16 ± 00:40)	04:47 (05:32 ± 00:48)	04:59 (08:18 ± 02:34)	04:51 (08:30 ± 04:46)	04:54 (10:22 ± 06:53)	05:20 (26:33 ± 10:20)
AutoGluon	–	05:00 (05:00 ± 00:00)	00:33 (00:50 ± 00:21)	01:05 (01:22 ± 00:11)	00:44 (02:21 ± 01:50)	01:28 (03:38 ± 01:36)	05:03 (06:33 ± 01:07)
AutoKeras	00:18 (00:25 ± 00:05)	01:01 (01:05 ± 00:03)	00:26 (00:35 ± 00:08)	00:43 (00:59 ± 00:16)	03:25 (03:56 ± 00:38)	01:16 (01:40 ± 00:21)	01:00 (01:21 ± 00:19)
Auto-PyTorch	05:08 (05:23 ± 00:16)	05:07 (05:17 ± 00:10)	05:05 (05:13 ± 00:04)	05:05 (05:13 ± 00:03)	05:08 (05:14 ± 00:03)	05:07 (05:13 ± 00:04)	05:08 (05:16 ± 00:04)
AutoSklearn	04:59 (05:03 ± 00:02)	04:58 (05:07 ± 00:07)	04:58 (05:03 ± 00:02)	04:56 (05:01 ± 00:02)	05:00 (05:06 ± 00:04)	04:56 (05:00 ± 00:02)	04:57 (05:00 ± 00:03)
EvalML	–	–	–	05:03 (05:06 ± 00:02)	–	–	–
FEDOT	–	–	–	–	–	–	–
FLAML	05:00 (05:02 ± 00:02)	05:00 (05:03 ± 00:04)	04:59 (05:01 ± 00:02)	05:00 (05:04 ± 00:03)	05:00 (05:03 ± 00:04)	05:01 (05:04 ± 00:02)	05:00 (05:07 ± 00:15)
GAMA	–	–	–	04:29 (04:29 ± 00:00)	–	–	–
H2O	05:11 (05:23 ± 00:10)	05:04 (05:09 ± 00:05)	05:05 (05:06 ± 00:00)	05:06 (05:07 ± 00:00)	05:03 (05:07 ± 00:00)	05:05 (05:06 ± 00:00)	05:16 (05:54 ± 00:26)
LightAutoML	–	–	01:12 (02:13 ± 00:36)	02:03 (05:11 ± 02:05)	03:25 (03:43 ± 00:17)	02:04 (02:04 ± 00:00)	–
Lightwood	00:01 (00:01 ± 00:00)	00:07 (00:07 ± 00:00)	00:02 (00:03 ± 00:01)	00:08 (00:09 ± 00:00)	00:11 (00:14 ± 00:02)	00:19 (00:19 ± 00:00)	00:07 (00:08 ± 00:00)
mljar-supervised	04:15 (04:34 ± 00:14)	02:04 (02:42 ± 00:21)	01:48 (01:57 ± 00:04)	01:44 (01:56 ± 00:07)	02:29 (02:56 ± 00:11)	02:08 (02:25 ± 00:10)	02:31 (03:12 ± 00:18)
NaiveAutoML	00:55 (01:19 ± 00:42)	03:09 (04:39 ± 00:34)	00:46 (04:11 ± 01:23)	01:22 (04:30 ± 00:55)	02:30 (04:33 ± 00:41)	00:51 (04:15 ± 01:13)	02:15 (04:44 ± 00:39)
PyCaret	00:49 (00:52 ± 00:01)	07:30 (07:55 ± 00:16)	10:02 (11:05 ± 00:53)	05:05 (08:54 ± 06:57)	08:00 (08:46 ± 00:17)	09:31 (11:36 ± 01:32)	34:35 (35:50 ± 00:36)
TPOT	–	05:02 (06:08 ± 00:48)	05:01 (05:26 ± 00:17)	05:03 (05:51 ± 00:26)	05:05 (06:10 ± 01:52)	05:03 (05:56 ± 00:35)	05:18 (06:48 ± 01:27)

##### Performance analysis

Regarding the $F_{1}$ score, AutoSklearn consistently achieved the highest values across most datasets, demonstrating robustness and reliability^32,38^, as shown in Tables 9a and 12. For example, on *scene* (ID 41471, 0.787/0.003), AutoSklearn reached $0.796\,(0.749\pm 0.021)$, exceeding both FLAML with $0.772\,(0.723\pm 0.024)$ and Lightwood with $0.299\,(0.235\pm 0.040)$. Such superior performance often stems from comprehensive pipeline searches and ensemble-based optimization, which consistently yield higher accuracy but require more computational effort^2,24^. Meanwhile, FLAML and AutoGluon^35,41^ also delivered competitive $F_{1}$ results on datasets like *reuters* (ID 41470, 0.981/0.001), highlighting their potential for powerset-transformed tasks. However, frameworks like Lightwood^45^ and PyCaret^48^ persistently underperformed in predictive accuracy, likely due to shallower HPO strategies or limited feature-engineering capabilities^1^.

##### Training time analysis

Training efficiency varied considerably among frameworks, as shown in Fig. 13 and Table 9b. AutoSklearn exhibited stable training times near the 5-min limit, indicating a thorough optimization process. By contrast, Lightwood often completed tasks in under a minute, emphasizing minimal pipeline overhead at the cost of performance drops^3^. For instance, on *reuters* (ID 41470, 0.981/0.001), Lightwood finished in $00{:}14\pm 00{:}02$ but lagged behind AutoSklearn ’s $F_{1}$ by over 30%. PyCaret, conversely, suffered extended runtimes on high-complexity datasets, sometimes exceeding 5 min and negatively impacting usability for time-constrained scenarios. Such disparities confirm the trade-off: deeper HPO or ensembling can boost $F_{1}$ but require more computation, while faster frameworks may be appealing yet yield lower accuracy^24,32^.

##### Usability and scalability comparison

Label-powerset transformations inflate the effective number of classes, leading to pronounced imbalance and sparse label distributions^11,13^. Frameworks with robust ensembling or Bayesian HPO strategies (e.g., AutoSklearn^38^) generally cope better with this complexity, albeit at the cost of longer training times^2^. Meanwhile, tools like AutoGluon and AutoKeras balance speed and accuracy via adaptive model selection^35,41^, often excelling on moderately complex tasks without exhausting the time budget. In contrast, frameworks such as LightAutoML^44^, Lightwood^45^, or PyCaret^48^ scale well in runtime but exhibit steeper drops when confronted with large powerset label spaces or heavy imbalances^1,3^. Hence, user priorities—rapid prototyping vs. highest possible $F_{1}$—dictate which frameworks are most suitable in powerset scenarios (Table 10).

**Table 10:** Comparison of AutoML frameworks across classification scenarios in this and prior studies.

Scenario	Framework	This study (2025)	Truong et al. (2019)^2^	Wever et al. (2021)^3^	Gijsbers et al. (2024)^4^		Eldeeb et al. (2024)^61^
		$F_1$ $\uparrow$	$F_1$ $\uparrow$	Subset-$F_1$ $\uparrow$	AUC $\uparrow$	Log-loss $\downarrow$	$F_1$ $\uparrow$
Binary	4intelligence	0.606–1.000	–	–	–	–	–
	AutoGluon	0.787–1.000	–	–	0.796 ± 0.041	–	–
	AutoKeras	0.749–1.000	0.050–0.880	–	–	–	–
	Auto-PyTorch	0.597–1.000	–	–	–	–	–
	AutoSklearn	0.805–1.000	0.200–0.930	–	–	–	0.884 ± 0.132
	EvalML	0.780–1.000	–	–	–	–	–
	FEDOT	0.806–1.000	–	–	–	–	–
	FLAML	0.782–1.000	–	–	–	–	–
	GAMA	0.793–1.000	–	–	–	–	–
	H2O	0.380–0.985	0.550–0.900	–	–	–	–
	LightAutoML	0.231–0.636	–	–	–	–	–
	Lightwood	0.563–1.000	–	–	–	–	–
	mljar-supervised	0.791–1.000	–	–	–	–	–
	NaiveAutoML	0.783–1.000	–	–	–	–	–
	PyCaret	0.801–1.000	–	–	–	–	–
	TPOT	0.792–1.000	0.800–0.920	–	–	–	0.890 ± 0.121
	Darwin	–	0.900–0.940	–	–	–	–
	Ludwig	–	0.780–0.940	–	–	–	–
	ATM	–	–	–	–	–	0.899 ± 0.121
	AutoSklearn-e	–	–	–	–	–	0.883 ± 0.132
	AutoSklearn-m	–	–	–	–	–	0.873 ± 0.141
	AutoSklearn-v	–	–	–	–	–	0.870 ± 0.142
	AutoWeka	–	–	–	–	–	0.842 ± 0.165
	Recipe	–	–	–	–	–	0.764 ± 0.221
	SmartML-e	–	–	–	–	–	0.840 ± 0.165
	SmartML-m	–	–	–	–	–	0.826 ± 0.169

Scenario	Framework	This study (2025)	Truong et al. (2019)^2^	Wever et al. (2021)^3^	Gijsbers et al. (2024)^4^		Eldeeb et al. (2024)^61^
		$F_1$ $\uparrow$	$F_1$ $\uparrow$	Subset-$F_1$ $\uparrow$	AUC $\uparrow$	Log-loss $\downarrow$	$F_1$ $\uparrow$
Multiclass	4intelligence	0.552–1.000	–	–	–	–	–
	AutoGluon	0.615–1.000	–	–	–	0.456 ± 0.062	–
	AutoKeras	0.535–1.000	0.880–0.920	–	–	–	–
	Auto-PyTorch	0.589–1.000	–	–	–	–	–
	AutoSklearn	0.631–1.000	0.000–0.950	–	–	0.442 ± 0.026	0.884 ± 0.132
	EvalML	0.566–1.000	–	–	–	–	–
	FEDOT	0.596–1.000	–	–	–	–	–
	FLAML	0.604–1.000	–	–	–	–	–
	GAMA	0.618–1.000	–	–	–	–	–
	H2O	0.521–0.991	0.600–0.800	–	–	–	–
	LightAutoML	0.150–0.997	–	–	–	–	–
	Lightwood	0.401–0.994	–	–	–	–	–
	mljar-supervised	0.618–1.000	–	–	–	–	–
	NaiveAutoML	0.595–1.000	–	–	–	–	–
	PyCaret	0.619–1.000	–	–	–	–	–
	TPOT	0.613–1.000	0.850–0.880	–	–	–	0.890 ± 0.121
	Darwin	–	0.880–0.900	–	–	–	–
	Ludwig	–	0.780–0.820	–	–	–	–
	ATM	–	–	–	–	–	0.899 ± 0.121
	AutoWeka	–	–	–	–	–	0.842 ± 0.165
	Recipe	–	–	–	–	–	0.764 ± 0.221
	SmartML-e	–	–	–	–	–	0.840 ± 0.165
	SmartML-m	–	–	–	–	–	0.826 ± 0.169
Multilabel (Native)	AutoSklearn	0.630–0.816	–	–	–	–	–
	AutoKeras	0.248–0.758	–	–	–	–	–
	AutoGluon	0.101–0.421	–	–	0.101–0.421	–	–
	FEDOT	0.538–0.781	–	–	–	–	–
	SMAC	–	–	0.400–0.760	–	–	–
	HB	–	–	0.400–0.760	–	–	–
	BOHB	–	–	0.420–0.760	–	–	–
	GGP	–	–	0.410–0.730	–	–	–
	HTN-BF	–	–	0.430–0.790	–	–	–
	Random	–	–	0.420–0.790	–	–	–
Multilabel (Powerset)	4intelligence	0.258–0.823	–	–	–	–	–
	AutoGluon	0.229–0.799	–	–	–	–	–
	AutoKeras	0.188–0.716	–	–	–	–	–
	Auto-PyTorch	0.047–0.800	–	–	–	–	–
	AutoSklearn	0.230–0.796	–	–	–	–	–
	EvalML	0.469–0.469	–	–	–	–	–
	FLAML	0.224–0.772	–	–	–	–	–
	GAMA	0.456–0.456	–	–	–	–	–
	H2O	0.180–0.630	–	–	–	–	–
	LightAutoML	0.294–0.773	–	–	–	–	–
	Lightwood	0.018–0.362	–	–	–	–	–
	mljar-supervised	0.194–0.749	–	–	–	–	–
	NaiveAutoML	0.201–0.781	–	–	–	–	–
	PyCaret	0.224–0.785	–	–	–	–	–
	TPOT	0.213–0.753	–	–	–	–	–

For Gijsbers et al.^4^ multiclass log-loss we report the arithmetic mean of the dataset-level means and the unweighted pooled standard deviation across the panels for *contraceptive-method-choice* (ID 23), *segment* (ID 36), *vehicle* (ID 54), *yeast* (ID 181), and *car* (ID 40975).

Eldeeb et al.^61^ do not distinguish binary vs. multiclass outcomes; their $F_1$ ± SD results are therefore repeated for both scenarios in this table.

Acronyms/abbreviations used in the table: SMAC: Sequential Model-based Algorithm Configuration; HB: Hyperband; BOHB: Bayesian Optimization with Hyperband; GGP: Grammar-based Genetic Programming; HTN-BF: Hierarchical Task Network–Best-First search; Random: Random Search. 4 Row colors denote frameworks exclusive to specific prior benchmarks, omitted here due to their non-Python implementations or other study-specific constraints

##### Biases, limitations, and failure modes

After powerset transformation, effective class count explodes, exposing three recurrent issues: (i) memory-intensive GP/evolutionary frameworks (Auto-PyTorch, TPOT) struggled on high-cardinality sets *birds* (ID 41464, 7.766/0.003) and *yeast* (ID 41473, 2.528/0.004), achieving low precision on rare combinations; (ii) lighter Bayesian engines (FLAML, AutoGluon) finished within budget but lost precision on rare labels, reflecting limited cost-sensitive tuning; (iii) majority-class fallbacks in Lightwood and PyCaret produced sub-minute runtimes yet ignored over 90% of minority classes, yielding degenerate, mode-seeking predictions. FEDOT and GAMA failed on several powerset tasks due to memory or sparsity issues, whereas AutoSklearn ’s bagged one-vs-rest ensembles handled memory pressure and imbalance at the cost of a 5-min budget. These patterns confirm that robustness under label explosion demands either heavy compute (deep ensembles) or specialized imbalance-aware strategies—capabilities missing in many AutoML frameworks.

##### Combined variability analysis

AutoSklearn maintained low variability in both $F_{1}$ and training times, making it a robust choice for powerset-transformed tasks. On *reuters* (ID 41470, 0.981/0.001), it achieved $0.764\,(0.718\pm 0.015)$ at $05{:}06\pm 00{:}04$. In contrast, Lightwood displayed high instability, finishing swiftly ($<1$ min) but yielding widely fluctuating results. FLAML offered a middle ground, balancing competitive accuracy (i.e., $0.745\,(0.717\pm 0.016)$ on *reuters* (ID 41470, 0.981/0.001)) with moderate time usage, illustrating how partial yet efficient HPO can yield strong performance without monopolizing the runtime^41^. Hence, frameworks with heavier (or more methodical) optimization loops generally show lower performance variance, while faster solutions introduce the greater risk of suboptimal hyperparameter sets^2,24^.

##### Insights on complexity and imbalance

After powerset transformation, imbalance ratios plunge (< 0.05) yet effective classes skyrocket, producing thousands of near-singletons that cripple memory-hungry GP engines and majority baselines. Moderately complex *emotions* (ID 41465, 1.361/0.012) proved tougher than denser *reuters* (ID 41470, 0.981/0.001) because rare label combinations outnumber informative ones. AutoSklearn and FLAML contained long tail via bagged one-vs-rest or cost-aware boosting, whereas EvalML and FEDOT timed out on high-cardinality *yeast* (ID 41473, 2.528/0.004). Once label-space explosion sets in, success depends less on complexity than specialised imbalance-aware search, memory discipline.

##### Conclusion

In the multilabel powerset scenario, AutoSklearn generally dominated in $F_{1}$ performance, while FLAML and AutoGluon provided strong contenders with shorter average training times. Frameworks such as Lightwood and PyCaret, though quick on some datasets, often fell behind in predictive quality, illustrating the classic speed-versus-accuracy trade-off^3,32^. Additionally, a few tools failed to complete powerset tasks altogether due to label-sparsity-induced pipeline breakdowns^3,12^. The results confirm that deeper searches and robust ensembling yield higher accuracy but come at the cost of runtime, while less exhaustive approaches can be advantageous in time-sensitive contexts yet require careful consideration of label imbalance.

### Benchmark comparison with prior studies

To situate our results within the broader AutoML evaluation landscape, we compare our results against four representative benchmark studies (see Table 3):

- Truong et al. (2019)^2^: early analysis of binary and multiclass $F_1$-score ranges under varying class imbalance.
- Wever et al. (2021)^3^: subset-$F_1$ performance of advanced hyperparameter optimizers on native multilabel tasks.
- Gijsbers et al. (2024)^4^: AUC for binary and log-loss for multiclass across diverse datasets.
- Eldeeb et al. (2024)^61^: $F_1$ ± SD results under extended time budgets (10–240 min).

Note: we did not include Romero et al. (2022)^57^ in our comparison because their evaluation was performed on a completely disjoint set of datasets with no overlap to the ones used in this study (Table 4).

We summarize each study’s top-reported metrics (and runtimes, where available) alongside our 5 min budget $F_1$ scores in Table 3—highlighting advances in predictive quality and persistent gaps, particularly in multilabel classification; see the table notes for full definitions and aggregation details. Having established how our results compare to prior benchmarks, we now turn to a detailed examination—analyzing weighted $F_1$ scores across binary, multiclass, native multilabel, and powerset multilabel scenarios.

#### Binary scenario

In this study, most frameworks achieve high $F_1$ scores (0.75+ minimum), with several reaching perfect 1.0, except for LightAutoML (0.231–0.636) and H2O (0.380–0.985). Comparing with Truong et al.^2^, AutoSklearn shows improvement from their 0.200–0.930 range, while TPOT maintains consistent performance. Darwin (only in Truong’s study) showed impressive binary performance (0.900–0.940). Eldeeb et al.^61^ reported strong $F_1$ scores for TPOT (0.890 ± 0.121) and ATM (0.899 ± 0.121), suggesting these frameworks maintain reliability across different benchmark studies.

#### Multiclass scenario

Again, most frameworks reach perfect scores on some datasets, with AutoGluon, AutoSklearn, and PyCaret showing strong lower bounds (0.61+). Comparing across studies, AutoSklearn improved from Truong’s findings (0.000–0.950), while H2O still shows variability. Gijsbers et al.^4^ used different metrics (log-loss) where AutoSklearn slightly outperformed AutoGluon (0.442 ± 0.026 vs 0.456 ± 0.062, lower is better). Interestingly, frameworks like TPOT show consistent performance across three different studies spanning 2019–2025.

#### Multilabel (Native) scenario

Only AutoSklearn (0.630–0.816), AutoKeras (0.248–0.758), AutoGluon (0.101–0.421), and FEDOT (0.538–0.781) report native multilabel capabilities in this study. Wever et al.^3^ focused exclusively on multilabel classification with specialized algorithms like HTN-BF showing competitive performance (0.430–0.790). The contrast between studies highlights that multilabel classification has received less attention in AutoML benchmarking, with specialized approaches from Wever et al. potentially offering insights for improving general-purpose frameworks.

#### Multilabel (powerset) scenario

Our study shows 4intelligence leading (0.258–0.823) using the label powerset approach, with most frameworks achieving moderate performance but showing greater variability than in binary/multiclass scenarios. None of the previous studies evaluated multilabel powerset approaches, making this a unique contribution of the current research and highlighting a gap in AutoML evaluation literature for transformed multilabel problems. Our analysis reveals that the powerset transformation inherently exacerbates class imbalance by creating numerous rare label combinations, which increases modeling difficulty and explains the higher variance observed across frameworks and datasets compared to binary and multiclass scenarios.

Across scenarios and studies, AutoSklearn demonstrates the most consistent performance and has been thoroughly evaluated in multiple benchmarks. Frameworks show progressive improvements between Truong et al. and this study, particularly for binary and multiclass problems. However, multilabel classification remains challenging with lower $F_1$ scores across all frameworks and studies. The use of different evaluation metrics ($F_1$, AUC, log-loss), dataset selections, and runtime budgets (our 5-minute constraint versus Eldeeb et al.’s extended 10–240 min) across studies makes direct comparison challenging, but frameworks like TPOT and AutoSklearn demonstrate reliable performance across different research efforts, time periods, and computational constraints. Gijsbers’ use of AUC and log-loss provides complementary performance insights that align with $F_1$-based rankings, further validating the overall performance trends observed across different evaluation approaches.

### Overall findings and insights

The analysis reveals that no single AutoML framework excels across all scenarios. AutoSklearn consistently demonstrated strong performance across binary, multiclass, and multilabel tasks (both native and powerset), making it a reliable choice for a wide range of classification problems. Its combination of high $F_{1}$ scores, robustness (low variability across datasets), and relatively stable training times underscores its versatility. However, it often consumes the full 5-min budget^38^, reflecting deeper hyperparameter searches that can be prohibitive in strict time-sensitive contexts^2^.

Frameworks like Lightwood and AutoKeras offered faster training times but showed trade-offs in accuracy due to simpler pipeline strategies and more limited hyperparameter exploration^1,45^. Meanwhile, dataset complexity played a significant role in framework performance, though traditional complexity metrics alone did not fully capture real-world challenges such as label imbalance or complex feature interactions. In multiclass tasks, some frameworks exhibited counterintuitive results on ostensibly simpler datasets (e.g., 23) while performing well on more complex ones (e.g., 1466), suggesting domain-specific factors can override nominal complexity^32^.

Challenges like sparse labels, imbalanced data, and preprocessing limitations were particularly notable in multilabel scenarios. In the native multilabel setting, only AutoSklearn, AutoGluon, AutoKeras, and FEDOT produced valid results, highlighting the scarcity of built-in multi-output support^3^. Meanwhile, powerset transformations further exacerbated class imbalance, leading to partial failures for frameworks like GAMA and FEDOT. EvalML also struggled on certain transformations, reflecting the need for more robust data-handling strategies (e.g., label subset smoothing, meta-label modeling)^12,13^.

Table 11 summarizes the key findings, challenges, and recommendations. It underscores how advanced hyperparameter optimization techniques (Bayesian or genetic)^38,49^ can yield top scores at the expense of runtime, while faster methods sacrifice some accuracy. Across binary, multiclass, and multilabel tasks, frameworks like AutoSklearn proved exceptionally consistent but time-consuming, whereas LightAutoML and Lightwood frequently finished in minutes (or seconds) but faced larger performance drops on high-dimensional or imbalanced datasets^44,45^. Striking a balance between thoroughness and efficiency remains a central challenge in AutoML design.

By addressing these gaps—improving native multilabel support, handling imbalanced classes, and refining preprocessing pipelines—AutoML frameworks can become more versatile and reliable. This is particularly important for real-world tasks where data quality is inconsistent and resource constraints are stringent^3,32^. The recommendations in Table 11 provide actionable steps to guide framework development toward low-variability, high-adaptability solutions for the wide range of classification challenges encountered in practice.

**Table 11:** Summary of the results and discussion.

Scenario	Key findings	Challenges	Recommendations
Binary	Advanced HPO approaches yielded top performance but required more time. Some faster frameworks or minimal pipelines struggled on complex or missing data and occasionally failed on certain tasks	Rigid preprocessing and insufficient handling of missing data or class imbalance limited performance and led to failures.	Adopt robust data encoding, improve imbalance mitigation, and enable adaptive model selection to address diverse complexities and avoid execution errors
Multiclass	A few frameworks consistently achieved strong results, while others showed unexpected drops on simpler data. No framework failed to run, though performance variability was substantial	Maintaining stable accuracy across varied distributions was difficult, and certain frameworks always used the full-time budget or saw large accuracy swings despite quick runs.	Incorporate adaptive ensembling or selective search to handle different data complexities effectively without monopolizing runtime or suffering marked performance drops
Multilabel (Native)	Only a limited set of frameworks supported native multi-output classification; one generally excelled in accuracy, while another was faster but less accurate	Sparse label sets, limited native support, and inconsistent training times reduced reliability, with many frameworks providing no results at all	Enhance native multilabel capabilities to cope with label sparsity and ensure consistent optimization loops for stable performance
Multilabel (Powerset)	More exhaustive pipelines or ensembling achieved higher scores but demanded longer training. Some frameworks finished rapidly but showed significantly lower accuracy or failed under extreme label inflation	Label powerset transformations exposed imbalance and sparse label combinations, causing pipeline instability and partial failures in certain tools	Adopt specialized balancing or meta-label methods to handle expanded label sets and refine search algorithms to stay robust under label inflation
General	No single tool dominated all tasks. Comprehensive search approaches delivered higher accuracy but often used the entire time limit, while faster methods risked significant degradation on challenging data	Handling real-world data characteristics, such as missing features and label imbalance, remained a common obstacle, and traditional complexity metrics did not fully capture domain-level issues	Employ resilient pipelines that combine flexible search with advanced preprocessing and domain-aware strategies, balancing thoroughness against strict time constraints

In summary, each framework balanced accuracy, speed, and ease of use differently across binary, multiclass, and multilabel tasks. The next section provides a statistical analysis for deeper insights into their real-world applicability.

## Statistical analysis

A robust, three-stage statistical analysis was conducted to compare AutoML frameworks with respect to both predictive performance ($F_1$ score) and computational efficiency (training time). In view of the heterogeneous nature of the datasets, classification tasks, and framework behaviors, all decisions regarding the selection of statistical tests and post-hoc comparisons were made automatically, guided by programmatically assessed properties such as normality (e.g., Shapiro–Wilk), variance homogeneity (e.g., Levene’s test), and sphericity (Mauchly’s test). This approach minimized the risk of subjective choices and ensured consistent, repeatable evaluations despite the complexity of the data.

The analysis was organized into three parts:

- Per-dataset analysis (“Per-dataset analysis” section): Each dataset was handled as a standalone case to highlight framework-specific strengths or deficiencies. We tested for normality and variance homogeneity, then applied either a parametric method (ANOVA with Tukey’s HSD) or a non-parametric counterpart (Kruskal–Wallis with Dunn’s post-hoc). This revealed how each framework performed on every individual dataset, unveiling nuances possibly masked by aggregate analyses.
- Across-datasets analysis (“Across-datasets analysis” section): We then aggregated results within each classification scenario (binary, multiclass, multilabel) to identify overarching performance trends. Mean $F_1$ and median training time were tested for normality (Kolmogorov–Smirnov if necessary) before employing either a repeated-measures ANOVA (with Greenhouse–Geisser correction if sphericity was violated) or the Friedman test if parametric assumptions did not hold. This stage provided a broader perspective on how frameworks fared in each scenario as a whole.
- All-datasets analysis (“All-datasets analysis)” section: Lastly, a global comparison was performed by pooling all datasets across all classification types, creating a unified view of framework performance. Given frequent departures from sphericity, a Friedman test was chosen, followed by Nemenyi post-hoc tests for pairwise framework comparisons. This produced a final, scenario-agnostic ranking of methods.

Throughout the analysis, a significance level of $\alpha = 0.05$ was maintained, following standard hypothesis testing conventions^99^, ensuring that the observed differences in framework performance were statistically meaningful. To further strengthen the reliability and reproducibility of these findings, we implemented all statistical tests in Python 3.10 using well-established libraries: scipy^100^ for core hypothesis testing, statsmodels^101^ for advanced model diagnostics, scikit-posthocs^102^ for multiple-comparisons procedures, and pingouin^103^ for additional metrics.

By structuring the analysis into per-dataset, across-datasets, and all-datasets stages, we capture nuances at increasing levels of aggregation, thereby addressing potential confounding factors like dataset complexity or class imbalance. The following subsections detail each step, presenting key results and discussing potential research and practical implications.

### Per-dataset analysis

For each dataset, we assessed the performance of multiple AutoML frameworks based on two key metrics: $F_1$ Score (predictive performance) and Training Time (computational efficiency). Because each framework–dataset combination had at most 20 trials, the Shapiro–Wilk test^104^ was used to assess normality (appropriate for smaller sample sizes), and Levene’s test^105^ determined whether variances were homogeneous across frameworks.

When both normality and homogeneity held, we proceeded with parametric statistical tests; otherwise, we used non-parametric methods. To improve normality and stabilize variance, we applied two transformations. First, for bounded $F_1$ values in $[0,1]$, we used the arcsine square root transformation^106^, $X' = \arcsin (\sqrt{X})$. Second, for right-skewed Training Time data, we employed a logarithmic transform^107^, $X' = \log (1 + X)$.

If the transformed data satisfied normality and homoscedasticity, we ran a one-way ANOVA^99^ to test for overall significance ($p<0.05$). In cases where ANOVA was significant, Tukey’s Honestly Significant Difference (HSD)^108^ identified pairwise differences among frameworks. Where assumptions were violated, we relied on the Kruskal-Wallis test^109^, a rank-based alternative to ANOVA, and then used Dunn’s post-hoc procedure^110^ with Holm’s correction^111^ to account for multiple comparisons.

#### Results

**Fig. 14:**
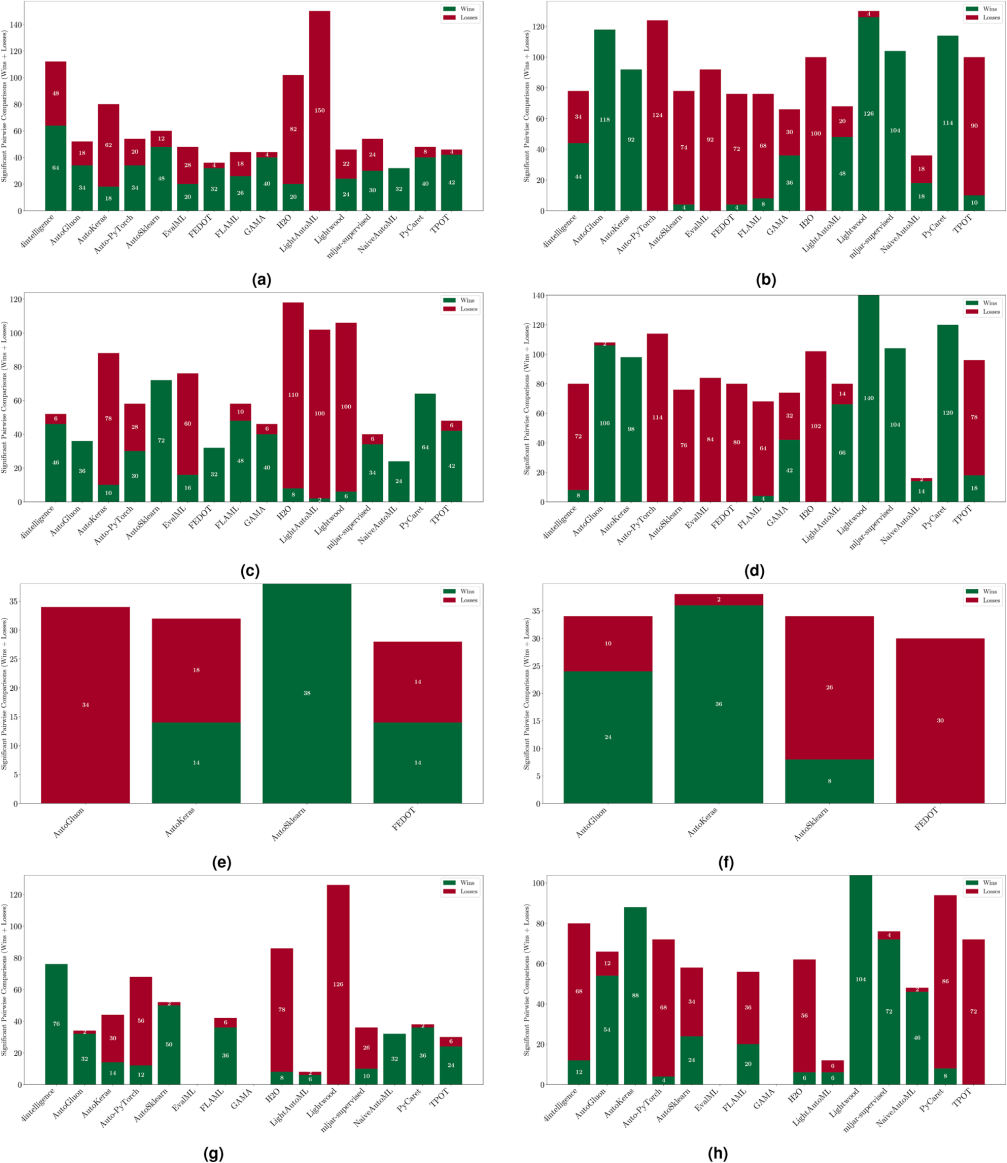
Win–loss stacked bar plots for each scenario. Each bar represents the number of datasets where a framework achieved statistically significant wins (green) or losses (red) relative to others, shown separately for $F_1$ score and training time.

**Fig. 15:**
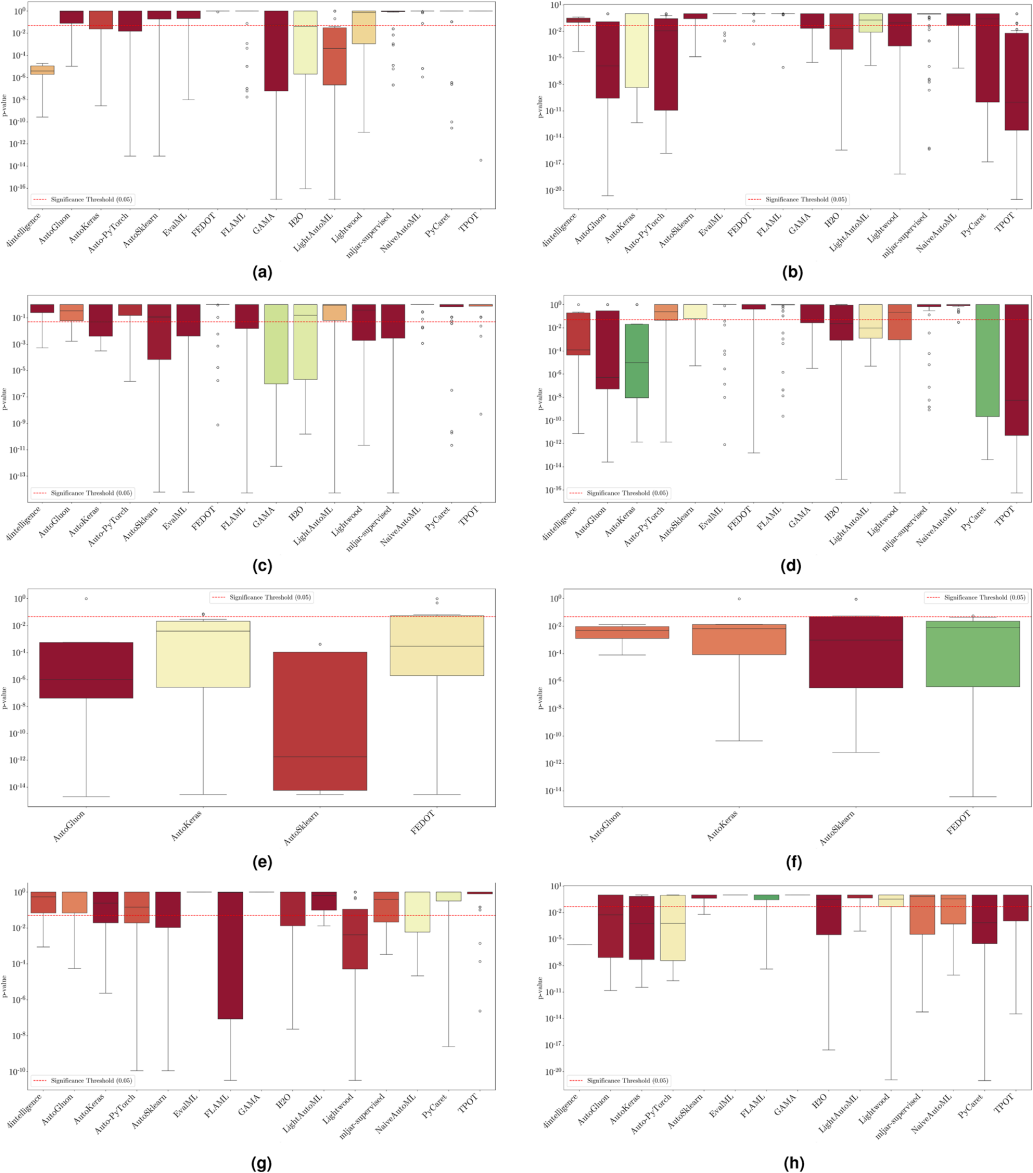
Post-hoc *p*-value distributions for each scenario, illustrating pairwise significance among frameworks for $F_1$ Score and Training Time. Colors indicate different significance levels, with green for strong significance, yellow for moderate, and red for weak. The red dashed line marks the $\alpha$ threshold.

Table 12 summarizes the statistical test results for $F_1$ Score and Training Time in the per-dataset setting. Win–Loss matrices were constructed for each dataset to show how often one framework significantly outperformed another, and these were aggregated by scenario into Win–Loss stacked bar plots, as shown in Fig. 14. Additionally, the distribution of post-hoc $p$-values is illustrated via boxplots in Fig. 15, highlighting the strength of observed differences in both $F_1$ Score and Training Time. Most datasets did not meet the criteria for parametric methods, so Kruskal–Wallis with Dunn’s post-hoc predominated. Nonetheless, a few cases (notably in some multilabel datasets) allowed ANOVA and Tukey’s HSD, demonstrating how differing dataset characteristics can necessitate different statistical procedures.

**Table 12:** Statistical test results.

(a)Statistical test results for $F_1$ score. Each row shows normality checks, variance checks, the selected method, and whether the differences were significant. Note that dataset 41465 met both normality and homoscedasticity assumptions, allowing an ANOVA + Tukey approach									
Scenario	Dataset	Normal pairs	Normal?	Homoscedastic Pairs	Homoscedastic?	Parametric?	Method	*p*-value	Significant?
Binary	1462	15/120	No	0/120	No	No	Kruskal–Wallis + Dunn	$3.1\times 10^{-39}$	Yes
	1479	36/120	No	0/120	No	No	Kruskal–Wallis + Dunn	$1.6\times 10^{-53}$	Yes
	1510	21/120	No	120/120	Yes	No	Kruskal–Wallis + Dunn	$1.3\times 10^{-17}$	Yes
	31	105/120	No	120/120	Yes	No	Kruskal–Wallis + Dunn	$8.3\times 10^{-24}$	Yes
	37	66/120	No	0/120	No	No	Kruskal–Wallis + Dunn	$1.4\times 10^{-18}$	Yes
	40945	36/66	No	0/66	No	No	Kruskal–Wallis + Dunn	$6.8\times 10^{-27}$	Yes
	44	66/120	No	0/120	No	No	Kruskal–Wallis + Dunn	$4.5\times 10^{-33}$	Yes
Multiclass	1466	36/120	No	0/120	No	No	Kruskal–Wallis + Dunn	$1.3\times 10^{-45}$	Yes
	181	91/120	No	0/120	No	No	Kruskal–Wallis + Dunn	$1.7\times 10^{-34}$	Yes
	23	91/120	No	0/120	No	No	Kruskal–Wallis + Dunn	$1.4\times 10^{-25}$	Yes
	36	91/120	No	0/120	No	No	Kruskal–Wallis + Dunn	$2.0\times 10^{-11}$	Yes
	40691	105/120	No	0/120	No	No	Kruskal–Wallis + Dunn	$1.5\times 10^{-31}$	Yes
	40975	45/120	No	0/120	No	No	Kruskal–Wallis + Dunn	$4.1\times 10^{-34}$	Yes
	54	91/120	No	0/120	No	No	Kruskal–Wallis + Dunn	$3.2\times 10^{-28}$	Yes
Multilabel (Native)	285	3/6	No	0/6	No	No	Kruskal–Wallis + Dunn	$2.0\times 10^{-9}$	Yes
	41464	3/3	Yes	0/3	No	No	Kruskal–Wallis + Dunn	$4.1\times 10^{-12}$	Yes
	41465	6/6	Yes	6/6	Yes	Yes	ANOVA + Tukey	$4.7\times 10^{-60}$	Yes
	41468	6/6	Yes	0/6	No	No	Kruskal–Wallis + Dunn	$9.2\times 10^{-15}$	Yes
	41470	6/6	Yes	0/6	No	No	Kruskal–Wallis + Dunn	$1.7\times 10^{-14}$	Yes
	41471	3/6	No	6/6	Yes	No	Kruskal–Wallis + Dunn	$3.5\times 10^{-15}$	Yes
	41473	6/6	Yes	6/6	Yes	Yes	ANOVA + Tukey	$3.1\times 10^{-80}$	Yes
Multilabel (Powerset)	285ps	15/45	No	0/45	No	No	Kruskal–Wallis + Dunn	$4.4\times 10^{-23}$	Yes
	41464	36/66	No	0/66	No	No	Kruskal–Wallis + Dunn	$6.7\times 10^{-25}$	Yes
	41465	66/78	No	0/78	No	No	Kruskal–Wallis + Dunn	$5.6\times 10^{-22}$	Yes
	41468	45/105	No	0/105	No	No	Kruskal–Wallis + Dunn	$3.2\times 10^{-26}$	Yes
	41470	36/78	No	0/78	No	No	Kruskal–Wallis + Dunn	$6.5\times 10^{-28}$	Yes
	41471	21/78	No	0/78	No	No	Kruskal–Wallis + Dunn	$4.6\times 10^{-26}$	Yes
	41473	21/66	No	0/66	No	No	Kruskal–Wallis + Dunn	$1.0\times 10^{-27}$	Yes

(b)Statistical test results for training time. Rows show normal/variance checks, chosen method, and resulting significance									
Scenario	Dataset	Normal pairs	Normal?	Homoscedastic pairs	Homoscedastic?	Parametric?	Method	*p*-value	Significant?
Binary	1462	15/120	No	0/120	No	No	Kruskal–Wallis + Dunn	$2.9\times 10^{-51}$	Yes
	1479	36/120	No	0/120	No	No	Kruskal–Wallis + Dunn	$1.2\times 10^{-53}$	Yes
	1510	6/120	No	0/120	No	No	Kruskal–Wallis + Dunn	$3.3\times 10^{-50}$	Yes
	31	28/120	No	0/120	No	No	Kruskal–Wallis + Dunn	$1.4\times 10^{-52}$	Yes
	37	28/120	No	0/120	No	No	Kruskal–Wallis + Dunn	$2.9\times 10^{-52}$	Yes
	40945	28/66	No	0/66	No	No	Kruskal–Wallis + Dunn	$2.0\times 10^{-38}$	Yes
	44	36/120	No	0/120	No	No	Kruskal–Wallis + Dunn	$2.5\times 10^{-53}$	Yes
Multiclass	1466	36/120	No	0/120	No	No	Kruskal–Wallis + Dunn	$5.9\times 10^{-51}$	Yes
	181	6/120	No	0/120	No	No	Kruskal–Wallis + Dunn	$5.2\times 10^{-47}$	Yes
	23	15/120	No	0/120	No	No	Kruskal–Wallis + Dunn	$1.1\times 10^{-49}$	Yes
	36	21/120	No	0/120	No	No	Kruskal–Wallis + Dunn	$6.7\times 10^{-47}$	Yes
	40691	3/120	No	0/120	No	No	Kruskal–Wallis + Dunn	$1.3\times 10^{-49}$	Yes
	40975	36/120	No	0/120	No	No	Kruskal–Wallis + Dunn	$3.5\times 10^{-47}$	Yes
	54	45/120	No	0/120	No	No	Kruskal–Wallis + Dunn	$3.4\times 10^{-47}$	Yes
Multilabel (Native)	285	0/6	No	0/6	No	No	Kruskal–Wallis + Dunn	$6.7\times 10^{-12}$	Yes
	41464	1/3	No	0/3	No	No	Kruskal–Wallis + Dunn	$8.7\times 10^{-11}$	Yes
	41465	3/6	No	0/6	No	No	Kruskal–Wallis + Dunn	$3.7\times 10^{-15}$	Yes
	41468	3/6	No	0/6	No	No	Kruskal–Wallis + Dunn	$1.4\times 10^{-13}$	Yes
	41470	0/6	No	0/6	No	No	Kruskal–Wallis + Dunn	$1.2\times 10^{-15}$	Yes
	41471	1/6	No	0/6	No	No	Kruskal–Wallis + Dunn	$1.3\times 10^{-15}$	Yes
	41473	3/6	No	0/6	No	No	Kruskal–Wallis + Dunn	$3.9\times 10^{-14}$	Yes
Multilabel (Powerset)	285ps	15/45	No	0/45	No	No	Kruskal–Wallis + Dunn	$1.6\times 10^{-30}$	Yes
	41464	10/66	No	0/66	No	No	Kruskal–Wallis + Dunn	$1.8\times 10^{-29}$	Yes
	41465	10/78	No	0/78	No	No	Kruskal–Wallis + Dunn	$2.2\times 10^{-39}$	Yes
	41468	21/105	No	0/105	No	No	Kruskal–Wallis + Dunn	$3.2\times 10^{-37}$	Yes
	41470	3/78	No	0/78	No	No	Kruskal–Wallis + Dunn	$1.1\times 10^{-37}$	Yes
	41471	6/78	No	0/78	No	No	Kruskal–Wallis + Dunn	$3.0\times 10^{-38}$	Yes
	41473	3/66	No	0/66	No	No	Kruskal–Wallis + Dunn	$9.2\times 10^{-42}$	Yes

#### Statistical interpretation

Before addressing the research questions in detail, we provide an overview of how the per-dataset results should be interpreted. Each dataset is treated independently to uncover framework-specific strengths and weaknesses in different settings. Because transformations (arcsine or logarithmic) and test choices (parametric vs. non-parametric) can vary from one dataset to another, the following questions and findings capture scenario-by-scenario observations rather than an overall ranking.

RQ1: What statistical test was used for each dataset, and why?

For most datasets, normality and variance assumptions were violated, prompting the Kruskal–Wallis test over ANOVA. As shown in Tables 12a and b, all datasets yielded statistically significant differences in both $F_1$ Score and Training Time. Extremely small $p$-values (e.g., $3.1 \times 10^{-39}$) point to pronounced framework performance disparities. When assumptions were met (e.g., dataset 41465), ANOVA + Tukey’s HSD was applied.

RQ2: Which frameworks perform best and worst for each dataset, in terms of $F_1$ Score and Training Time?

- Best/Worst in $F_1$ Score: Binary tasks show that 4intelligence and AutoSklearn often secured the most wins, while LightAutoML ranked poorly (Fig. 14a). In multiclass settings, AutoSklearn dominated, with PyCaret close behind, but H2O and LightAutoML struggled (Fig. 14c). For multilabel (native), AutoSklearn again excelled, whereas AutoGluon recorded the most losses (Fig. 14e). In the powerset approach, 4intelligence led with more significant wins, while Lightwood and H2O lagged (Fig. 14g).
- Best/Worst in Training Time: Lightwood and AutoGluon were quickest on binary datasets, while Auto-PyTorch and H2O were slowest (Fig. 14b). This pattern continued in multiclass tasks, with Lightwood and PyCaret being the most efficient and Auto-PyTorch/H2O again slower (Fig. 14d). For multilabel (native), AutoKeras proved to be the fastest, whereas FEDOT was consistently slow (Fig. 14f). Finally, Lightwood and AutoKeras also led in powerset tasks, whereas PyCaret and TPOT remained the slowest (Fig. 14h).

RQ3: How strong and consistent are the performance differences among frameworks across datasets?

- $F_1$ Score consistency: Across binary tasks, post-hoc $p$-values were largely below $10^{-10}$, confirming robust differences (Fig. 15a). AutoSklearn and 4intelligence were repeatedly strong, while LightAutoML showed higher $p$-value medians (i.e., weaker performance). In multiclass tasks, AutoSklearn and PyCaret again featured the lowest $p$-values, whereas H2O and Lightwood indicated higher variance (Fig. 15c). Meanwhile, multilabel scenarios often showed broader spreads in significance; AutoSklearn stayed near the top, but AutoGluon had varying results (Figs. 15e , 15g).
- *Training time consistency* For binary tasks, Lightwood and AutoGluon repeatedly showed small medians, while Auto-PyTorch and H2O were slower with high variance (Fig. 15b). Similar patterns emerged in multiclass (Fig. 15d), whereas in multilabel (native) tasks, AutoKeras was the fastest with tight whiskers, and FEDOT was slow (Fig. 15f). Lastly, in powerset tasks, Lightwood and AutoKeras again ranked as the most efficient, while PyCaret and TPOT performed poorly (Fig. 15h).

These results show that certain frameworks (AutoSklearn, 4intelligence) frequently lead in $F_1$ Score, whereas others (Lightwood, AutoGluon) commonly excel in Training Time. A few (H2O, LightAutoML) display less stable performance across datasets. Because these findings do not provide a definitive ranking over multiple tasks, the subsequent sections aggregate results to identify more general and robust patterns.

### Across-datasets analysis

While the per-dataset analysis provided insights into individual datasets, we also assessed framework performance across multiple datasets within each scenario to derive broader rankings. Specifically, we aggregated results per dataset by computing the mean $F_1$ Score for each framework (suitable for near-symmetric distributions) and the median Training Time (to mitigate skewness) before comparing frameworks across all datasets in a given scenario.

Before deciding on a statistical test, we checked the aggregated data for normality. When the number of datasets was fewer than 50, the Shapiro–Wilk test^104^ was used; for dataset counts of 50 or more, the Kolmogorov–Smirnov test^112^ was applied. If normality was violated ($p < 0.05$), we proceeded with the Friedman test^113^, a rank-based alternative that does not assume normality or sphericity. Conversely, if normality was upheld, we ran Mauchly’s test^114^ to examine sphericity. A satisfactory sphericity result ($p \ge 0.05$) permitted the use of Repeated-Measures ANOVA (RM-ANOVA)^115^, whereas sphericity violations led to applying the Greenhouse-Geisser correction^116^ for RM-ANOVA. If Mauchly’s test failed altogether (e.g., returning NaN due to a singular matrix or excessive missing data), the procedure automatically reverted to the Friedman test.

After determining the primary test (RM-ANOVA or Friedman), we selected a post-hoc method based on both the statistical approach and the number of pairwise comparisons. Under RM-ANOVA, we employed pairwise $t$-tests with Holm–Bonferroni correction^111^ if there were fewer than 10 comparisons or the Benjamini–Hochberg correction^117^ if 10 or more. For the Friedman test, the Conover test^118^ with Holm–Bonferroni correction was used if there were fewer than 10 comparisons (roughly five frameworks), whereas the Nemenyi test^119^ was applied when 10 or more comparisons existed, since Nemenyi does not require an additional correction.

Framework performance within each scenario was then expressed via an *average rank*. Under RM-ANOVA, each dataset assigned ranks to frameworks according to higher $F_1$ Scores (better, i.e., lower rank) and lower Training Times (also better, i.e., lower rank). These ranks were then averaged across all datasets in that scenario. When the Friedman test was used, the same ranking approach was conducted according to the non-parametric procedure inherent to Friedman. In both cases, a lower final rank indicated superior overall performance. If the primary test revealed no statistically significant difference ($p \ge 0.05$), the frameworks were simply reported rather than ranked.

Table 13 summarizes this decision process, detailing the thresholds for normality, sphericity checks, and the respective primary/post-hoc tests that govern how frameworks were compared across multiple datasets.

**Table 13:** Statistical decision framework for AutoML comparison across datasets.

Step	Decision point	Condition and action
1. Preliminary	Data aggregation	Aggregate multiple trials per scenario (mean $F_1$ Score / median Training Time)
2. Statistical test selection	Normality test	If number of results $< 50$, use Shapiro-Wilk^104^; else use Kolmogorov–Smirnov^112^.
	Primary test	If normality violated ($p < 0.05$), use Friedman test^113^; else proceed to Mauchly’s Sphericity test^114^.
	Sphericity test	If sphericity violated ($p < 0.05$), use RM-ANOVA^115^ with Greenhouse-Geisser correction^116^; else use RM-ANOVA without correction.
	Failure handling	If any test produces errors/NaN, fallback to Friedman test.
3. Post-hoc analysis	For Friedman Test	If comparisons $\ge 10$, use Nemenyi^119^; else use Conover^118^ with Holm-Bonferroni correction^111^.
	For RM-ANOVA	If comparisons $\ge 10$, use pairwise *t*-tests with Benjamini-Hochberg correction^117^; else use pairwise *t*-tests with Holm-Bonferroni correction.
4. Visualization	Rank visualization	Compute average ranks and generate bar charts/plots.

This table outlines the normality checks, sphericity checks, primary test choices (Friedman or RM-ANOVA), post-hoc methods (Nemenyi, Conover, or t-tests with corrections), and visualization approaches to derive scenario-wide rankings.

#### Results

Table 14 presents the statistical test results for $F_1$ Score and Training Time when performance is aggregated across all datasets within each scenario. Each row corresponds to a scenario–metric combination, indicating which normality test was performed (Shapiro–Wilk or Kolmogorov–Smirnov), whether the data passed normality ($p \ge 0.05$), the outcome of Mauchly’s sphericity test if applicable, the final primary test chosen (Friedman or RM-ANOVA), and the post-hoc method. The resulting $p$-values confirm statistically significant differences among frameworks ($p < 0.05$) for every scenario and metric.

**Fig. 16:**
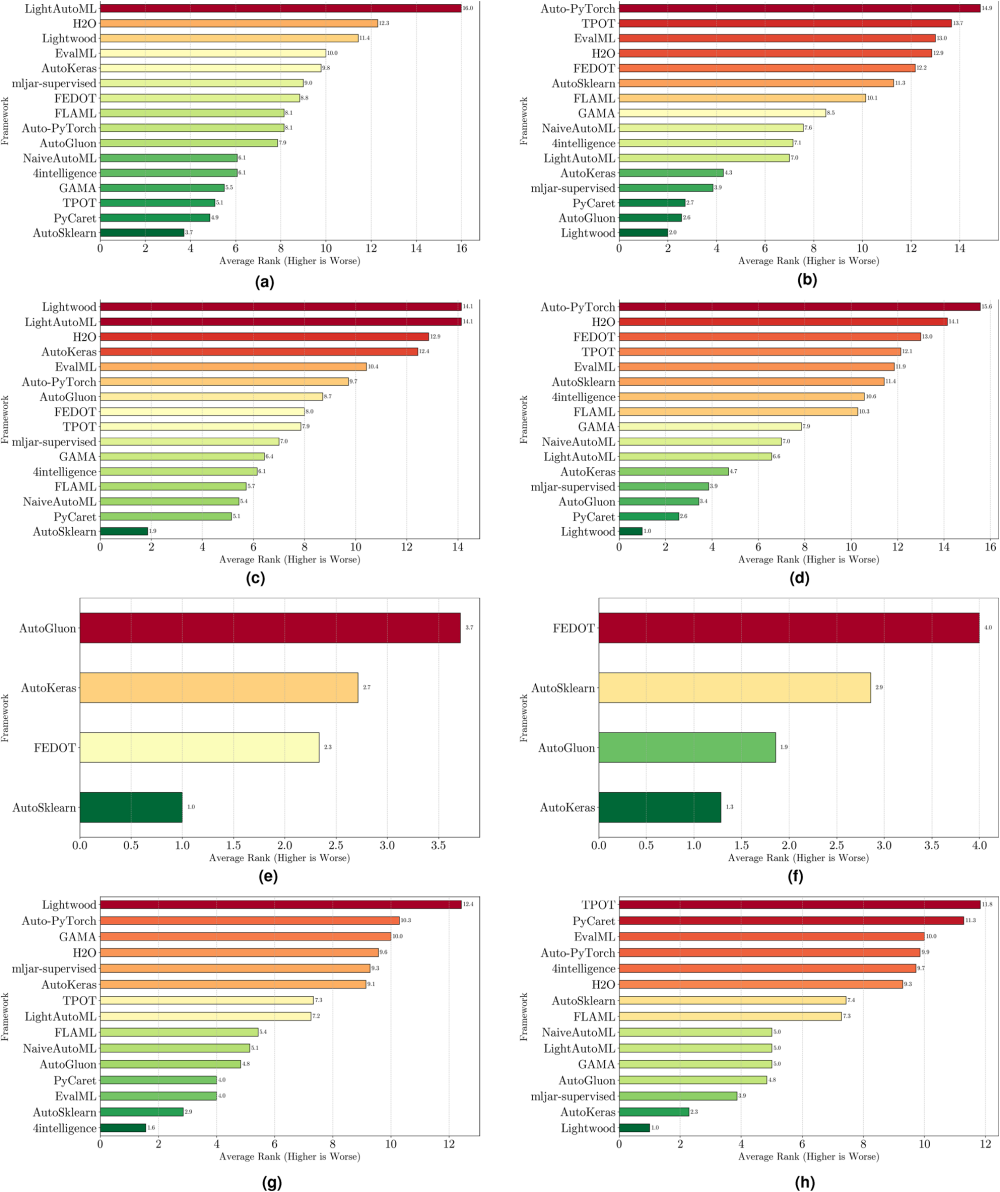
Average ranks for each framework in $F_1$ Score (left subplots) and Training Time (right subplots) across the four scenarios (Binary, Multiclass, Multilabel Native, and Multilabel Powerset). Each pair of subplots corresponds to one scenario, illustrating which frameworks tend to rank higher (lower rank value) or lower (higher rank value) when results from multiple datasets are aggregated.

After applying the appropriate statistical test, we computed each framework’s average rank within each scenario. Figure 16 presents these rankings for $F_1$ Score and Training Time across the binary, multiclass, multilabel (native), and multilabel (powerset) scenarios, offering a comparative view of framework consistency within each scenario.

**Table 14:** Statistical test results across datasets for $F_1$ score and training time.

Metric	Scenario	Normality test	Normality *p*-value	Normal?	Sphericity *p*-value	Spherical?	Parametric?	Method	*p*-value	Significant?
$F_1$ Score	Binary	Shapiro–Wilk	$7.1 \times 10^{-15}$	No	$3.3 \times 10^{-200}$	No	No	Friedman + Nemenyi	$4.8 \times 10^{-06}$	Yes
	Multiclass	Shapiro–Wilk	$2.7 \times 10^{-15}$	No	$9.0 \times 10^{-154}$	No	No	Friedman + Nemenyi	$3.8 \times 10^{-07}$	Yes
	Multilabel (Native)	Shapiro–Wilk	$6.3 \times 10^{-02}$	Yes	$4.4 \times 10^{-06}$	No	No	Friedman + Conover + Holm	$2.9 \times 10^{-03}$	Yes
	Multilabel (Powerset)	Shapiro–Wilk	$6.2 \times 10^{-09}$	No	N/A	N/A	No	Friedman + Nemenyi	$1.7 \times 10^{-03}$	Yes
Training Time	Binary	Shapiro–Wilk	$2.9 \times 10^{-10}$	No	$1.0 \times 10^{-213}$	No	No	Friedman + Nemenyi	$5.7 \times 10^{-11}$	Yes
	Multiclass	Shapiro–Wilk	$1.0 \times 10^{-10}$	No	$0.0 \times 10^{+00}$	No	No	Friedman + Nemenyi	$2.4 \times 10^{-13}$	Yes
	Multilabel (Native)	Shapiro–Wilk	$6.3 \times 10^{-02}$	Yes	$1.8 \times 10^{-08}$	No	No	Friedman + Conover + Holm	$9.4 \times 10^{-04}$	Yes
	Multilabel (Powerset)	Shapiro–Wilk	$2.8 \times 10^{-12}$	No	N/A	N/A	No	Friedman + Nemenyi	$1.6 \times 10^{-04}$	Yes

The Friedman test was employed for scenarios where normality or sphericity were not satisfied, accompanied by an appropriate post-hoc (Nemenyi or Conover+Holm). If normality was held but sphericity failed (or Mauchly’s test returned NaN), the procedure was also reverted to Friedman.

Overall, these results indicate that framework choice significantly impacts both the $F_1$ Score and Training Time across all scenarios (binary, multiclass, and both multilabel modes). The statistical tests (Friedman or RM-ANOVA, when applicable) provided strong evidence of these differences. The subsequent average-rank plots (Fig. 16a–h) highlight the top- and bottom-performing frameworks across aggregated datasets.

#### Statistical interpretation

Before addressing the research questions for the across-datasets analysis, it is useful to recall that these results aggregate each framework’s performance across all datasets belonging to a particular scenario. Thus, the mean $F_1$ Score and median Training Time serve as single representative values per dataset, allowing a scenario-wide comparison that may differ from the conclusions drawn at the per-dataset level.

RQ4: Do framework performance differences remain statistically significant across datasets within each scenario?

Yes. As shown in Table 14, every scenario (binary, multiclass, and multilabel in both native and powerset forms) exhibits significant differences ($p < 0.05$) for both $F_1$ Score and Training Time. The *Multiclass* scenario shows especially pronounced variations, particularly in Training Time ($p = 2.4 \times 10^{-13}$) and, to a slightly lesser extent, $F_1$ Score ($p = 3.8 \times 10^{-7}$). Similarly, the *Binary* scenario registers considerable discrepancies ($p = 5.7 \times 10^{-11}$ for Time, $p = 4.8 \times 10^{-6}$ for $F_1$ Score).

By contrast, both *Multilabel (Native) and Multilabel (Powerset)* scenarios show statistically significant yet smaller-effect differences (e.g., $p \approx 10^{-3}$). This implies that although frameworks remain distinguishable, their relative performance is somewhat more interchangeable in multilabel tasks than in binary or multiclass classification. Notably, Training Time divergences are consistently more extreme than those observed for $F_1$ Score, suggesting framework choice impacts computational efficiency even more strongly than predictive performance.

RQ5: Which frameworks perform best and worst across datasets within each scenario?

The statistical test results (Table 14) and the average-rank plots (Fig. 16) confirm that certain frameworks dominate in $F_1$ Score, while others excel in Training Time. *Binary* tasks see 4intelligence and AutoSklearn leading on $F_1$ Score, whereas Lightwood and AutoKeras are fastest. Conversely, Lightwood and Auto-PyTorch trail in $F_1$ Score, while TPOT and PyCaret are the slowest. In *Multiclass* tasks, AutoSklearn dominates $F_1$ Score, followed by FEDOT and AutoKeras, whereas AutoGluon ranks last. For Training Time, AutoKeras is fastest, but FEDOT is consistently slower. Within *Multilabel (Native)*, AutoSklearn stands out in $F_1$ Score, while Lightwood leads in speed, and in *Multilabel (Powerset)*, AutoSklearn still achieves the top $F_1$ Score rank, but LightAutoML lags behind. Meanwhile, Lightwood repeats its speed advantage, with Auto-PyTorch and TPOT trailing behind.

RQ6: How do the results from the across-datasets analysis compare to the per-dataset analysis?

Many key patterns remain consistent, such as AutoSklearn ’s strong $F_1$ Score and Lightwood ’s speed advantage, yet ranking disparities emerge once multiple datasets are aggregated. For instance, 4intelligence excels in binary tasks on a per-dataset basis but drops in the aggregated standings, indicating potential dataset-specific dependencies. Conversely, H2O improves upon aggregation despite weaker single-dataset results, suggesting scenario-specific variability. These shifts highlight how some frameworks perform well under certain conditions but less so when many datasets are considered simultaneously.

Table 15 summarizes these comparisons by listing the best and worst frameworks in both the per-dataset and across-datasets analyses. While aggregation provides a broader perspective within each scenario, some frameworks remain consistently strong (shown in Bold), while others persist as weaker performers (shown in Italic). However, robust frameworks in one setting may show inconsistencies in another, reinforcing the importance of evaluating performance across different aggregation levels.

**Table 15:** Comparison of best and worst frameworks between per-dataset and across-datasets analyses.

Metric	Scenario	Best frameworks		Worst Frameworks	
		Per-dataset	Across-datasets	Per-dataset	Across-datasets
$F_1$ score	Binary	**4intelligence**, **AutoSklearn**	**AutoSklearn**, **4intelligence**	*LightAutoML*	*Lightwood*, Auto-PyTorch
	Multiclass	**AutoSklearn**, PyCaret	**AutoSklearn**, FEDOT, AutoKeras	H2O, *LightAutoML*, Lightwood	*AutoGluon*
	Multilabel (Native)	**AutoSklearn**	**AutoSklearn**	*AutoGluon*	*Lightwood*, LightAutoML
	Multilabel (Powerset)	**4intelligence**	**AutoSklearn**, PyCaret	*Lightwood*, H2O	*LightAutoML*
Training time	Binary	**Lightwood**, AutoGluon	**Lightwood**, AutoKeras	Auto-PyTorch, H2O	*TPOT*, *PyCaret*
	Multiclass	**Lightwood**, PyCaret	**AutoKeras**, AutoGluon	Auto-PyTorch, H2O	*FEDOT*
	Multilabel (Native)	AutoKeras	**Lightwood**	*FEDOT*	*Auto-PyTorch*
	Multilabel (powerset)	**Lightwood**, AutoKeras	**Lightwood**, AutoGluon, PyCaret	PyCaret, *TPOT*	*Auto-PyTorch*, *TPOT*

Frameworks shown in Bold consistently rank among the best, whereas those shown in Italic remain among the worst.

The across-datasets analysis offers valuable insights, but it does not establish cross-scenario robustness. The upcoming *all-datasets* analysis (“All-datasets analysis” section) further consolidates every task and both metrics, aiming to identify frameworks that balance predictive performance and computational efficiency across all classification types.

### All-datasets analysis

To derive an overall ranking of frameworks across *all* classification scenarios, we combined every dataset—binary, multiclass, and both multilabel modes—into one unified analysis. Because each dataset-framework combination constitutes a repeated measure, we first tested sphericity. As shown in Table 16, the sphericity assumptions failed, necessitating a non-parametric approach rather than RM-ANOVA.

Following the procedure used in the across-datasets analyses (“Across-datasets analysis” section), we employed the Friedman test to assess whether frameworks differed significantly in both $F_1$ Score and Training Time when evaluated over all datasets. Upon finding significant results ($p<0.05$), pairwise comparisons were conducted with Nemenyi’s post-hoc test to pinpoint which frameworks were meaningfully distinct. We then calculated each framework’s average rank (from the Friedman procedure) to form a final, comprehensive ranking encompassing all tasks.

**Fig. 17:**
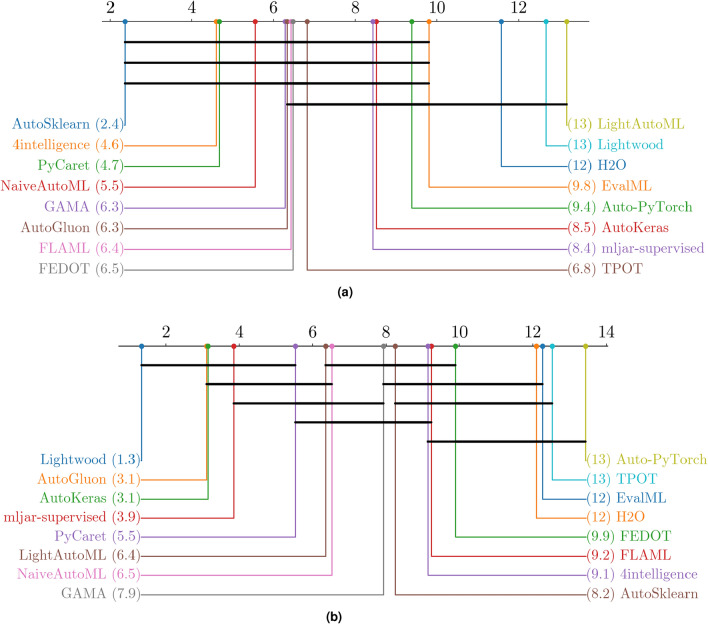
Critical difference diagrams for framework performance in terms of $F_1$ score and training time.

To illustrate these results, we generated Critical Difference (CD) diagrams for both $F_1$ Score and Training Time (Figs. 17a,b). These diagrams concisely show how frameworks cluster together (or stand apart) according to average ranks. Additionally, we plotted a dual-rank scatter (Fig. 19), which shows each framework’s relative position regarding predictive performance versus computational efficiency. This visualization helps readers quickly identify which frameworks strike the best trade-off between high $F_1$ Scores and low Training Times when every dataset, regardless of classification type, is considered.

#### Results

Table 16 summarizes the statistical outcomes. The Shapiro-Wilk test for normality indicated a pronounced deviation ($p \ll 0.05$), and Mauchly’s test confirmed sphericity violations ($p=0$), leading us to the Friedman test for both metrics. Post-hoc Nemenyi analyses showed clear differences among frameworks ($p=1.6 \times 10^{-5}$ for $F_1$ Score and $p=1.8 \times 10^{-29}$ for Training Time), indicating that the choice of framework remains highly impactful even when all tasks are considered jointly.

**Table 16:** Statistical test results across all datasets for $F_1$ score and training time.

Metric	Scenario	Normality test	Normality $p$-value	Normal?	Sphericity $p$-value	Spherical?	Parametric?	Method	${p}$-value	Significant?
$F_1$ Score	All datasets	Shapiro–Wilk	$1.5 \times 10^{-21}$	No	0.0	No	No	Friedman + Nemenyi	$1.6 \times 10^{-05}$	Yes
Training Time	All datasets	Shapiro–Wilk	$1.2 \times 10^{-22}$	No	0.0	No	No	Friedman + Nemenyi	$1.8 \times 10^{-29}$	Yes

**Fig. 18:**
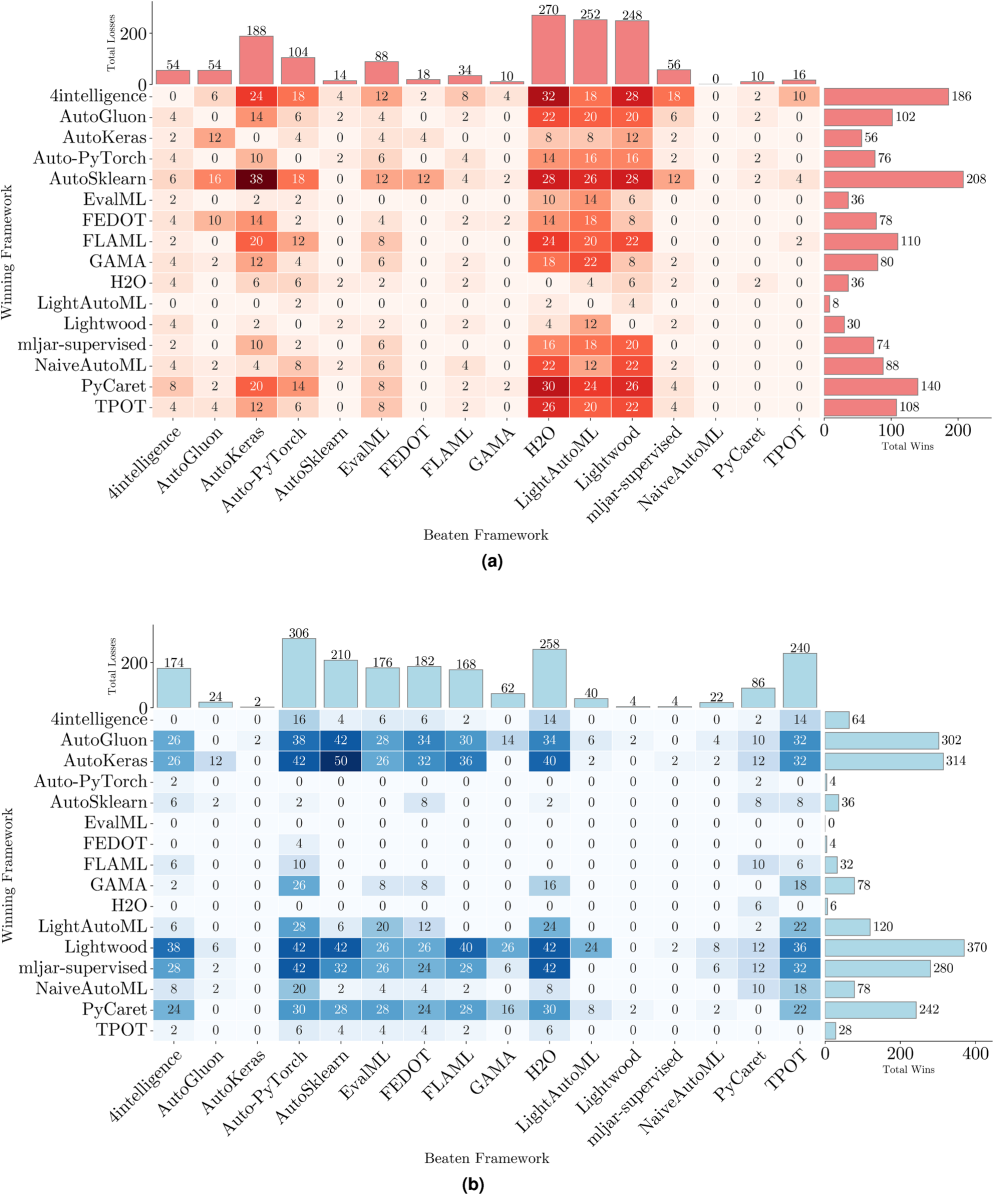
Pairwise win–loss matrices showing how often each framework outperformed others across all datasets in terms of $F_1$ score and training time. Marginals indicate total wins and losses per framework.

Figures 17a and 18a display the Critical Difference diagrams for $F_1$ Score and Training Time, respectively. Both diagrams illustrate that certain frameworks group together statistically, whereas others sit far apart, reflecting greater relative differences.

To complement the statistical tests and provide a finer-grained perspective, we visualized the raw pairwise win–loss counts for each framework across all datasets (Fig. 18). Each cell in the matrix shows how often the framework in the row outperformed the one in the column in terms of $F_1$ Score (Fig. 18a) and Training Time (Fig. 18b). Marginal bar plots summarize the total number of wins (right) and losses (top), offering a clear snapshot of overall competitiveness. This matrix highlights dominant frameworks that tend to underperform relative to others. By directly counting wins, this view complements the rank-based CD diagrams with a more interpretable metric of comparative dominance.

**Fig. 19:**
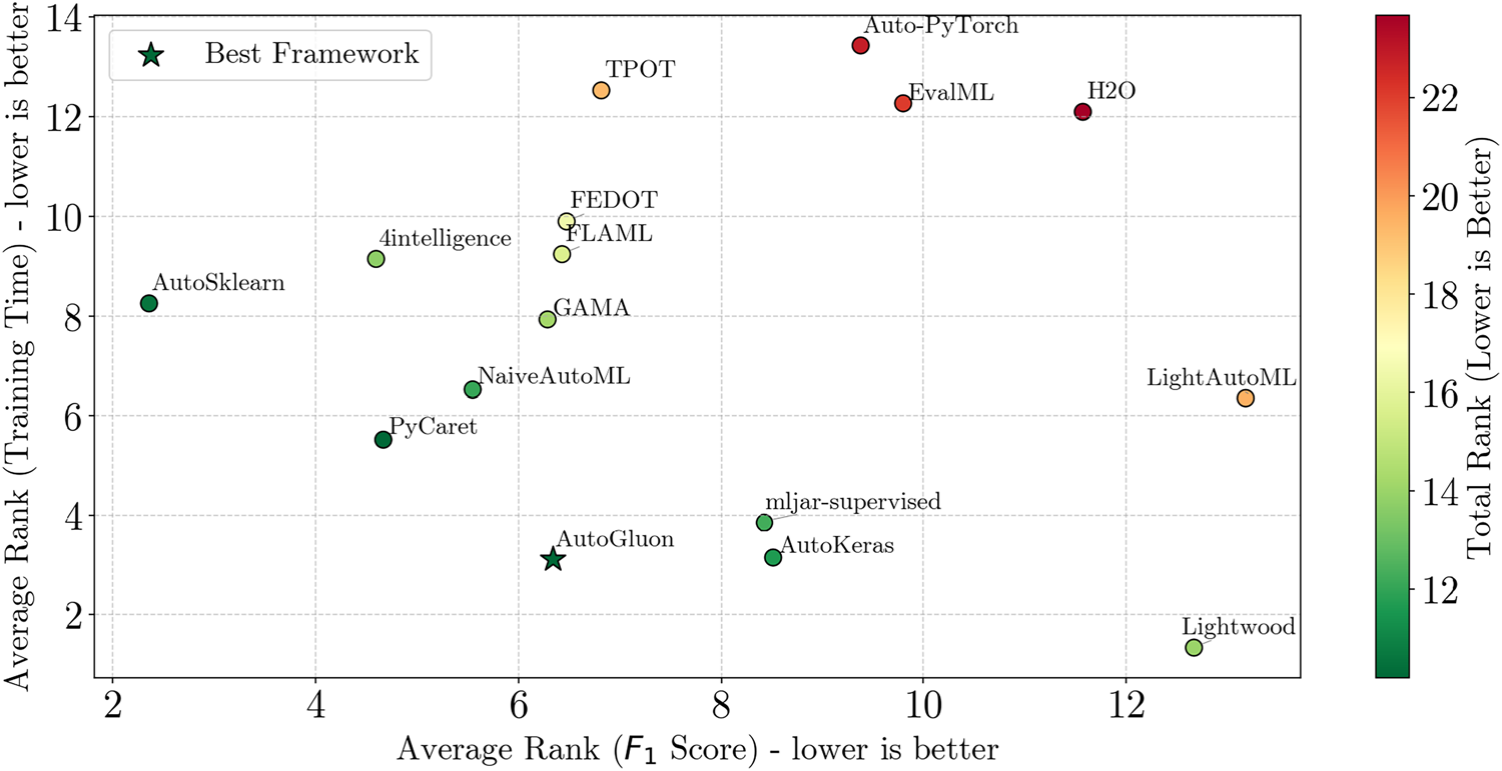
Summary of framework performance across all datasets.

Finally, Fig. 19 shows a dual-rank scatter plot in which each framework’s rank on $F_1$ Score (horizontal axis) is plotted against its rank on Training Time (vertical axis). Frameworks located toward the lower-left corner attain consistently high $F_1$ Score values and low training durations, thus striking an advantageous balance.

With the overall framework rankings established, we now examine their statistical significance to determine whether the observed differences are robust and meaningful.

#### Statistical interpretation

RQ7: Do framework performance and computational efficiency differences remain statistically significant when considering all datasets together?

When all datasets—across binary, multiclass, and multilabel tasks—are pooled into a single unified analysis, it is essential to determine whether frameworks still exhibit meaningful performance distinctions in both predictive performance ($F_1$ Score) and computational efficiency (Training Time). We, therefore, conducted normality checks (Shapiro–Wilk) and tested for sphericity (Mauchly’s test). As shown in Table 16, both metrics deviated strongly from normality ($p \approx 10^{-21}$ for $F_1$ Score, $10^{-22}$ for Time), and sphericity violations ($p=0$) confirmed that *neither* a parametric nor sphericity-based approach (e.g., RM-ANOVA) was appropriate.

Accordingly, we used the Friedman test. The resulting $p$-values ($1.6 \times 10^{-5}$ for $F_1$ Score and $1.8 \times 10^{-29}$ for Time) indicate that framework selection remains *highly significant* even when every dataset is considered simultaneously. A post-hoc Nemenyi procedure further revealed substantial pairwise differences between certain frameworks. These findings expand upon those from “Across-datasets analysis” section, implying that the impact of framework choice on predictive performance and computational efficiency is not confined to individual tasks or aggregated scenarios but persists across *all* classification settings combined.

RQ8: Which framework achieves the best performance across all scenarios in terms of $F_1$ Score?

We next examined the CD diagram for $F_1$ Score (Fig. 17a), which aggregates each framework’s average rank (from the Friedman test) while also grouping statistically equivalent frameworks. The diagram shows that *AutoSklearn* obtained the best average rank ($\approx 2.4$), signaling excellent predictive performance across the entire dataset corpus. However, post-hoc analysis indicates that AutoSklearn is not strictly better than several close contenders, including 4intelligence ($\approx 4.6$), PyCaret ($\approx 4.7$), and NaiveAutoML ($\approx 5.5$). Moreover, the aggregated win–loss tallies (Fig. 19a) confirm that AutoSklearn achieved 208 wins against only 36 losses in head-to-head $F_1$ comparisons, outpacing 4intelligence (186 wins/54 losses) and PyCaret (140/56), and underscoring its consistent dominance.

Below this top group stands a broader “middle tier,” comprising frameworks like TPOT, FLAML, GAMA, and AutoGluon, each showing competent $F_1$ Scores though lacking the small rank values of the top cluster. Meanwhile, LightAutoML and Lightwood share the worst positions (rank $\approx 13.0$), indicating that these frameworks consistently underperformed regarding raw predictive performance. Thus, although AutoSklearn emerges as a top contender, multiple others (4intelligence, PyCaret, NaiveAutoML) can achieve similar predictive performance, emphasizing that training speed and other practical considerations may further influence framework choice.

RQ9: Which framework is the most efficient across all scenarios in terms of Training Time?

We inspected the Training Time CD diagram (Fig. 18a) to identify the fastest framework overall. In stark contrast to the more crowded ranks for $F_1$ Score, *Lightwood* stands out as the unequivocally fastest (rank $\approx 1.3$), with statistical evidence (Friedman + Nemenyi) confirming its significant lead. Clustering behind it, AutoGluon and AutoKeras (both $\approx 3.1$) form the second-best computational efficiency group, while a mid-tier segment includes mljar-supervised ($\approx 3.9$) and PyCaret ($\approx 5.5$). Another slightly slower band (LightAutoML, NaiveAutoML, GAMA) hovers around ranks 6–8, significantly behind the leaders but not as slow as the bottom group. The pairwise speed heatmap (Fig. 18b) further illustrates that Lightwood recorded 370 wins against just 40 losses in training-time comparisons, vastly exceeding AutoGluon ’s 302/24 and AutoKeras ’s 314/32, thereby quantifying its clear efficiency edge.

Finally, AutoSklearn ($\approx 8.2$), H2O (12), TPOT (13), and Auto-PyTorch (13) fall at the trailing end. These outcomes align with prior observations that Lightwood consistently yields minimal training times, whereas frameworks like TPOT or Auto-PyTorch exhibit much higher computational overhead. From a purely computational efficiency-driven standpoint, Lightwood is the clear winner.

RQ10: Which framework performs best across all scenarios when considering both $F_1$ Score and Training Time?

While separate CD diagrams for $F_1$ Score (Fig. 17a) and Training Time (Fig. 17b) clarify who excels at predictive performance versus computational efficiency, practical deployment often requires a framework that balances both. To visualize this trade-off, Fig. 19 plots each framework’s final rank on $F_1$ Score (x-axis) against its rank on Time (y-axis). The lower-left quadrant denotes frameworks that are simultaneously strong in predictive performance and cost-effective in training.

From this scatter, *AutoGluon* emerges as the most balanced option—outperforming peers with 102 wins vs. 54 losses in $F_1$ and 302 vs. 24 in Training Time—while AutoSklearn and PyCaret trade speed for slightly higher $F_1$, and Lightwood trades accuracy for speed. At the other end, frameworks like Auto-PyTorch, TPOT, EvalML, and H2O lag on both axes, and LightAutoML ’s training overhead diminishes its overall standing. Thus, AutoGluon offers the best compromise for general use, though specific use-cases might still favor the speed of Lightwood or the raw accuracy of AutoSklearn.

### Summary of statistical findings

The statistical analyses conducted in this study—covering per-dataset, across-datasets, and all-datasets comparisons—consistently revealed strong, statistically significant distinctions among AutoML frameworks in both $F_1$ Score (predictive performance) and Training Time (computational efficiency). In simpler tasks such as Binary and Multiclass classification, these differences tended to be especially pronounced, with Training Time variations often exceeding the gaps observed in predictive performance. This underlines the considerable disparities in computational efficiency across frameworks.

In the per-dataset and across-datasets analyses, certain frameworks emerged as clear leaders in either predictive performance or computational efficiency. AutoSklearn regularly appeared at or near the top in $F_1$ Score rankings, and Lightwood consistently delivered the fastest Training Times, albeit at the expense of lower predictive performance. AutoGluon and PyCaret generally struck a balance, maintaining relatively strong ranks on both metrics. Other frameworks, including Auto-PyTorch and LightAutoML, struggled in multiple settings, suggesting more limited applicability for those seeking either top-tier performance or fast training.

When pooling all datasets, head-to-head tallies reinforce these findings: AutoSklearn recorded 208 wins versus 14 losses in $F_1$ comparisons, Lightwood logged 370 wins against 4 losses in speed, and AutoGluon alone achieved net positive records on both axes (102–54 in $F_1$, 302–24 in Time).

Once all datasets were pooled into a single analysis, the results confirmed that AutoSklearn retained its role as one of the most accurate solutions, though it fell behind in speed. Lightwood again stood out for its computational efficiency but continued to post weaker predictive outcomes. AutoGluon, meanwhile, rose to the forefront by combining solid $F_1$ Scores with comparatively low Training Times, making it the most well-rounded choice overall. Less competitive frameworks included Auto-PyTorch, TPOT, and EvalML, which frequently incurred high computational costs while also underperforming on predictive performance.

Taken together, these findings offer practical guidance for selecting an AutoML framework. Users with a strict focus on predictive performance may lean toward AutoSklearn or PyCaret, whereas those needing rapid model training might adopt Lightwood or AutoKeras. For those seeking a balanced compromise, AutoGluon proved to be the strongest general-purpose option, ranking highly in both predictive performance and computational efficiency across the full range of classification tasks.

## Threats to validity

No benchmarking study is immune to methodological or contextual limitations, particularly when comparing complex systems like AutoML frameworks. In this section, we outline the primary factors that may affect the validity, reproducibility, or generalizability of our findings. We separate these into two categories: (1) limitations stemming from the design and scope of the study itself, and (2) emergent biases and behavioral patterns observed during evaluation.

### Scope and design limitations

- The evaluation was conducted using specific *framework versions* available at the time of the study. Because AutoML tools evolve rapidly, results may differ for future releases. Version tracking is essential for reproducibility.
- Although the selected datasets span binary, multiclass, and multilabel tasks, their *domain coverage* remains limited. Important areas such as healthcare, finance, or cybersecurity are underrepresented. Future studies could incorporate more specialized benchmarks to broaden generalizability.
- The *runtime constraint* of 5 min per tool-task pair was chosen to reflect practical deployment settings. However, this may have limited the performance of frameworks designed for long-running or iterative optimization.
- The use of *default configurations* ensured fair and comparable conditions across tools, but may have disadvantaged those that benefit from tuning or expert guidance. Custom parameterization could yield different performance outcomes.
- Our primary evaluation metrics were *weighted* $F_1$ and training time. While widely used, these do not capture complementary concerns such as fairness, interpretability, or energy efficiency.
- A small number of tool–dataset combinations failed due to *compatibility or stability issues*, including timeouts, memory errors, or unsupported feature types. These partial failures may skew aggregate metrics if not properly accounted for.

These limitations reflect deliberate design choices made to ensure fairness and reproducibility across tools. While necessary for methodological clarity, they may constrain how broadly the findings apply, especially in highly specialized or dynamic settings.

### Systematic biases and runtime constraints

In addition to these structural constraints, the evaluation revealed recurring behavioral patterns that arise from the internal design of AutoML frameworks. These are not specific to individual tools but rather reflect systemic tendencies in how AutoML methods handle data, allocate resources, and optimize performance under pressure.

- Many tools made strong *preprocessing assumptions*, expecting numeric inputs or fully cleaned data. Frameworks that lacked robust handling of categorical variables or missing values frequently failed or underperformed when those conditions were not met.
- Differences in *optimization strategy* created trade-offs between accuracy and speed. Exhaustive search methods (e.g., Bayesian or genetic algorithms) often struggled under tight runtime constraints, while lightweight pipelines tended to favor simplicity at the expense of model expressiveness.
- The level of *native support for multilabel classification* varied widely. Several frameworks relied on label-powerset transformations, which introduced class imbalance and label sparsity—particularly problematic in high-cardinality settings.
- We observed notable *hardware sensitivity*, especially for tools optimized around GPU acceleration. These frameworks often experienced performance drops or memory bottlenecks when restricted to CPU-only environments.
- There were clear signs of *modality bias* across tools. Frameworks tailored for image or text classification frequently generalized poorly to tabular problems, and vice versa, due to pipeline assumptions and architecture defaults.
- Despite using 20 replicate runs with different seeds, we observed significant *run-to-run variability* in certain frameworks. Methods relying on stochastic optimization or non-deterministic pipeline generation exhibited inconsistent results even under controlled conditions.

To mitigate these risks, we recommend selecting frameworks based on alignment with the task type and resource environment, validating preprocessing compatibility in advance, and repeating runs to assess stability—particularly for methods using stochastic or non-deterministic algorithms.

Understanding these biases is essential not only for interpreting our results but also for improving the design of future benchmarks. As AutoML continues to evolve, careful attention to tool behavior under real-world constraints will remain critical for producing evaluations that are both fair and actionable.

## Conclusion

This study provides a comprehensive evaluation of AutoML frameworks across binary, multiclass, and multilabel classification tasks, highlighting their strengths, limitations, and trade-offs in predictive performance and computational efficiency. By analyzing datasets of varying complexities, we offer practical insights into framework selection for real-world applications. We also benchmarked our results against four representative prior studies to contextualize our findings within the existing AutoML literature.

Our findings indicate that AutoSklearn and PyCaret achieved the highest $F_1$ scores, albeit with longer training times, while Lightwood and AutoKeras prioritized speed at the expense of predictive performance. A structured summary of these trade-offs is provided in Table 11.

To ensure that these differences were not due to random variation, we conducted a multi-level statistical analysis. The results confirmed significant performance disparities across all classification tasks. In binary classification, frameworks that perform exhaustive HPO—such as AutoSklearn and TPOT—consistently achieved the highest $F_1$ scores but required longer runtimes. Meanwhile, faster frameworks like Lightwood and AutoGluon sacrificed some predictive performance for improved efficiency. A similar trend was observed in multiclass classification, where AutoSklearn and PyCaret achieved the best accuracy, while H2O and LightAutoML exhibited greater variability and, in some cases, slower convergence.

Multilabel classification posed a unique challenge, as few frameworks supported it natively. Among those that did, AutoSklearn delivered the strongest accuracy, whereas AutoKeras prioritized speed at the cost of predictive performance. When using powerset transformations, AutoSklearn remained the most effective, but frameworks such as LightAutoML, Lightwood, and GAMA struggled significantly with sparse label spaces. The all-datasets analysis reinforced these findings, showing that AutoSklearn consistently led in $F_1$ scores but frequently consumed the full training budget, making it less suitable for time-sensitive applications. In contrast, Lightwood was the fastest but least accurate, while AutoGluon emerged as the most well-rounded framework, striking a strong balance between predictive performance and computational efficiency.

Future research should explore meta AutoML frameworks that dynamically optimize framework selection across tasks. Expanding the dataset pool to include domain-specific challenges and integrating techniques like reinforcement or meta-learning could further enhance AutoML adaptability. Ethical considerations, such as bias mitigation and interpretability, remain crucial as AutoML adoption grows.

By addressing these challenges—particularly the need for robust multilabel support and fair, interpretable models—AutoML frameworks can fully realize their potential as universal AI tools. While AutoGluon emerged as the strongest general-purpose solution, users must consider trade-offs between predictive performance, computational efficiency, and framework adaptability based on their specific application needs. All code and analysis scripts are publicly available to ensure full reproducibility and to support future advancements in AutoML.

## Data Availability

Dataset 31—credit-g^63^ is available at OpenML (https://www.openml.org/search?type=data&id=31). Dataset 37—diabetes^64^ is available at OpenML (https://www.openml.org/search?type=data&id=37). Dataset 44—spambase^65^ is available at OpenML (https://www.openml.org/search?type=data&id=44). Dataset 1462—bank-note-authentication^66^ is available at OpenML (https://www.openml.org/search?type=data&id=1462). Dataset 1479—hill-valley^67^ is available at OpenML (https://www.openml.org/search?type=data&id=1479). Dataset 1510—wdbc^68^ is available at OpenML (https://www.openml.org/search?type=data&id=1510). Dataset 40945—titanic^69^ is available at OpenML (https://www.openml.org/search?type=data&id=40945). Dataset 23—contraceptive-method-choice^70^ is available at OpenML (https://www.openml.org/search?type=data&id=23). Dataset 36—segment^71^ is available at OpenML (https://www.openml.org/search?type=data&id=36). Dataset 54—vehicle^72^ is available at OpenML (https://www.openml.org/search?type=data&id=54). Dataset 181—yeast^73^ is available at OpenML (https://www.openml.org/search?type=data&id=181). Dataset 1466—cardiotocography^74^ is available at OpenML (https://www.openml.org/search?type=data&id=1466). Dataset 40691—wine-quality^75^ is available at OpenML (https://www.openml.org/search?type=data&id=40691). Dataset 40975—car^76^ is available at OpenML (https://www.openml.org/search?type=data&id=40975). Dataset 285—flags^77^ is available at OpenML (https://www.openml.org/search?type=data&id=285). Dataset 41464—birds^78^ is available at OpenML (https://www.openml.org/search?type=data&id=41464). Dataset 41465—emotions^79^ is available at OpenML (https://www.openml.org/search?type=data&id=41465). Dataset 41468—image^80^ is available at OpenML (https://www.openml.org/search?type=data&id=41468). Dataset 41470—reuters^81^ is available at OpenML (https://www.openml.org/search?type=data&id=41470). Dataset 41471—scene^82^ is available at OpenML (https://www.openml.org/search?type=data&id=41471). Dataset 41473—yeast^83^ is available at OpenML (https://www.openml.org/search?type=data&id=41473).

## Code availability

All the code and instructions for reproducing the experiments and their outcomes are available at the public GitHub repository (https://github.com/marcelovca90/auto-ml-evaluation).
